# Responses of drinking water bulk and biofilm microbiota to elevated water age in bench-scale simulated distribution systems

**DOI:** 10.1038/s41522-023-00473-6

**Published:** 2024-01-22

**Authors:** Hannah Greenwald Healy, Aliya Ehde, Alma Bartholow, Rose S. Kantor, Kara L. Nelson

**Affiliations:** 1 Department of Civil and Environmental Engineering, University of California, Berkeley, Berkeley, CA USA; 2 Division of Environmental and Ecological Engineering, Purdue University, West Lafayette, IN USA

**Keywords:** Water microbiology, Metagenomics, Biofilms, Pathogens

## Abstract

Reductions in nonresidential water demand during the COVID-19 pandemic highlighted the importance of understanding how water age impacts drinking water quality and microbiota in piped distribution systems. Using benchtop model distribution systems, we aimed to characterize the impacts of elevated water age on microbiota in bulk water and pipe wall biofilms. Five replicate constant-flow reactors were fed with municipal chloraminated tap water for 6 months prior to building closures and 7 months after. After building closures, chloramine levels entering the reactors dropped; in the reactor bulk water and biofilms the mean cell counts and ATP concentrations increased over an order of magnitude while the detection of opportunistic pathogens remained low. Water age, and the corresponding physicochemical changes, strongly influenced microbial abundance and community composition. Differential initial microbial colonization also had a lasting influence on microbial communities in each reactor (i.e., historical contingency).

## Introduction

Management of the microbial quality of drinking water is aimed primarily at minimizing illness caused by waterborne pathogens, minimizing operational issues such as nitrification and corrosion, and maintaining aesthetic quality (e.g., taste, odor, color) for downstream consumers^1^. Maintenance of a disinfection residual in the distribution system is common practice in many countries, including the United States, to protect against regrowth and contamination within drinking water distribution systems (DWDSs), but chlorine dosing and residuals are limited by the potential for disinfectant byproduct (DBP) formation and taste thresholds^2,3^. Compared to free chlorine, application of chloramine as a residual disinfectant limits the formation of DBPs and suppresses *Legionella* but may increase nitrification in DWDSs^4,5^. Water quality may deteriorate during transport in centralized DWDSs, where water age in the United States has been reported to average 1.3 days^6^ but can be as high as 17 days^7^ or even longer when considering premise plumbing. In general, as water age increases and there is a corresponding decay of disinfectant residual, water quality issues become more prevalent, such as increased temperature, formation of DBPs, pathogen intrusion, sediment deposition, metals leaching, and increased bacterial abundance and metabolic activity^8,9^. Traditional monitoring techniques employed by drinking water providers (e.g., heterotrophic plate counts and total coliforms) measure a limited portion of microbial communities and do not provide sufficient insight into microbial impacts of varied DWDS operation^10^.

High-throughput sequencing has allowed for the holistic study of drinking water distribution system microbial communities and enabled the identification of key treatment processes (e.g., filtration^11,12^) and chemical water quality parameters (e.g., nutrient concentrations, disinfectant, temperature^13^) that influence microbial community composition. Water age and disinfectant have been found to be strong factors in shaping absolute bacterial abundance and microbial community composition^7,14^. For example, greater water age (i.e., combined distribution system residence time and home plumbing residence time) was associated with lower diversity and a greater relative abundance of *Mycobacterium avium*^15^. Stagnation as short as 8 h was found to be a significant driving force for shifts in building water bacterial communities^16–19^. Although there are increasing studies in this research area, studies focused specifically on drinking water microbiomes are still fragmentary^20^. Surprisingly, little information is available about eukaryotic communities in drinking water, although there has been a recent push to include eukaryotes and construct eukaryotic MAGs in metagenomic analysis^14,21,22^. Further studies into microbial communities will lend insight into when and why problematic microbiota proliferate in water systems and how to control their growth.

In DWDSs, over 90% of microorganisms are present in pipe wall biofilms where they are better protected from disinfectant residuals and hydraulic shocks by a gelatinous matrix of extracellular polymeric substances^23,24^. Currently, in the United States, most hospitalizations and deaths due to waterborne pathogens are caused by biofilm-associated opportunistic pathogens (e.g., nontuberculous *Mycobacteria*, *Pseudomonas*, *Legionella*), costing $2.39 billion annually^25^. Despite their importance to drinking water microbial ecology, biofilms are often neglected in microbiome studies because of the challenges associated with sampling real-world systems. Simulated distribution systems, such as pipe loops or bench-scale reactors, can provide controlled growth conditions and the ability to sample drinking water biofilms.

The COVID-19 pandemic highlighted the importance of understanding how water age impacts water quality as social distancing measures led to population shifts and changes in water demand. In a survey of practitioners from 28 utilities, 46% mentioned a decrease in commercial demand and 36% experienced reduced overall demand (with one reporting 70% less demand than normal), with some reporting low chlorine residuals or issues maintaining water quality^26^. A study on water use in California found that there was an 11.2% decrease in water demand in the commercial, industrial, and institutional sector^27^. These variations in water demand (and therefore water age) in DWDSs are not confined to the COVID-19 pandemic. For example, water age may increase as population levels decrease in an area due to school breaks on a university campus, during off-season in seasonal communities, during evacuations for natural disasters, or in gradually shrinking populations. As mentioned previously, water age increases as water is transported through the DWDS, and even during typical operation, sections of continuously-supplied DWDSs can be subjected to variable hydraulic conditions, including no-flow conditions at dead-end pipes^28^. On a finer scale, occupancy reductions in individual buildings or limited use of an individual fixture can also increase water age and deteriorate water quality at those points. The studies published to date on microbial water quality impacts of the COVID-19 pandemic focused on water quality and remediation tactics at these consumer end-points, rather than system-wide impacts^5,29–35^.

Using benchtop model distribution systems, we aimed to characterize the impacts of elevated water age on microorganisms in bulk water and pipe wall biofilms. Study reactors were already in operation 6 months prior to building closures related to the COVID-19 pandemic, providing an opportunity to study how increased water age entering the reactors impacted microbial communities. The specific goals of this study were to: (i) Investigate the impacts of water age on bulk water and biofilm microbial abundance, activity, and community composition, (ii) Explore biofilm progression and the relationship between biofilm and bulk water bacterial communities, and (iii) Examine factors contributing to community assembly using five replicate reactors operated in parallel.

## Results

**Fig. 1:**
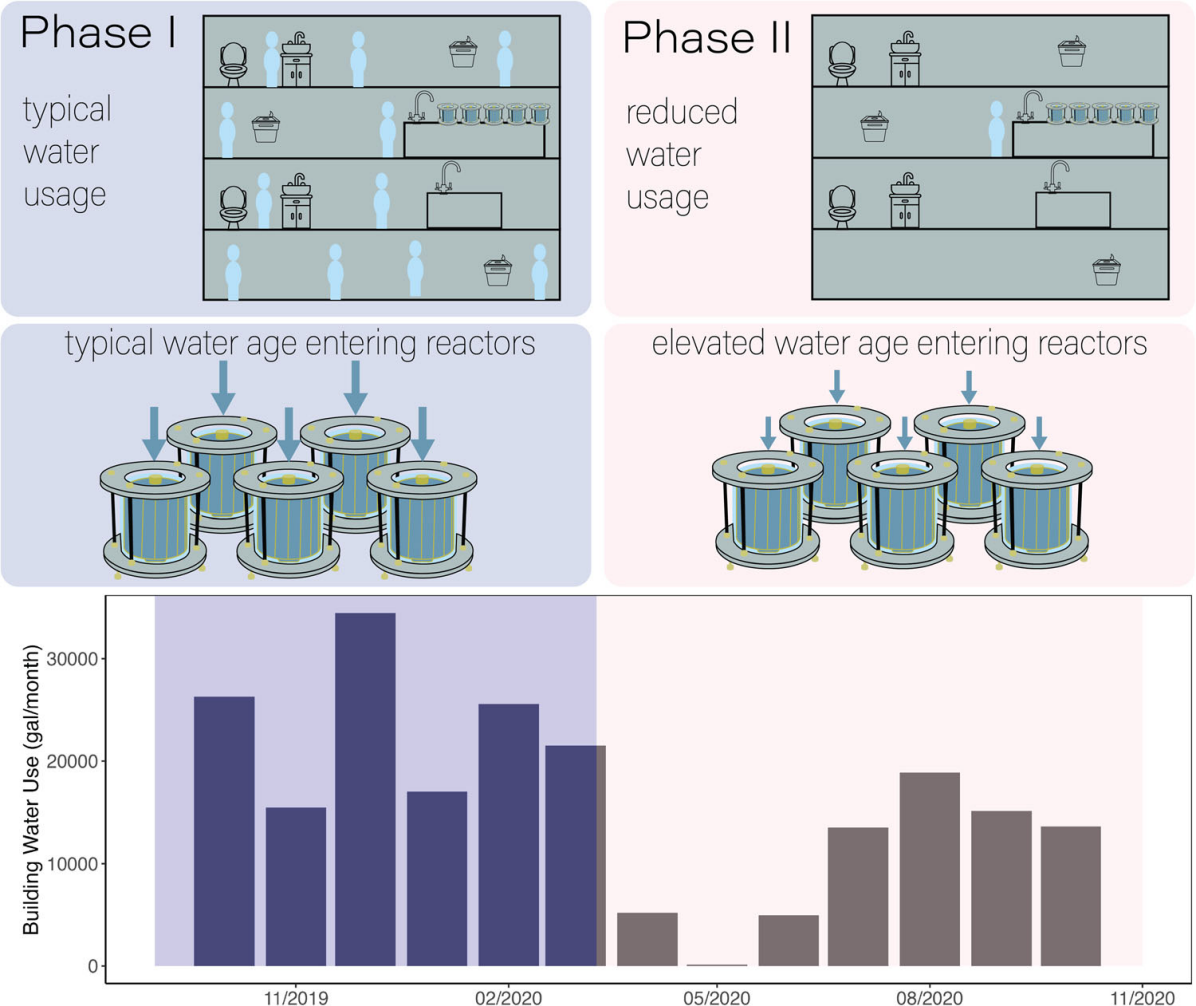
Two phases of the research study. Phase I involved operation of annular reactors being fed by tap water in a building during typical occupancy. In Phase II, the reactor setup was maintained while building occupancy and water use decreased. Although the mean hydraulic residence time in the reactors was the same in both phases, the water age of influent tap water to the reactors was higher in Phase II than in Phase I, leading to overall elevated water age in the reactors. In the bottom panel, building water meter readings are shown.

Five replicate annular reactors were operated in parallel with chloraminated tap water from a municipal drinking water distribution system as feed water (Fig. 1). During the first 6 months of the study (Phase I), building water usage was 23,388 gallons/month, and total chlorine concentration in the tap reservoir was 1.5 mg/L on average (Table 1). When the building was closed to nonessential activity for 7 months (Phase II), usage dropped to 10,210 gallons/month, and total chlorine in the tap reservoir averaged 0.74 mg/L. Phase II comprised 3 months of very low usage (135–5210 gal/month), then 4 months where water usage rebounded to approximately 70% of typical levels (15,300 gal/month on average) when the building reopened at reduced occupancy (Fig. 1). A total of 226 bulk water samples from the annular reactors (referred to as reactors) and reservoir feeding the reactors (referred to as tap reservoir) were collected over 39 sampling events, and 35 biofilm samples were collected over seven sampling events, four before building closure and three after. Cell count and chlorine measurements of samples collected from the tap reservoir feeding the annular reactor versus from the tap directly were not statistically different (Wilcoxon, *p* > 0.05). Therefore, the short residence time in the tap reservoir did not significantly influence water quality (Supplementary Fig. 3), and measurements of tap reservoir samples, not tap samples, are reported throughout. Water from the tap reservoir served as influent to the five replicate annular reactors, and a mean HRT of 8 h was maintained in all reactors throughout the experiment.

**Table 1:** Mean water quality parameters.

	Tap Reservoir		Reactor Bulk		Reactor Biofilm	
	Phase I Mean Conc.	Phase II Mean Conc.	Phase I Mean Conc.	Phase II Mean Conc.	Phase I Final Conc.	Phase II Final Conc.
Total Chlorine	1.5 mg/L	0.74 mg/L	0.62 mg/L	0.11 mg/L	-	-
TCC	5.3 × 10^3^ cells/mL	1.2 × 10^4^ cells/mL	3.9 × 10^4^ cells/mL	1.9 × 10^5^cells/mL	3.2 × 10^5^ cells/cm^2^	1.3 × 10^6^ cells/cm^2^
ICC	9.9 × 10^2^ cells/mL	2.5 × 10^3^ cells/mL	2.2 × 10^4^ cells/mL	1.3 × 10^5^ cells/mL	5.6 × 10^4^ cells/cm^2^	5.6 × 10^5^ cells/cm^2^
ATPt	1.0 × 10^−3^ nM	4.6 × 10^−3^ nM	1.1 × 10^−2^ nM	4.4 × 10^−2^ nM	6.1 × 10^−5^ nmol/cm^2^	4.9 × 10^−4^ nmol/cm^2^
ATPi	4.6 × 10^−4^ nM	7.9 × 10^−4^ nM	7.5 × 10^−3^ nM	3.8 × 10^−2^ nM	1.6 × 10^−5^ nmol/cm^2^	1.8 × 10^−4^ nmol/cm^2^
Temp.	22.6 °C	23.7 °C	22.4 °C	23.5 °C	-	-
pH	7.78	7.69	8.85	8.78	-	-

Mean concentrations of bulk water quality parameters and final concentrations of biofilm parameters in each phase in the tap reservoir and reactors. Geometric means are presented for cell counts and activity, while arithmetic means are presented for all other parameters.

### Bulk water cell counts and activity increased after building closures

**Fig. 2:**
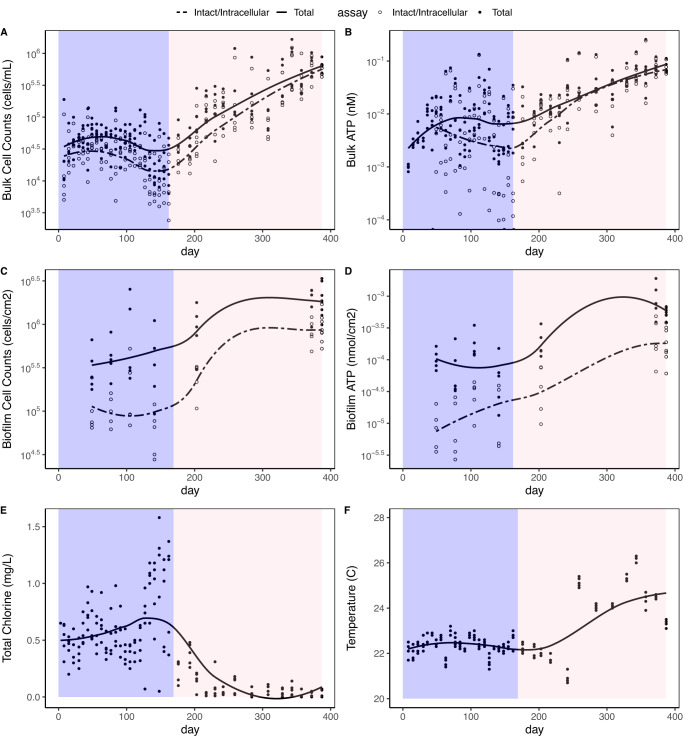
Time series water quality parameters. Time series of total/intact cell counts and total/intracellular ATP in the bulk water (panels **A** and **B**) and in the biofilm (panels **C** and **D**) as well as total chlorine and temperature (panels **E** and **F**) during the two phases of reactor operation in the five replicate reactors. Phases are designated by background colors (purple: Oct. 2019 to mid-March 2020; pink: mid-March 2020 to October 2020).

We characterized the relationships between water age and microbial abundance and activity in our system using flow cytometry and ATP measurements. In the tap reservoir that fed the reactors, mean measurements of TCC and ICC increased from Phase I to Phase II, as did ATP concentrations (Table 1 and Supplementary Fig. 7). Even larger shifts in microbial water quality occurred in the annular reactors between Phases I and II (Table 1 and Fig. 2). Reactors had average ICC concentrations that were 13-fold higher than the tap reservoir in Phase I and 46-fold higher than the tap reservoir in Phase II. The fraction of cells that were intact (ICC/TCC) increased from an average of 56% in Phase I to 67% in Phase II, with a ratio of 80% by the final sampling day. The bulk ratio of intracellular to total ATP (ATPi/ATPt) increased from an average of 64% in Phase I to 86% in Phase II, with a ratio of 93% by the final sampling day. The relatively stable trend in cell counts in Phase I (Fig. 2), when cell counts even declined during a period of high chlorine, indicates that reactors had sufficient time for conditioning prior to the transition into Phase II.

Changes in microbial abundance between the phases were likely influenced by physicochemical water quality shifts, as observed in prior studies^36,37^. In Phase I, average chlorine levels in the tap reservoir and reactors were 1.5 and 0.62 mg/L, respectively, while in Phase II chlorine concentrations decreased to 0.74 and 0.11 mg/L, respectively. Bulk water ICC had a positive correlation with temperature (Kendall’s tau = 0.21, *p* < 0.05) and negative correlations with chlorine (tau = −0.72, *p* < 0.05) and pH (tau = −0.51, *p* < 0.05) (Supplementary Fig. 8). There were no changes in pH between the phases, but the pH was higher in the tap reservoir than in the reactors throughout the study (Supplementary Fig. 8). There was not a significant correlation between ICC and monthly building water usage based on water meter readings (Supplementary Fig. 9).

### Biofilm microbial abundance increased in phase II despite low area coverage throughout the experiment

Similar to the bulk water, biofilm cell counts and ATP increased about an order of magnitude from the last day of biofilm sampling of Phase I (21 days before building closure) to the final day of Phase II (Table 1 and Fig. 2). Regarding relative microbial abundance in the biofilm versus the bulk water, the fraction of intact cells in the biofilm compared to intact cells counts in the whole reactor (bulk + biofilm) reactor ranged from 25% to 89% (Supplementary Fig. 10). However, the calculated fraction in the biofilm is an underestimate because only biofilm on the PVC portion of the reactor interior were sampled and included in the analysis. During Phase I, the fraction of cells in the biofilm increased, likely due to biofilm growth over time. The biofilm fraction was highest when chlorine was high and lowest during Phase II when chlorine was low, possibly because more cells migrate to and survive in biofilms under the stress of chlorine^38^. The fraction of biofilm cells that were intact (ICC/TCC) increased from an average of 24% in Phase I to 39% in Phase II. Similarly, the fraction of intracellular ATP (ATPi/ATPt) went from an average of 16% in Phase I to 30% in Phase II, with a ratio of 93% by the final sampling day. These fractions in the biofilm are consistently lower than they were in the bulk water, but this discrepancy could be due to properties of the matrix (e.g., high amounts of eDNA in biofilm) or the methods (e.g., cell lysis during biofilm sonication). The ratio of ATPi per intact cell was also consistently much lower in the biofilm than in the bulk water (Supplementary Fig. 11).

**Fig. 3:**
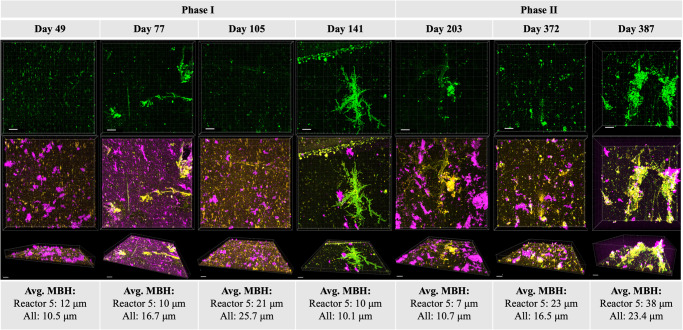
Fluorescent imaging of biofilms. Fluorescent confocal laser scanning microscopy images of drinking water biofilms. (top row) Biofilms stained only with SYTO9 as a projection from above; (second row) Biofilms stained with all three dyes as a projection from above; and (third row) Biofilms stained with all three dyes from an angle. Representative images were chosen from each of seven biofilm sampling events. All images in the figure are of biofilms from the same reactor (reactor 5). MBH stands for maximum biofilm height. Scale bars represent 20 μm. Green: Nucleic acids stained with SYTO9. Orange: Proteins stained with Sypro Orange. Pink: Polysaccharides stained with Concanavalin A, Alexa Fluor 647 conjugate. Yellow: Overlap of green and orange.

Throughout the 13-month experiment, biofilm structure comprised heterogeneous clusters that covered only a small fraction of substrate area (Fig. 3). The mean fractional area coverage was 10.5% with a maximum in a single image view of 47%. This heterogeneity^39,40^ and low area coverage^41^ are commonly observed in drinking water biofilms, including clustered colony formation on PVC^42^. The heterogeneity could also explain why biofilm cell counts were more consistent when larger areas were sampled or imaged (Supplementary Results Section 2). Maximum biofilm height (MBH) ranged from 0 to 78 μm, where the maximum measurement was recorded in reactor 3 on day 387 and seemed to be dependent on both chlorine and time (Supplementary Fig. 12). MBH was weakly correlated to biofilm ICC (Kendall’s tau = 0.1, *p* = 0.03) but not with ATPi. Mean MBH across the full experiment was slightly lower in reactor 2 (10 μm) than in the other reactors (16–19 μm). Similar to MBH, total biovolume increased over the first three sampling events in Phase I, reflecting biofilm growth, but then dropped on the fourth biofilm sampling day when chlorine was high (Supplementary Fig. 13). Unlike height, biovolume did not increase throughout Phase II, possibly reflecting slower recovery of total biomass after high total chlorine concentrations near the end of Phase I. Because of limited dye penetration into large biofilm clusters (biofilms appeared hollow in orthogonal view), biovolume and area coverage were not reliable parameters when biomass was high, which may explain why these parameters are not as high as expected on the final two sampling days when biofilm ICC and ATPi were high. The EPS fraction was consistently high (>90%) but was lowest in the youngest biofilms and during the period of high chlorine (Supplementary Fig. 13).

### Net rates of cell growth and transfer from the biofilm exceeded cell inputs from the tap

Given that cell counts were higher in Phase II in both the tap reservoir and the reactors, we investigated the primary sources of additional cells to the reactor. To estimate relative contributions (to bulk cell counts) of cells from the tap reservoir versus from net growth and transfer from the biofilm, number balances were performed using intact cell counts (ICC). First, a number balance was performed according to Eq. 2, where the system was modeled as a continuously stirred tank reactor with accumulation in the bulk water and biofilm between time points (Supplementary Fig. 6). Because there were no direct measurements for growth/decay rates of cells in the bulk water or rates of transfer of cells from the biofilm to the bulk water, these rates could not be separated and were instead grouped into a single term: the Net Growth/Transfer Rate (NGTR). A second number balance was performed according to Eq. 3, where the system was modeled as a continuously stirred tank reactor at an instantaneous steady state. By assuming there was no accumulation of cells in the reactor during the time frame of one sampling event, the number balance did not rely on biofilm ICC measurements, which resulted in more output data points (*n* = 170) than with Eq. 2 (*n* = 30).

**Fig. 4:**
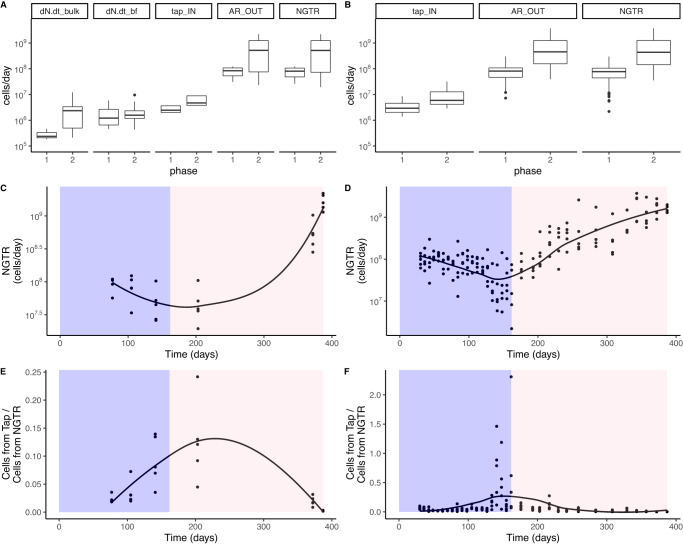
Number balance parameter outputs. **A** Parameters from the Non-Steady State number balance for each phase. **B** Parameters from the Instantaneous Steady State number balance for each phase. **C** Calculated Net Growth/Transfer Rate from the Non-Steady State number balance. **D** Calculated Net Growth/Transfer Rate from the Instantaneous Steady State number balance. **E** Calculated ratio of cell input to the reactor from the tap over input from the Net Growth/Transfer Rate using the Non-Steady State number balance. **F** Calculated ratio of cell input to the reactor from the tap over input from the Net Growth/Transfer Rate using the Instantaneous Steady State number balance. In boxplots, box edges correspond to the first quartile, median, and third quartile. Whiskers extend from the edge to the largest and smallest values no further than 1.5× the interquartile range from the edge. Data beyond the whiskers are considered outliers and plotted individually.

Both number balances showed similar trends in calculated parameters (Fig. 4). In Phase I, the Net Growth/Transfer Rate was relatively stable but decreased slightly during the period when chlorine was high. In Phase II, the NGTR increased from below 10^8^ to above 10^9^ cells/day. Compared to the input of cells from growth and transfer within the reactor, the input of cells from the influent flow from the tap reservoir was low: an average of 5% in the non-steady state number balance and 10% in the instantaneous steady state number balance. Although generally low, this ratio of cell inputs from the tap reservoir over the NGTR was highest at timepoints with high chlorine, meaning contributions of cells from the influent could be more influential during periods of decay and low growth. Overall, these findings suggest the importance of cells already present in the reactor in determining future communities, especially during periods of high growth.

### Variability across the five replicate reactors

**Fig. 5:**
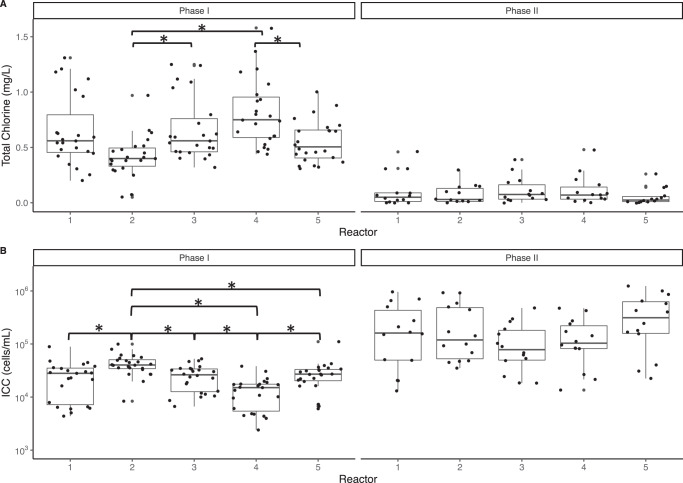
Boxplots by annular reactor. Boxplots of (**A**) total chlorine and (**B**) intact cell counts (ICC) in each reactor in each phase. Brackets with asterisks indicate that mean concentrations were statistically different (Kruskal–Wallis followed by pairwise Wilcoxon rank sum test with a significance threshold of 0.05). Box edges correspond to the first quartile, median, and third quartile. Whiskers extend from the edge to the largest and smallest values no further than 1.5× the interquartile range from the edge. Data beyond the whiskers are considered outliers and plotted individually.

By running five annular reactors in parallel, we were able to analyze variability across reactors with the same operational setup and feed water. In general, the reactors displayed similar trends in chlorine and cell counts over time (Supplementary Figs. 14 and 15). However, despite being operated with the same conditions and influent water in parallel, the concentrations of chlorine across some of the reactors in Phase I were significantly different (Kruskal–Wallis, *p* = 0.04) as were ICC concentrations (Kruskal–Wallis, *p* = 0.002). Reactors with significantly lower chlorine had higher cell counts and vice versa (Fig. 5). These differences could have been due to slight operational differences in the wear of inner elements of the reactor over the 13 months of the experiment or the pump flow rate of water into the reactors. Flow rates were measured and adjusted weekly, but differences in flow rate between adjustments could have led to slightly varied water age. In Phase II, chlorine decreased across all reactors, and chlorine and cell counts were no longer significantly different. Further analysis of variation of bulk and biofilm parameters across reactors is included in Supplementary Results Section 2.

### Microbial taxonomy overview

To explore the relationship between water age and microbial community dynamics, metagenomic sequencing was performed on a total of 4 tap reservoir, 20 bulk, and 10 biofilm samples (Supplementary Table 8). Sequencing resulted in an average of 10,957 Mbp per sample (Supplementary Table 8), an average assembly length of 350 Mbp (Supplementary Table 9), and 83 sample-derived dereplicated metagenome assembled genomes (MAGs). MAGs were named based on taxonomy determined by Anvi’o, followed by a number to distinguish multiple MAGs with the same taxonomy (Supplementary Tables 10 and 11). Overall, the majority of MAGs were classified within the phylum *Proteobacteria* (average percent abundance: 65.9%) followed by *Nitrospirota* (12.4%) and *Actinobacteriota* (9.1%), typical for drinking water^36,38^. Alpha diversity (Shannon Index) was similar in reactor samples from both phases and the tap reservoir from Phase II but higher in the tap reservoir samples from Phase I (results were not significant due to small sample size; Kruskal–Wallis, *p* = 0.26) (Supplementary Fig. 16). The dominant genera in the tap reservoir were *Methylobacterium* (19.5%), *Nitrosomonas* (16.9%), *Nitrospira* (8.5%), *Sphingomonas* (8.5%), and *Mycobacterium* (6.7%). In contrast, the dominant genera in reactor bulk water were *Hyphomicrobium* (18.7%), *Mycobacterium* (12.4%), *Reyranella* (11.4%), *Nitrosomonas* (9.3%), and *Nitrospira* (7.3%). The reactor biofilm also included *Hyphomicrobium* (22.3%), a higher percentage of *Nitrospira* (25.6%), and *Reyranella* (7.2%) (Supplementary Figs. 17–19).

### The tap reservoir community contributed to the reactor community

**Fig. 6:**
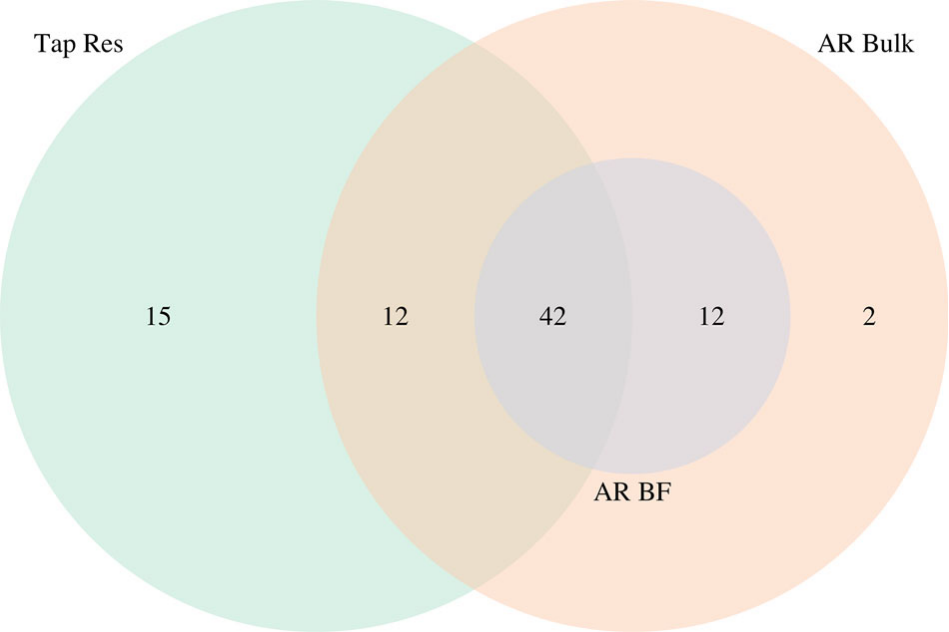
MAGs venn diagram. Venn Diagram showing detection of the 83 dereplicated, sample-derived MAGs in each sample type.

While the majority (*n* = 54 of 83) of MAGs were detected in at least one sample from both the tap reservoir and reactor bulk water (Fig. 6), the community profiles of these samples were different. Fifteen MAGs were detected only in the tap reservoir. Tap reservoir samples also had a higher percentage of reads that did not map to a MAG (Supplementary Table 8), consistent with the higher diversity observed during Phase I. Conversely, fourteen MAGs were detected in the reactors but not in the tap reservoir, although these may have been present in the tap reservoir but not detected due to insufficient sequencing depth.

Even where overlaps occurred, frequency of detection and relative abundances differed between the tap reservoir and reactors. Four MAGs were detected consistently in the reactors but were only detected in the tap reservoir on the final sampling day (Fig. 8). Several other MAGs detected in both the tap reservoir and reactor bulk water had different relative abundances. For example, Gallionella_1, Mycobacterium_2, Obscuribacteraceae_3, Rhodocyclaceae_2 were at moderate relative abundance in the tap reservoir throughout but at low abundance in the reactors in Phase I and were not detected in Phase II.

### Microbial communities shifted as water age increased

**Fig. 7:**
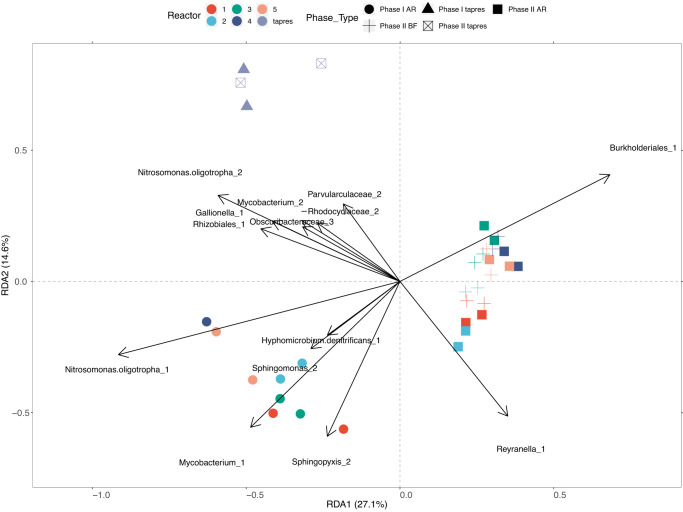
RDA Plot of Key MAGs. Ordination based on redundancy analysis (RDA) of all sequenced samples based on MAG relative abundance. Colors represent the sample source (reactor number or tap reservoir), and shapes represent sample type (AR: reactor bulk, BF: reactor biofilm, tapres: tap reservoir) and phase (1: Phase I, 2: Phase II). Sample AR4_162 is not included due to suspected cross-contamination (see Section “Sequencing Negative Controls and Cross-Contamination”). For readability, arrows and labels for MAGs significant to the RDA were only included if r > 0.63.

The increases in cell counts from Phase I to Phase II and from the tap reservoir to the reactors were associated with shifts in microbial community composition. Microbial communities in the reactors clustered by phase (before and after building closure; PERMANOVA, R^2^ = 27.2%, *p* = 0.001; Fig. 7). Visually, tap reservoir samples clustered separately from reactor samples via redundancy analysis ordination (RDA; Fig. 7 and Supplementary Figs. 20, 21), and several MAGs appeared to drive this clustering, including those identified as Nitrosomonas oligotropha_2, Mycobacterium_2, Obscuribacteraceae_3, and Parvularculaceae_2 (Fig. 7). A separate PERMANOVA conducted to compare reactor samples showed that both phase and sample source (i.e., reactor replicate) significantly shaped community composition (R^2^ = 20.5%, *p* = 0.001 and R^2^ = 29.1%, *p* = 0.003, respectively). These clustering patterns were observed when analyzing both dissimilarity matrices of MAGs and MASH distances between reads (Supplementary Fig. 5). Additionally, the sample source explained a higher percentage of variation in Phase II than in Phase I (Supplementary Table 12).

**Fig. 8:**
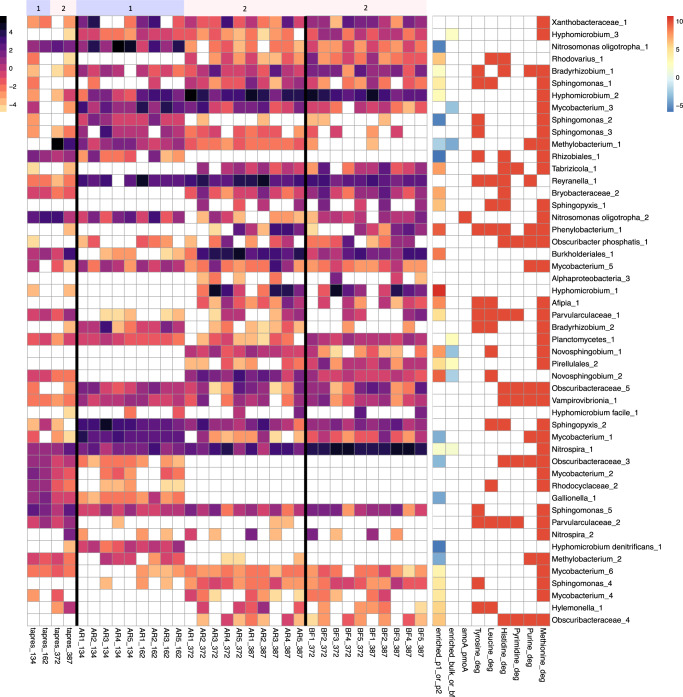
Heatmaps of MAGs. Normalized coverages (log2) of a subset of 50 MAGs. The subset contains MAGs that were either (i) one of the top 10 most abundant MAGs in each sample, (ii) significantly differentially enriched in the reactor or tap reservoir bulk water across phases, or (iii) significantly differentially enriched in reactor bulk water versus biofilm. The top panel represents the phase of the samples. The left heatmap and legend display normalized coverage. The right heatmap and legend display enrichment and presence of key functional pathways. The column ‘enriched_p1_or_p2’ is the log2-fold-change between the phases of significantly enriched bins, where <0 is enriched in Phase I and >0 is enriched in Phase II. The column ‘enriched_bulk_or_bf’ is the log2-fold-change between the sample types of significantly enriched bins, where <0 is enriched in bulk water and >0 is enriched in biofilm. The following columns are presence/absence of KO# in each bin related to *amoA*/*pmoA* or complete KEGG modules related to degradation of amino and nucleic acids. Sample AR4_162 is not included due to suspected cross-contamination (see Section “Sequencing Negative Controls and Cross-Contamination”). A heatmap of all 90 MAGs is included as Fig. S. 23.

Differences in microbial communities by phase were driven by several key taxa. Via differential abundance analysis, 27 MAGs were significantly enriched in Phase II over Phase I, and six MAGs were enriched in Phase I (Fig. 8 and Supplementary Fig. 22). Differential abundance analysis was supported by the RDA, which implicated some of the same MAGs (e.g., Burkholderiales_1, Nitrosomonas oligotropha_1, Hyphomicrobium denitrificans_1, Sphingomonas_2, Mycobacterium_1). In the tap reservoir, Methylobacterium_1 was significantly enriched in Phase II and had the highest abundance compared to other community members (Fig. 8).

### Reactor identity had a significant influence on microbial community composition

Because the sample’s source (i.e., reactor replicate or tap reservoir) contributed significantly to variance in the communities (see Section “The Tap Reservoir Community Contributed to the Reactor Community”), we explored community composition comparatively across the reactors. Several taxa were consistently identified in all reactors, including Hyphomicrobium_2, Hyphomicrobium_3, Sphingopyxis_2, and Sphingomonas_5 (Fig. 8). Differences in reactors were defined by specific key taxa. For example, Sphingomonas_2 was present in the tap reservoir and all reactors in Phase I, but was detected only in reactor 2 in Phase II. All reactors had detection of Hyphomicrobium_1 in Phase II, except for reactor 1. Reactors 2 and 5 resembled each other more closely, both containing higher levels of Sphingopyxis_1. Relatedly, reactors 2 and 5 also had the highest mean cell counts over the course of the experiment, while reactors 3 and 4 had the lowest. Reactors 3 and 4 often showed similar patterns, including lower detection of Mycobacterium_3 and Hylemonella_1 and higher levels of Nitrospira_1. Reactors 4 and 5 had the highest occurrence of Parvularculaceae_1, and reactor 5 was the only reactor with detection of Hyphomicrobium facile_1.

### Some taxa were preferentially enriched in either bulk water or biofilm

To explore the relationship between bulk water and biofilm microbial communities in the reactors, bulk and biofilm samples were compared across paired sampling events in Phase II. In Phase II, reactor identity had a strong influence on microbial community composition such that the biofilm samples clustered with bulk water samples from their respective reactors (Supplementary Fig. 21, Supplementary Table 12). Five MAGs (Mycobacterium_3, Novosphingobium_1, Novosphingobium_2, Methylobacterium_1) were significantly enriched in bulk water while three MAGs (Hyphomicrobium_3, Planctomycetes_1, Pirellulales_2) were enriched in the biofilm (differential abundance analysis; Fig. 8). In addition, Nitrospira_1 was more abundant in biofilm samples and appeared to contribute to the variance between bulk and biofilm samples, while Burkholderiales_1 and Reyranella_1 were more abundant in bulk water (Supplementary Fig. 24).

### Functional potential varied by phase

Multiple MAGs were differentially abundant between the phases, and we sought to identify functional genes that may have led to preferential growth of microorganisms in each condition. Using the KEGG database-derived Kofam HMMs and the built-in Anvi’o function anvi-estimate-metabolism, 17,752 modules were identified in the dereplicated bins, and 6556 were 100% complete. Notably, different MAGs identified as *Mycobacterium* and *Hyphomicrobium* were enriched in each phase but no difference in functional potential could be identified. In the set of Phase II- but not in Phase I-enriched MAGs, multiple MAGs contained functional genes related to the degradation of histidine, leucine, tyrosine, and pyrimidine. Complete modules related to the degradation of amino and nucleic acids that were identified in multiple bins are included in Fig. 8. While multiple detected MAGs were identified as nitrifiers, out of 34 samples, only one sample (AR2_134) had a quantifiable concentration of *amoA* of 87.7 gc/L via qPCR, which was supported by estimates using metagenomics where *pmoA*/*amoA* in this sample was approximated as 45.2 gc/L (more in Supplementary Results Section 3).

### Pathogenic Taxa

Out of 34 samples tested via qPCR, all were negative or amplified below the limit of detection for *L. pneumophila*, *Mycobacterium avium* complex (MAC), and *P. aeruginosa*. None of the sample-derived MAGs or SCGs were identified as the genera *Legionella* or *Pseudomonas*. Seven bins were identified as *Mycobacterium*, but only one was identified to the species level: *Mycobacterium iranicum*, which is an opportunistic pathogen^43^ but not a part of MAC. Although the KEGG database contains limited modules related to pathogenicity of specific organisms, a single pathogenicity module (Pertussis pathogenicity signature, T1SS) was identified in bin Rhodocyclaceae_1. Both detected pathogens were only identified in the tap reservoir samples (*n* = 4 of 4), not the reactor samples. Mycobacterium iranicum_1 had maximum and mean concentrations of 6.9 × 10^3^ and 4.0 × 10^3^ cells/mL, respectively. Rhodocyclaceae_1 had maximum and mean concentrations of 5.2 × 10^3^ and 2.6 × 10^3^ cells/mL, respectively.

### Eukaryotic Taxa

Drinking water eukaryotes serve key ecological roles, such as engaging in predator-prey, parasite-host, and mutualistic relationships^44^. Based on the Domain level suggested by Anvi’o using SCGs, nine sample bins were predicted to be eukaryotic in addition to the eukaryotes included in positive controls (Supplementary Table 13). After examining the GC content and size, it was inferred that these nine bins represented three or four unique genomes with unknown taxonomy. In the reactors, several freshwater amoeba were identified by BLAST of 18 S rRNA genes as *Vexillifera bacillipedes*, *Saccamoeba* spp., and *Vannella* spp. Free-living amoebae are of specific interest because they may be human pathogens (e.g., *Acanthamoeba* spp., *Vermamoeba vermiformis*, *Balamuthia mandrillaris*) or serve as hosts for pathogenic microorganisms such as *Legionella* spp. and *Mycobacterium* spp^44^. SILVA classified only one sequence from a sample: *Liliopsida*, a plant, in a tap reservoir sample from week 23 (Supplementary Table 14). Similarly, BLAST identified a sequence derived from a plant in the same sample (Supplementary Table 15). In addition to plants, BLAST also identified sequences in the tap reservoir samples as segmented worms and algae, all organisms or DNA that could have originated from the source water and have been reported in other drinking water publications^44^.

## Discussion

### Influence of water age and corresponding physicochemical parameters on bulk water quality and microbiota

Water age significantly impacted both microbial abundance and community composition of the bulk water. These differences were observed both between experimental phases and between the tap reservoir and reactor samples (Figs. 2, 7 and Supplementary Figs. 7, 8). Elevated water age has previously been linked to elevated microbial abundance and activity as well as changes in community composition^15,18,19,28,35,44–46^. Although an increase in cell counts does not necessarily represent a health hazard, microbial growth can alter water taste, odor, and color, which are leading causes of water quality complaints^47,48^. Increases in microbial abundance and shifts in community composition can also correspond to growth of pathogenic or nuisance organisms^14,31,49^.

We expected that the low chlorine, low nutrient conditions created by high water age would increase the abundance of opportunistic pathogens, which tend to be oligotrophic^50^ and increase in occurrence at higher water ages^15,51–53^. Despite increases in overall microbial abundance, there was no detection of *Legionella pneumophila, Pseudomonas aeruginosa, or Mycobacterium avium* via qPCR and no detection of the genera *Legionella* and *Pseudomonas* via metagenomics (both SCG- and MAG-based analyses). It is unsurprising that *L. pneumophila* was not detected in this study because *Legionella* spp. tend to be suppressed in DWDSs with chloramine as a residual disinfectant^12^, even after the residual has dissipated^5^. Although *Mycobacterium avium* was not detected, multiple MAGs identified as *Mycobacterium* spp. were prevalent, with different MAGs shifting in abundance between the tap reservoir and reactors as well as between phases (Figs. 7 and 8). While elevated water age alone is not enough to lead to growth of opportunistic pathogens in DWDS^4,30^, elevated water age can exacerbate issues with opportunistic pathogens if they were already present in the system. Robust characterizations of opportunistic pathogen occurrence in distribution systems and building plumbing under typical operating conditions could enable identification of plumbing sections of concern under shifting water demand.

The impacts of water age on microbial water quality are mediated by multiple corresponding physicochemical factors, including disinfectant concentration, temperature, and nutrient concentrations. Lower chlorine and higher temperature trends with increasing water age have previously been established in the literature^8,9,54–56^ and are likely the main factors that contributed to the higher microbial growth observed^8,9,35^. Residual disinfectant concentration is often the dominant variable in determining microbial abundance^36^. Chloramine residual can decay with increasing water age due to oxidizing reactions with natural organic matter, exposed surfaces of metal pipes, and nitrification^19^. At lower levels of disinfectant residual, temperature becomes important for determining bacterial abundance^36^. In this study, higher water temperatures could have been due to water temperatures trending towards ambient temperature with higher water age or the seasonal differences between Phase I and Phase II. Regardless, by increasing the rates of reactions, higher temperatures can lead to increased dissolution of metals from pipes thereby increasing conductivity, lead to more rapid loss of dissolved oxygen, and further accelerate the degradation of chlorine residuals^54^. The shift in microbial community composition was likely related to changing growth conditions. As water age increases and assimilable nutrients are consumed by microorganisms, bacteria may need to rely on recycling biomass as a source of nutrients (necrotrophic growth), hence why MAGs with the functional potential to degrade amino acids may have been enriched in Phase II. Other studies have shown enrichment of metabolic modules related to amino acid degradation in disinfected DWDS during high biomass turnover rates^57^. However, differences in functional potential could not be identified between MAGs belonging to the same genera that were differentially enriched across the phases. Overall, a complex set of factors influences microbial community composition, including water age, location of growth, time since initial colonization, sampling source, and physicochemical water quality. These factors are intertwined, and it is difficult to assess their influence on community composition, even in a controlled, replicated study.

### Biofilm progression and relation to bulk water

Relatively few studies have investigated progression of drinking water biofilms, especially for time scales beyond eight weeks. During Phase I, biofilm ICC remained relatively stable between the first measurement (taken at 7 weeks) and the 6-month mark (Fig. 2), indicating that a development phase of seven weeks was sufficient to establish a stable biofilm on the basis of cell counts. Throughout the experiment, microscopy revealed that biofilm structures were relatively thin and heterogeneous, similar to previous drinking water biofilm studies on PVC substrate^42^. The ratios of intact/total cells and intracellular/total ATP were consistently lower in the biofilm than bulk water, as was the ratio of ATP per cell (Supplementary Fig. 11), which could indicate higher growth and activity of cells in the bulk water than the biofilm or a net transfer of intact, high-activity cells from the biofilm into the bulk water. Previous studies have also noted that the bulk water phase bacteria exhibited a higher activity than the biofilm bacteria in terms of culturability and cell-specific ATP content^58^.

It was unclear how biofilm would respond to elevated water age, especially while maintaining consistent shear forces. Shear forces have been documented to have a strong impact on biofilm structure^59^, and studies on stagnation generally involve cessation of or fluctuations in flow. The conditions in this study would therefore be representative of pipes that maintained the same flow rate of water but had increasing water age due to upstream conditions. From Phase I to Phase II, cell counts increased in both the bulk water and biofilm, but the accumulation rate of cells increased more in the bulk water than in the biofilm (Fig. 4). Because biofilm samples from Phase I were not included in metagenomic analysis, comparisons of biofilm microbial community composition with water age could not be made.

A significant difference in microbial communities of biofilm and bulk water has been reported in previous studies profiling microbiomes in chlorinated and unchlorinated, simulated and real-world drinking water systems^60–62^, accredited to the different ecological niches biofilm and bulk water provide^12^. However, in this study, bulk water and biofilm samples were similar, which has been observed in other studies of building plumbing and high water age^19^. Additionally, diversity of bulk and biofilm microbial communities were not statistically different by Shannon Index (Supplementary Fig. 16). Although biofilm detachment rates could not be quantified directly here, the combined net growth and transfer rates based on number balances were always high and increased with elevated water age (Fig. 4). Detachment rates of cells from biofilm have previously been found to be equivalent to the net growth rate^58^, meaning biofilm microbial communities can have a strong influence on bulk water communities. Here, the similarity between bulk and biofilm communities from the same reactor (Fig. 5, Supplementary Fig. 20, and Supplementary Fig. 21) indicated a high level of transfer between the bulk water and biofilm. We identified several MAGs (Mycobacterium_3, Novosphingobium_1, Novosphingobium_2, Methylobacterium_1) that were enriched in bulk water and several (Hyphomicrobium_3, Planctomycetes_1, Pirellulales_2, Nitrospira_1) that were more abundant in the biofilm. Previous studies have also reported that *Novosphingobium* spp. and *Methylobacterium* spp. were more abundant in bulk water than biofilms^63,64^. Interestingly, *Hyphomicrobium* spp. and *Nitrospira* spp. have been reported to be higher in stubborn biofilms that are more resistant to detachment^65^. Overall, the genera that we found to be enriched in the biofilm overlapped with those found in biofilms in previous drinking water studies in chloramine systems and on PVC^65–67^, but differed from free chlorine systems^63,68–70^.

### Influence of sampling point and historical contingency

**Fig. 9:**
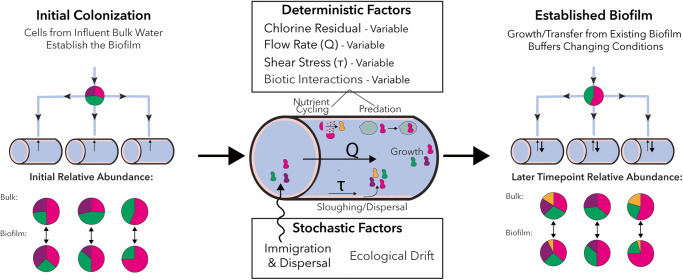
Conceptual schematic of microbial community assembly. Stochastic and deterministic factors lead to differential initial biofilm colonization in parallel sections of branching pipes. Over time, both deterministic and stochastic factors influence microbial communities. However, microorganisms that were already present in the biofilm buffer the community to these changes (i.e., historical contingency), resulting in communities more similar over time than conditions might suggest.

Plumbing microbial communities within buildings experiencing changing water age have previously been observed to have differences in communities across sampling locations but fairly consistent communities over time^19,29^. In our previous study of building plumbing, distinct microbial communities at drinking water fixtures within the same building stayed relatively consistent across wide fluctuations in water usage during stagnation and flushing^29^. This phenomenon could be partially due to biofilm that had been established at each location and continued to influence bulk water microbiota at later time points. In ecology, this principle is referred to as historical contingency, or the pattern of arrival of initial colonizing organisms, which has been documented to be important for microbial communities^71–73^. Supporting this model of drinking water microbial community assembly, in a prior study utilizing 14 replicate reactors, ecological drift (i.e., initial stochastic colonization) and subsequent biotic interactions created dramatically different communities across the reactors^74^. In this study, there was more overlap in microbial taxa between the reactors, and deterministic factors in assembly (i.e., operational differences in flow rate or differential wear on internal reactor elements), in addition to stochastic ones, likely contributed to the observed compositional differences. Additionally, in Phase I, chlorine concentrations and bacterial abundance were significantly different by reactor, and microbial communities clustered by reactor, pointing to initial deterministic differences. In Phase II, environmental conditions became more homogenous across the reactors as chlorine and cell counts were no longer significantly different; however, microbial communities continued to cluster by reactor, which supports that historical contingency played a role. Furthermore, throughout the experiment, the majority of bacterial cells present in the reactor bulk water derived from the reactor (i.e., growth in the bulk water or transfer from the biofilm) versus entering in the tap influent. In other words, unique microbial communities that developed in the reactors in phase I, either due to initial stochastic colonization from the tap reservoir or deterministic differences in environmental factors, were perpetuated over time and through changing conditions through growth and transfer from the biofilm (Fig. 9). Therefore, biofilm in a location has strong influence on bulk water microbiota and can buffer short-term environmental changes. However, sustained environmental changes can gradually modify communities in the biofilm and therefore bulk water. These findings could point to the importance of engineering drinking water pipe microbial communities in their early stages. A suggestion for future work is to incorporate ecological modeling and measurement of additional physicochemical parameters to investigate whether heterogeneity in reactor assembly can be predicted.

### Limitations and challenges in studying microbiota of drinking water distribution systems

Reactors provide an alternative to full-scale distribution systems that allow fine-scale control of hydraulics and sampling^72,75,76^. However, we found significant variability across reactors, pointing to the need for replication and suggesting that different points in distribution systems are likely also distinct (Section “Variability across the Five Replicate Reactors” and Supplementary Results Section 2). In addition, microbial communities and biofilms in reactors tend to be more homogenous and less diverse than those in real conditions, which is attributed to the relative continuity in environmental conditions^40^. Furthermore, hydraulics in annular reactors resemble the hydraulics of a continuously stirred tank reactor model, unlike real distribution systems. This has implications for the length of time a packet of bulk water is exposed to the same patch of biofilm and could lead to more sharing of organisms than in an actual system that more closely resembles a plug flow model. In this study, the reactors also represented a hybrid condition between DWDSs and building plumbing. Based on flow rate and water age alone, the reactors represented a pipe segment in a DWDS main with continuous flow where water age was increasing temporally. However, the high surface-area-to-volume ratio resembled building plumbing, and because the influent water to the reactors passed through a building plumbing system, the water quality and chemistry entering the reactors was more representative of building plumbing.

Bulk water microbiota in DWDSs are commonly monitored using culture-based methods, such as the measurements of total coliforms required in DWDSs in the United States^77^, but advanced methods (e.g., flow cytometry, DNA-based) are commonly applied in research contexts and are gaining popularity among water utilities. Cell counts via flow cytometry and ATP concentrations have been shown to be quick, effective, and comparable measures of microbial abundance^36^, and ICC and ATPi trended together throughout the experiment. When conducting community analysis, metagenomic sequencing results can be analyzed using read-based approaches, single copy genes, or MAGs. Although 28% of reads did not map to dereplicated bins, the MAGs showed similar trends and taxonomy to reads and single copy genes. For functional targets, metagenomics provided more breadth and a higher detection than qPCR, which contradicts the widely held belief that qPCR is a more sensitive and reproducible method for detection of rare populations^12^. In addition to functional genes, metagenomics can enable detection of eukaryotes and viruses; however, more work is needed to improve databases and analysis tools for these targets.

Few studies have compared multiple methods for quantifying bacteria in drinking water biofilms. Standard analysis procedures are lacking for microscopy of water biofilms that would enable quantitative analysis, and there are many potential pitfalls to consider when designing a protocol^78^. Flow cytometric cell counts and ATP measurements enable aggregate quantitative measurements of homogenized biofilm^79^, although there is always a risk that biofilms are not fully homogenized or that eDNA in the biofilm matrix is counted as a cell. Homogenization approaches should be standardized to ensure targets are properly released from the EPS matrix for detection. Fluorescent confocal laser scanning microscopy coupled with quantitative image analysis could provide an alternative method for calculating biofilm biomass while also obtaining information about biofilm structure and distribution, although it is more time and labor intensive than measuring cell counts and ATP. Prior studies have employed this technique and set the groundwork for a future consensus protocol but have each faced their own limitations, such as fixing and drying of biofilms which may impact their structure and volume^41^. In this study, there were some discrepancies between microscopy and ICC/ATP. Methods development and standardization for microscopy of drinking water biofilms on various substrates are needed. For drinking water biofilms on plastic substrates, we recommend building upon the protocols presented herein that involve microscopy of hydrated, unfixed biofilms but with further optimization of dye concentration to ensure penetration into thicker biofilms or with more advanced quantitative image analysis that accounts for all area within a cluster.

### Challenges in measuring and managing water age

Water age, defined here as the total residence time between water treatment and consumption, is impossible to measure directly but can be estimated through the use of hydraulic models^80^. A limitation in this study is that modeled estimates of water age were not available, forcing reliance on relative changes in building occupancy as well as building water meter readings. Building water usage could be a good proxy for water age if the majority of the residence time of water were spent in the building, but it does not encapsulate time spent upstream of the building. We suspect that, due to shelter-in-place, water demand in the surrounding area decreased in Phase II. For this reason, it is unsurprising that water quality parameters (e.g., chlorine, ICC) did not correlate with monthly building water usage (Supplementary Fig. 9). Another possible contributing factor to the discrepancy in water demand and cell counts at each time point is that the return of microbial abundance and community composition to baseline levels may be a gradual process, as demonstrated by a recent study on building reopening^35^.

In full-scale systems, elevated water age is primarily a concern for microbial water quality in distribution systems with prior detection of opportunistic pathogens and with a high potential for regrowth in distribution (i.e., cell counts far below the carrying capacity of water). Although water age is highest at the distal points of DWDS within building plumbing, evidence is emerging from multiple studies that flushing building plumbing likely is not a feasible solution for continued prevention of regrowth when water demand is low^29,31^. Instead, utilities could increase disinfectant residuals in network sections with elevated water age through the use of chlorine booster stations^80^. When water age issues are suspected, water quality monitoring should be conducted across multiple time points and for time periods that extend beyond any alterations in water demand. Although resource-intensive, incorporating sensors and modeling into DWDS to detect changes in flow and monitor water age in real time could assist in predicting where and when water quality issues may arise due to changes in water demand^81,82^.

## Methods

### Source water and operation of rotating annular reactors

Five biofilm annular reactors (Model 1320 LS; Biosurface Technologies Corp., Bozeman, Montana) were operated for 13 months, from September 30, 2019 to October 21, 2020. Annular reactors have been commonly used as bench-scale models to mimic water distribution systems and to simulate biofilm growth^4,12,83,84^. The annular reactors comprised rotating inner cylinders that subjected biofilm growing on removable, flush-mounted coupons to shear forces typical of water systems, independent of flow-controlled residence time. The removable coupons used in this experiment were gray PVC slides; PVC pipes represent about 10% of the local drinking water distribution system [G. Williams, personal communication, 2020]. Inner cylinder rotation was set to 50 rpm which translated to shear forces of 0.25 N/m^2^ ^85^. Reactors were supplied with tap water from a continuously flowing laboratory fixture (cold water) that was set to 150 mL/min (Supplementary Fig. 1). The laboratory was on the third floor of a four-story building constructed in 1959 on the University of California, Berkeley campus. The tap was connected to a 10 L reservoir using autoclaved silicone tubing, and water was peristaltically pumped from the reservoir into the reactors at an average rate of 2.1 mL/minute. This resulted in mean hydraulic residence times (HRT) of approximately 1 h in the storage reservoir and 8 h in the reactors. The study building was served by a drinking water treatment facility using protected surface water treated by primary disinfection, coagulation, and filtration, followed by applying chloramine as the secondary disinfectant^86^.

As part of COVID-19 precautions, academic buildings on the University of California, Berkeley campus were closed to all but essential activities on March 10, 2020 (day 162 of the experiment), followed by the San Francisco Bay Area shelter-in-place order on March 17, 2020. The drinking water utility reported no differences in chlorine residuals or water age in the water mains supplying campus [G. Williams, personal communication, 2020]. The lower building water usage during shelter-in-place led to a higher water age of the input water to the reactors. Based on water meter data, water usage in the months following building closure dropped to an average of 50% of typical levels in the building where the annular reactors were operated (Supplementary Fig. 2). The building reopened at reduced occupancy midway through Phase II on June 30, 2020. The mean HRT in the annular reactors was consistent throughout the study period, such that the study isolated the effect of the naturally elevated water age in the influent tap water, due to decreased building water usage. The experiment consisted of two phases (Fig. 1): (I) Influent with typical water age for 6 months to set a baseline and allow biofilms to develop; (II) Influent with elevated water age for 7 months.

### Bulk water and biofilm sample collection

Water samples were collected from the tap, tap reservoir, reactor bulk, and reactor biofilm (Supplementary Fig. 1). Total chlorine and intact cell counts of both the tap reservoir feeding the reactors and the tap directly were collected for a subset of sampling events (21 total tap samples; Supplementary Table 1) and were not statistically different (Wilcoxon, *p* > 0.05; Supplementary Fig. 3). Therefore, the short residence time in the tap reservoir did not significantly influence water quality. Measurements of samples collected from the tap reservoir but not the tap are therefore reported throughout. Sample names include sample type and reactor number (tap reservoir: tapres; reactor bulk water: AR1, AR2, AR3, AR4, AR5; biofilm: BF1, BF2, BF3, BF4, BF5) followed by the day of the sampling event.

For bulk water physicochemical measurements, flow cytometric cell counts, and ATP measurements (described further in Sections “Physicochemical water quality measurements”, “Flow Cytometry” and “ATP”), reactor bulk water samples (*n* = 190; Supplementary Table 1) were collected via the top sampling port of each reactor without stopping the rotation of the inner cylinder. Samples from the 10 L reservoir preceding the reactors and directly from the tap were also collected. Samples were aliquoted immediately. Physicochemical parameters (chlorine, temperature, and pH) were measured immediately. The aliquot for microbial analysis was dosed with sodium thiosulfate to quench the chloramine residual and then stored at 4 °C until analysis within 24 h.

Nucleic acids-based analysis was performed during a subset of sampling days that occurred toward the end of each phase. For nucleic acids-based analyses of bulk water, reactor effluent water was collected over a 24-h period into a sterile container on ice during four sampling events (Supplementary Table 1). Collected water was then filtered through a 0.2 μm polyethersulfone filter (Millipore Sterivex) using a peristaltic pump, and filters were stored in sterile 50 mL tubes at -80°C until extraction. Tap reservoir water was pumped directly from the reservoir. The volume of water filtered was 3 L for reactor effluent and 10 L for tap reservoir, or until clogging occurred.

For biofilm flow cytometric cell counts, ATP measurements, microscopy, and nucleic acids-based analyses, reactor slides were removed through the top sampling port with sterilized tweezers after gradually slowing reactor rotation to 0 rpm. Biofilm samples were collected after (but within 72 h of) bulk water sample collection so that any biofilm detachment upon changes in rotation would not affect bulk water microbial abundance measurements. After slide removal, slides were immediately cut into two pieces (5 cm and 10 cm) with a sterilized pipe cutter. After sample collection, a sterile PVC slide was inserted to replace the one that was removed so that empty slots did not interfere with reactor hydraulics, and the reactor rotation was restarted gradually. The 5 cm segment of the sampled slide was used for microscopy (Section “Fluorescent Confocal Laser Scanning Microscopy of Biofilm”), while the 10 cm segment was homogenized for downstream flow cytometry, ATP measurements, and nucleic acids-based analyses. To homogenize biofilm, the cut PVC slide was placed into a 50 mL centrifuge tube containing 50 mL of 0.1 μm-filtered and dechlorinated (using sodium thiosulfate) tap water and then sonicated for 3 min using a Bransonic 3510 sonicator. The water was collected, and slides were removed with flame-sterilized tweezers then scraped with a sterile cell scraper (Research Products International). The slide was rinsed with 10 mL of sterile tap water, and the rinse water was collected. Last, the scraper was sonicated for 1 min in 5 mL of sterile tap water, and the water was collected. All the collected water (~65 mL total) was combined and used for downstream analysis. For a subset of sampling events (*n* = 7; Supplementary Table 1), 45 mL of homogenized biofilm was filtered through a 0.2 μm polycarbonate flat membrane filter using syringe filtration. Filters were rolled, placed into 2 mL tubes, and stored at -80°C until extraction.

### Physicochemical water quality measurements

All bulk water samples were analyzed for temperature, pH, total chlorine, and free chlorine. Total chlorine and free chlorine were measured onsite with DPD Hach methods 8167 and 8021, respectively, both with a limit of detection (LoD) of 0.02 mg/L as Cl_2_.

### Flow cytometry

Total and intact cell concentrations of bulk water and homogenized biofilm were measured following methods previously described^36,87^ using an Accuri™ C6 flow cytometer (BD Biosciences). Drinking water biofilms have been previously analyzed with flow cytometry, although no standardized method exists^79^. In brief, triplicate 500 µL sample aliquots were stained with SYBR Green I (Sigma Aldrich) to identify total cells (TCC) or were stained with both SYBR Green I and propidium iodide (Life Technologies) to distinguish cells with intact membranes (ICC). Measurements were performed at the “fast” flow rate of 66 µL per minute on sample volumes of 50 µL with an excitation wavelength of 488 nm and threshold of 800 on the green fluorescence channel. Microbial cell signals were distinguished and enumerated from background and instrument noise on density plots of green (FL1; 533 ± 30 nm) and red (FL3; >670 nm) fluorescence using gate templates previously developed for water samples^88^. The lower limits of quantification were determined previously on the same instrument for intact cell count (22 cells/mL) and total cell count (12 cells/mL)^87^. For a negative control, 0.22 μm filtered, Millipore Milli-Q water was used. Some samples were diluted up to 5x with filtered (Medical Millex-VV Syringe Filter, 0.1 μm, PVDF), dechlorinated tap water to stay within the instrument quantification limits.

### ATP

Total ATP (ATPt) and extracellular ATP (ATPe) were determined using the BacTiter-Glo™ reagent (Promega Corporation) and a Promega Glomax 20/20 luminometer as described elsewhere^89–91^. Briefly, samples (500 μL) and ATP reagent (50 μL) were warmed to 38°C simultaneously in separate sterile Eppendorf tubes then combined, incubated at 38 °C for an additional 20 s, and measured. Luminescence data were collected as relative light units (RLU) and converted to ATP (nM) by means of a calibration curve made with a known ATP standard (10 uM; Promega) diluted in ATP-free, sterile water (autoclaved and 0.1 μm filtered dechlorinated tap water). For extracellular ATP analysis, each sample was filtered through a 0.1 μm sterile syringe filter (Millex®-GP, Millipore) followed by analysis as described above. The intracellular and cell-bound ATP (ATPi) was calculated by subtracting the extracellular ATP from the total ATP for each individual sample. ATP was measured in triplicate for all samples.

### Fluorescent confocal laser scanning microscopy of biofilm

Following biofilm sampling procedures described in Section “Bulk Water and Biofilm Sample Collection”, 5 cm portions of PVC slides from the reactors were glued to plastic petri dishes to prevent movement during measurements. Biofilm on the slide was then stained with 50 µL of Invitrogen SYTO™ 9 Green Fluorescent Nucleic Acid Stain (SYTO9), Invitrogen Sypro Orange Protein Gel Stain (Sypro Orange), and Invitrogen Concanavalin A Alexa Fluor® 647 conjugate (ConA), in that order so that protein dyes did not bind the lectins^41^. Dyes were diluted to final concentrations in TC buffer (SYTO9: 20 µM, Sypro Orange: 10x, ConA: 200 µg/mL), stored at −20 °C, and used within 1 week. Original stocks of Concanavalin A were resuspended to 5000 ug/mL in sodium bicarbonate solution. Each dye was incubated on the biofilm for 10 to 20 min and then rinsed with dechlorinated, filtered tap water. Biofilm slides were transported to the microscopy facility while protected from light during the final incubation period (Concanavalin A). Slides were then gently resuspended in dechlorinated, filtered (0.22 µm) tap water from the same fixture that was feeding the reactors.

Fluorescence confocal laser scanning microscopy (CLSM) was performed on a Zeiss 710 using a 40x water immersion dipping lens to visualize biofilms without disturbing their structure (hydrated and without a fixation step). Bidirectional scans were performed with averaging of 1, pinhole of 1 AU, depth of 8 bit, speed of 9, and gains of 624, 755, and 816 for SYTO9, Sypro Orange, and ConA, respectively. The 633 nm laser was used for ConA, and the 488 nm laser was used for SYTO9 and Sypro Orange. The collection wavelength spectra of the three channels were optimized to limit bleedover (SYTO9: 500–530 nm, Sypro Orange: 560–620 nm, ConA: 660–750 nm). Frames were taken in the z direction at an interval of 1 µm with scan start and stop points selected manually for each image based on the apparent start of the substrate to end of continuous biofilm. Each frame was 1024 × 1024 pixels representing an area of 212.5 × 212.5 µm^2^. At least five representative z-stacks of each slide were captured at random within the stained area, representing a total area of at least 2.25 × 10^5^ µm^2^ based on recommendations that representative biofilm samples capture a minimum total area of 1 × 10^5^ µm^2^ ^92^. Six replicates of each of the following controls were imaged: sterilized slides with no stain, sterilized slides stained only with SYTO9, sterilized slides stained only with Sypro Orange, sterilized slides stained only with ConA, and sterilized slides stained with all three dyes (Supplementary Fig. 4).

### DNA concentration and extraction

DNA collection and extraction followed methods previously described^93^ using the PowerWater kit (Qiagen). Briefly, biomass from water samples (1.5 L) was filter-concentrated on the day of sampling using peristaltic pumps, autoclaved silicone tubing, and in-line 0.2 µm PES filters (Millipore Sterivex). Filters were stored at −80 °C until extraction. DNA extraction was conducted using a modified DNeasy PowerWater kit (Qiagen) protocol which involves physical, chemical, and enzymatic lysis steps^92,93^. Filters were cut with a scalpel, inserted into tubes with lysozyme (Thermo Fisher) and Tris EDTA, and incubated shaking at 37 °C for 60 min. Next, kit reagent PW1 (Qiagen) and Proteinase K (Life Technologies) were added followed by incubation with shaking at 56 °C for 30 min. Chloroform/isoamyl alcohol (Acros Organics) was added, and then samples underwent bead beating for 40 s in lysing matrix E tubes (Thomas Scientific). After centrifugation, the aqueous phase was transferred to new tubes, and the PowerWater kit was used following manufacturer protocols. After extraction, DNA was quantified in every sample using the Qubit dsDNA HS assay (Life Technologies) and then stored at −80 °C until downstream analyses.

An extraction negative control was included in every extraction batch for quality control and consisted of an unused filter (Millipore Sterivex) that was processed through extraction steps alongside samples. In addition, manifold negative controls for bulk water processing consisted of 3 L of 0.2 µm filtered, autoclaved MilliQ water that was filtered and processed according to the same protocol as samples. Slide negative controls for biofilm processing consisted of sterilized PVC slides that were processed according to the same protocol as samples. Dilutions of a commercially available mock community (ZymoBIOMICS Microbial Community Standard; cat. 6300) were extracted as positive controls for metagenomics (Supplementary Table 2).

### qPCR

Quantitative PCR (qPCR) was performed on samples concurrently and using the same methods as previously described^29^. Briefly, 34 samples were processed via qPCR on a QuantStudio3 Real-Time PCR System qPCR machine (Thermo Fisher) for four targets of interest: the bacterial ammonia monooxygenase gene of ammonia-oxidizing bacteria (*amoA*)^94^ the macrophage infectivity potentiator gene of *L. pneumophila* (*mip*)^95^, polymorphic regions of ITS sequences of *Mycobacterium avium* complex (MACF/MACR)^96^, and the oprL gene of *Pseudomonas aeruginosa* (*oprL*)^97^. Reactions consisted of 1 μL template in a 10 μL reaction, except for *amoA* in which 5 μL of template was included in a 20 μL reaction, with detailed reaction mixes reported in Supplementary Table 3. Cycling conditions followed master mix manufacturer recommendations but were optimized for individual laboratory conditions (Supplementary Table 4). Each plate contained no-template controls (PCR water) and a standard curve consisting of synthetic DNA (gBlock, IDT) of known concentrations spanning 5 to 10^5^ gene copies (gc) per reaction. All samples, standards, and controls were analyzed in technical triplicate. Standard curve efficiencies ranged from 89.8% to 114.6% with R^2^ of 0.984 to 0.998 (Supplementary Table 5). None of the no-template controls (NTCs) amplified throughout the study.

To determine the limit of detection (LoD) and limit of quantification (LoQ), 12 replicates of standard material were run across at least three plates (Supplementary Table 6). The LoD was defined as the interpolated gene copy number where 95% of standard technical replicates amplified, and the LoQ was defined as the interpolated gene copy number where the coefficient of variation of standard technical replicates was 35%^98^. Inhibition was assessed by spiking a subset of DNA extract with standard materials and measuring the target at four dilutions. Any dilution with a measured Cq that deviated more than 1 Cq from expected was considered inhibited. Only one sample showed inhibition at any dilution (*oprL* assay, AR4_372), which was diluted out at 4x (Supplementary Table 7). Because of such minimal signs of inhibition, samples were processed without dilution.

### Sequencing and metagenomics

Thirty-four samples were sequenced as well as two positive controls from mock communities, one negative control for bulk water processing, and one negative control for biofilm processing. Controls are further described in Section “DNA Concentration and Extraction”.

Library preparation for metagenomic sequencing was performed at the Functional Genomics Laboratory at UC Berkeley. A bioanalyzer run was used to confirm extraction quality and concentration prior to sequencing. Briefly, 500 bp insert libraries were constructed using the Roche KAPA hyper prep kit with a PCR amplification step. Sequencing was performed on one lane of an Illumina NovaSeq S4 flow cell with 150 bp paired-end reads at the Vincent J. Coates Genomics Sequencing Laboratory at UC Berkeley, such that each sample was sequenced to an average depth of 75 million reads (Supplementary Table 8).

Illumina adapters and PhiX were removed from raw reads using bbmap v38.79^99^, and sequences were quality trimmed using sickle (https://github.com/najoshi/sickle). The performance of the quality control and trimming was visually inspected using fastQC^100^. Pairwise MASH distances were calculated for all samples using mash v2.2^101^. These read-based distances (Supplementary Fig. 5) between samples were used to determine which samples to coassemble and crossmap to assist with binning. Reads from each sample were either individually assembled or coassembled using IDBA-UD with a pre-correction step to normalize uneven sequencing depths^102^. Samples included in each coassembly are listed in Supplementary Table 9.

Reads were mapped to the assemblies to which they contributed for coassembled samples and mapped to closely related assemblies for individually assembled samples using bowtie2 v2.3.5^103^ with default parameters. Samtools (http://www.htslib.org/) and Anvi’o v7.1^103^ were used to profile the resulting bam files for each assembly. Profiles for individual samples were merged into coassembly profiles using anvi-merge with enforced hierarchical clustering.

Taxonomic analysis of contigs was performed with Kaiju^104^ using the nr_euk database version r2021-02-24. Single copy genes and rRNAs were identified within Anvi’o v7.1^105^ using hmmscan^106^. Single copy gene analysis was performed using ribosomal protein S2 (RPS2), the single copy gene with the highest count across all assemblies. Functional annotation was performed using hmmscan with KEGG KOFAM profiles in Anvi’o (KOFAM HMMs from December 23, 2020). Hidden Markov models were used to identify single-copy genes in contigs (function anvi-run-hmms). The function anvi-estimate-scg-taxonomy was used to investigate taxonomy and estimate the number of genomes as an input for automated binning with Concoct (see Section “Metagenome-assembled genome (MAG) analysis”)^106^. GTDB-Tk^107^ was used to find the taxonomy of the bin (ARSTAG_ARBF_2_post_bin_29_1) with unknown taxonomy up to the domain level output (Supplementary Results Section 1). Potential eukaryotic bins were identified using the Domain suggested by Anvi’o using SCGs as well as bin GC content and size. Using anvi-get-sequences-for-hmm-hits within Anvi’o, 18 S ribosomal RNA gene sequences were extracted and (i) a search was conducted using BLASTN against NCBI-nr with default parameters and (ii) sequences were aligned to the SILVA database^108^ using SINA^109^. Anvi’o was used to reconstruct metabolism for modules in the KEGG MODULE database^110^. The Benchling alignment tool was used to compare qPCR primers to metagenomic gene calls.

Binning of the scaffolds was initially performed using CONCOCT, with manual refinement in Anvi’o. CONCOCT was run with the -c parameter to produce a number of bins equal to the number of unique single copy genes identified by anvi-estimate-scg-taxonomy, except in the case that zero genomes were predicted (negative control) in which case the input of one was used. Bins were then refined manually by opening each bin generated by CONCOCT individually with anvi-refine in “collections” mode. Bins were curated based on hierarchical clustering, completeness, redundancy, coverage, GC content, and taxonomy. Some bins were merged using anvi-merge-bins and then further refined, and bins were summarized iteratively using anvi-summarize. Manual curation resulted in 345 bins. To ensure quality, bins with completeness <= 90% and redundancy >= 10% were dropped from the final collection. Genomes were dereplicated using dRep^111^ with the CheckM lineage workflow and the default percent identity cut-off of 99% average nucleotide identity, resulting in 90 dereplicated bins. Reads were then mapped to dereplicated genomes using bowtie2 as above. Bins derived from controls that did not appear in samples were removed for community analysis, resulting in 83 bins. Detection was defined as at least 1x coverage over at least 50% of the genome.

Negative controls for bulk water and biofilm processing were sequenced. Qubit values for the manifold control and biofilm control were 0.04 ng/µL and below the limit of detection (≤0.01 ng/µL), respectively. However, after sequencing, these controls yielded 309,000 and 43 million reads, suggesting contamination of the biofilm control likely occurred during library preparation. Based on the taxonomy of reads in the biofilm control, it seems likely that the contamination was from higher biomass samples rather than external contamination. Significant cross-contamination of low biomass controls is common and can be harder to remove in silico than external contamination^112^. Based on differences in community composition compared to other reactor samples from the same day, cross-contamination of the sample from AR4 bulk water on sampling day 162 (0.21 ng/µL) likely occurred. The microbial community in this single Phase I sample clustered with Phase II samples despite no difference in water quality and cell count data compared to other Phase I reactor samples. Therefore, this sample was removed for most metagenomic analyses.

### Data analysis

Data analysis was conducted in R (v4.1.3) and Python (v3.9.12). Plots were generated using ggplot2 (v3.3.6).

Coefficients of variation (CV) were calculated as the arithmetic standard deviation divided by the mean, while geometric coefficients of variation (gCV) were calculated as the geometric standard deviation minus one. As water quality data were non-normal (Shapiro–Wilk’s, R function, *p* < 0.001), hypothesis testing was conducted using Kruskal–Wallis (R function kruskal.test) and the non-parametric pairwise Wilcoxon rank sum test (“Mann–Whitney,” R function wilcox.test) with a significance threshold of 0.05, and correlations were computed using rank-based Kendall’s tau-b (R function cor) to account for tied values.

### Quantitative image analysis for biofilm characterization

An in-house Python pipeline using Java (v8), bioformats (0.0.0) and scikit-image (0.19.3) was used to enable customized calculations and normalizations. CLSM image files (.czi) were imported into Python using python-bioformats. Images were denoised through median filtering and then transformed into binary arrays using Otsu thresholding (scikitimage, threshold_otsu). The first four z slices were then removed from all images because area coverage greater than 1% was measured in the majority of stained controls in this range (Supplementary Fig. 4), likely because the dyes attached to the plastic when resulting concentrations were high because there was no biofilm to which dyes could bind. The PVC did not autofluoresce when no dyes were added. After merging all three channels, filtering, thresholding, and removing base stacks, maximum area was calculated as the area in the image with the maximum number of detected pixels, and maximum height was calculated as the number of stacks with at least one detected pixel. Area of extracellular polymeric substances (EPS) was calculated similarly but only after merging the two channels associated with Sypro Orange and Concanavalin A. Biovolume was calculated by summing the area of each slice and multiplying by the height of each slice (1 μm).

### Number balances on intact cell counts

Number balances were performed on intact cell counts in the reactor bulk water. In Eq. 1, a generic number balance equation is presented for cells in a reactor. $\frac{{dN}}{{dt}}$ is the total accumulation rate in the reactor (cells/day), while $\left( {\frac{{dN}}{{dt}}} \right)_{bulk}$ and $\left( {\frac{{dN}}{{dt}}} \right)_{biofilm}$ are accumulation rates in the bulk water and biofilm, respectively. Because there were no direct measurements for growth/decay rates of cells in the bulk water or rates of transfer of cells from the biofilm to the bulk water, these rates could not be separated and were instead grouped into a single term: the Net Growth/Transfer Rate (NGTR).

The first ICC number balance was performed according to Eq. 2, where the system was modeled as a continuously stirred tank reactor with accumulation in the bulk water and biofilm (Supplementary Fig. 6). Accumulation was calculated as the difference in total cells in the reactor (bulk + biofilm) between *t*_1_ and *t*_2_, where *t*_1_ and *t*_2_ are two consecutive time points where all necessary parameters were measured. Because biofilm ICC was only measured at seven timepoints, the resulting number balance only produced six data points for these time periods. For the cell counts in the influent $C_{tap_{avg}} \ast Q$ and effluent terms $C_{AR_{avg}} \ast Q$ from advective flow, average values from all sampling events collected between *t*_1_ and *t*_2_ were used, to better account for variability in these measures and irregular time intervals.

A second number balance was performed according to Eq. 3, where the system was modeled as a continuously stirred tank reactor at an instantaneous steady state. By assuming there was no accumulation of cells in the reactor during the time frame of one sampling event, the number balance did not rely on biofilm ICC measurements, which resulted in more output data points (*n* = 170) than with Eq. 2 (*n* = 30). In addition, this number balance is not impacted by the variable time intervals between biofilm sampling events.

Generic Reactor Number Balance (Eq. 1)

1 $$\frac{{dN}}{{dt}} = \left( {\frac{{dN}}{{dt}}} \right)_{bulk} + \left( {\frac{{dN}}{{dt}}} \right)_{biofilm} = tap_{IN} - AR_{OUT} + NGTR$$

Non-steady State Number Balance (Eq. 2)

2 $$\frac{{dN}}{{dt}} = \frac{{\left( {C_{AR} \ast V + C_{BF} \ast A} \right)_{t_1}^{t_2}}}{{dt}} = C_{tap_{avg}} \ast Q - C_{AR_{avg}}\ast Q + NGTR$$

Instantaneous Steady State Number Balance (Eq. 3)

3 $$0 = C_{tap} \ast Q - C_{AR} \ast Q + NGTR$$

### Metagenomic data analysis

Non-parametric, permutational ANOVA (PERMANOVA) of Aitchison distance was used to test differences in community composition using the adonis2 function in vegan (v2.6-4)^112^. Aitchison distance accounts for the compositional nature of relative abundance data and was calculated using Euclidean distance after a clr transformation. Differential gene abundance analysis was performed using DESeq2 (v1.34)^113^. Nonmetric multidimensional scaling (NMDS) ordinations were conducted using Bray–Curtis distances for MAGs or MASH distances for reads. Redundancy analysis (RDA) ordination was performed using Hellinger distances of community composition data constrained around sample type and phase.

### Supplementary information

Supplemental Material

## Data Availability

The datasets generated and analyzed during the current study are publicly available at https://github.com/hannahgreenwald/Reactor-Water-Age. Sequencing data were deposited in NCBI SRA (BioProject PRJNA850455).

## Code availability

The underlying code for this study is publicly available at https://github.com/hannahgreenwald/Reactor-Water-Age.
