# Supplementary material for: Responses of drinking water bulk and biofilm microbiota to elevated water age in bench-scale simulated distribution systems
Source: NPJ Biofilms Microbiomes. 2024 Jan 22;10:7. doi: 10.1038/s41522-023-00473-6 (PMC10803812; doi:10.1038/s41522-023-00473-6)
Supplement: Supplementary file 1 — Supplemental Material [file 41522_2023_473_MOESM1_ESM.pdf]

Supplementary Information

Responses of Drinking Water Bulk and Biofilm Microbiota to Elevated Water Age in Bench-Scale Simulated Distribution Systems

Authors: Hannah Greenwald Healy[1], Aliya Ehde[2], Alma Bartholow[1], Rose Kantor[1]*, Kara L. Nelson[1]*

1. Department of Civil and Environmental Engineering, University of California, Berkeley, Berkeley, CA, USA.
2. Division of Environmental and Ecological Engineering, Purdue University, West Lafayette, IN, USA.

*Co-corresponding authors

Contents

SUPPLEMENTARY RESULTS 2
SUPPLEMENTARY FIGURES 3
SUPPLEMENTARY TABLES 18

Supplementary Results

Section 1. Identification of bin with unknown taxonomy

The bin (ARSTAG_ARBF_2_post_bin_29_1) was identified as d__Bacteria; p__Acidobacteriota; c__Acidobacteriae; o__Bryobacterales; f__Bryobacteraceae; g__; s__). The output showing that it belongs to the family Bryobacteraceae largely aligns with the NCBI nBlast results of the 23S gene, acquired through anvi-get-sequences-for-hmm-hits, showing that the top hit was Bryobacterales (Acc#: AP025252.1), albeit with only 88% identity.

Section 2. Variability of bulk water and biofilm parameters across annular reactors

Mean arithmetic coefficient of variation for total chlorine across the five reactors on each sampling day was 48.9%. Mean geometric coefficient of variation for ICC across the five reactors on each sampling day was 89.9%. For biofilm samples, ICC was not statistically different by reactor (Kruskal-Wallis, $p=0.90$).

On the final biofilm sampling day, three slides were sampled from reactor 3 and independently analyzed to compare variability at different scales of measurement. At the smallest scale, the average coefficient of variation of biofilm height in analyzing different image fields of the same biofilm slide was 74%, representing a high level of small-scale heterogeneity. Across the three slide replicates from the same reactor, the coefficient of variation of mean heights was lower, at 19.8%. Across the five reactors, the coefficient of variation in the means of each reactor height measured on the same day was 50%. A similar analysis was conducted for the same reactor 3 biofilm samples from day 387 with ICC. The mean geometric coefficient of variation of flow cytometry technical replicates was 106%, while the geometric coefficient of variation in ICC across the three slide replicates was 80.5%. The coefficient of variation in the means of biofilm ICC measured on the same day across the five reactors was only 23.2%.

Section 3. Quantification of nitrifying genes

One functional gene target was measured via qPCR: the nitrifying gene *amoA*. While multiple detected MAGs were identified as nitrifiers, out of 34 samples, only one sample (AR2_134) had a quantifiable concentration of *amoA* of 87.7 gc/L, while the theoretical limit of detection and quantification for a reactor sample was 60 gc/L. Using metagenomic data, the concentration of *pmoA*/*amoA* in this sample was approximated as 45.2 gc/L by summing the relative abundance of bins containing *pmoA*/*amoA* (assuming one copy per bin) and multiplying by TCC. This approximation is close to the concentration measured via qPCR; however, estimates based on metagenomic data from ten other bulk water samples exceeded *pmoA*/*amoA* estimates from AR2_134, with some concentrations as high as 1150 gc/L. Six genes were identified as *amoA*/*pmoA*. Of these, the *amoA* qPCR assay primers matched to two of them (identified as >6 matching bases and <3 mismatches) although none matched fully without mismatches, which may further account for low detection via qPCR with this *amoA* assay. Estimates based on metagenomic data were likely higher because of the inclusion of *pmoA* along with *amoA* and because estimated concentrations based on metagenomic data and flow cytometry may not be as susceptible to extraction losses and sample-specific inhibition as qPCR. Given that we did not distinguish between *amoA* and *pmoA* in metagenomic data, it is not surprising that *amoA*-specific qPCR produced only one detection even though the genes were identified in multiple samples via metagenomics.

Supplementary Figures

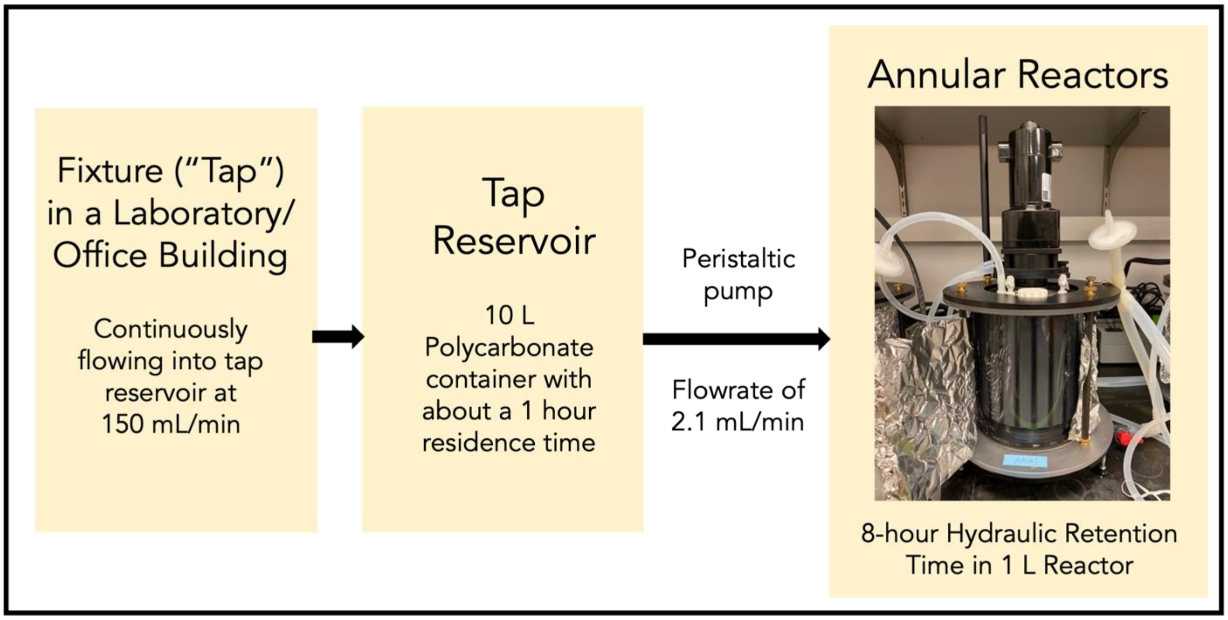

Supplementary Figure 1: Experiment Schematic. Schematic diagram of the experimental set-up with flow rates and hydraulic retention times. Water flowed from the tap to the tap reservoir and then was pumped into each annular reactor using a multi-channel peristaltic pump.

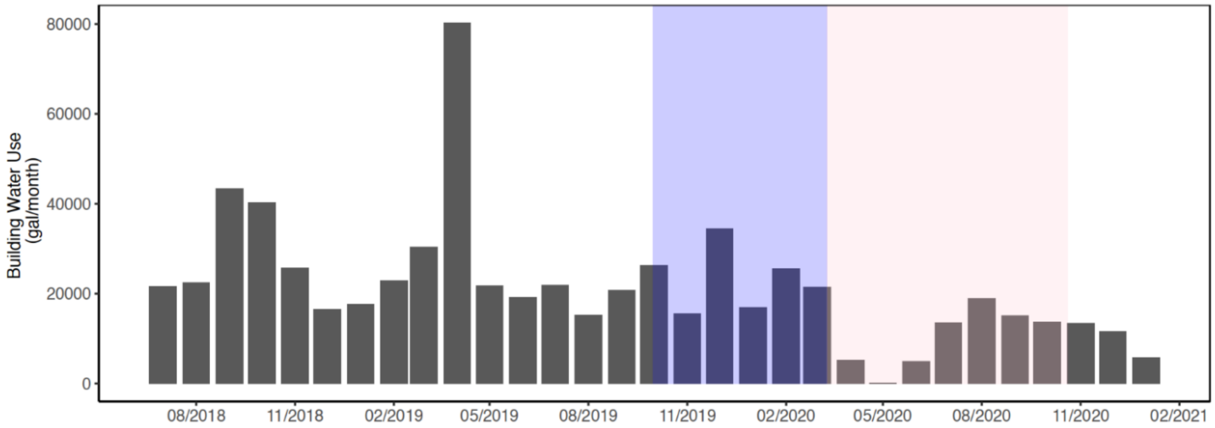

Supplementary Figure 2: Building Water Usage Over Time. Water usage in building feeding the reactors based on water meter readings. The water meter represents water usage in both the study building and the adjoining building. Purple represents Phase I of reactor operation, and pink represents Phase II.

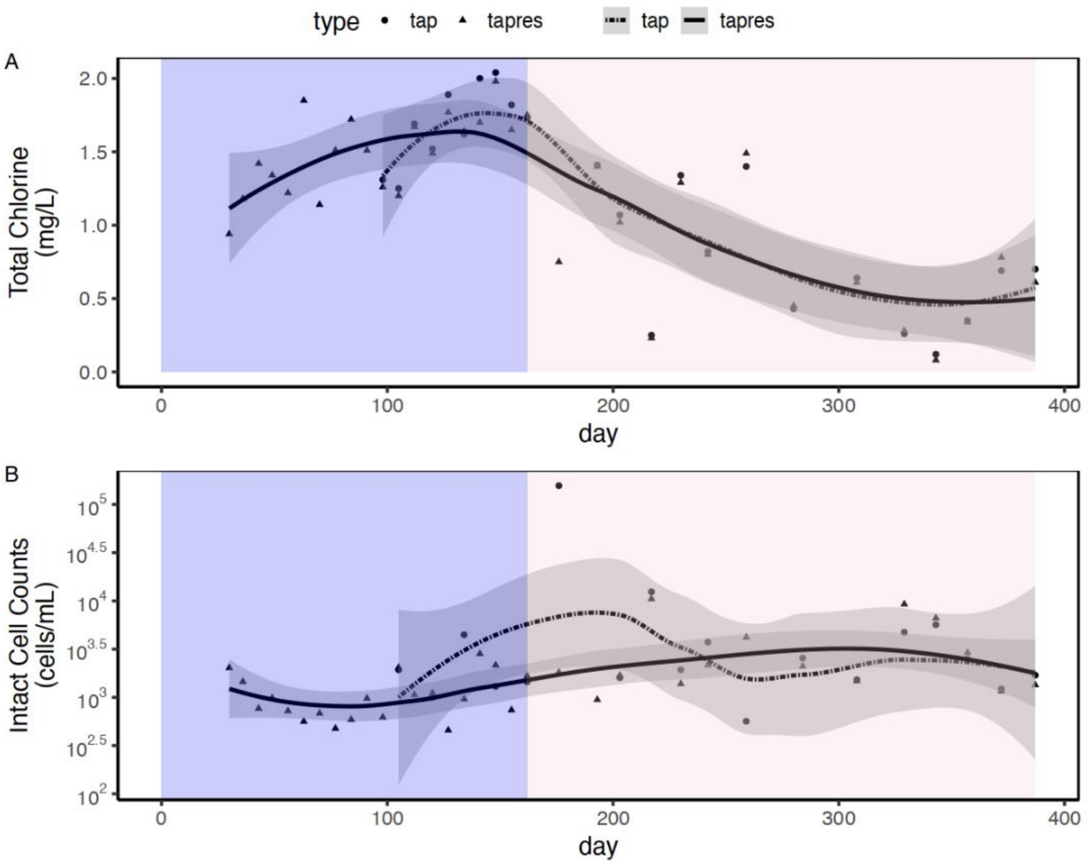

Supplementary Figure 3: Time Series of Tap and Tap Reservoir Samples. Comparison of total chlorine and intact cell counts of samples from both the tap and tap reservoir for a subset of sample dates.

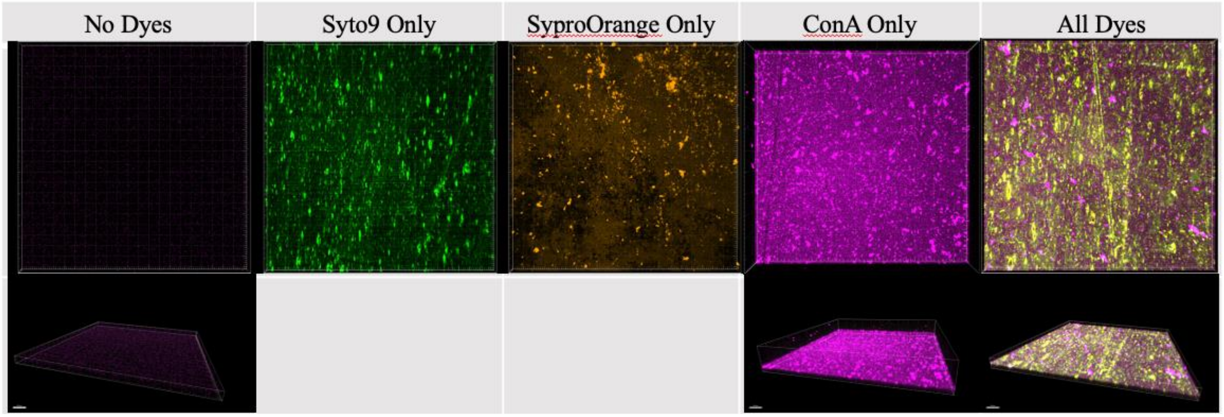

Supplementary Figure 4: Control CLSM Images by Fluorescent Dyes. Images of blank PVC slides that served as CLSM controls. Slides were imaged after (from left to right) no staining, staining with only SYTO9, staining with only Sypro Orange, staining with only ConA, and staining with all three dyes.

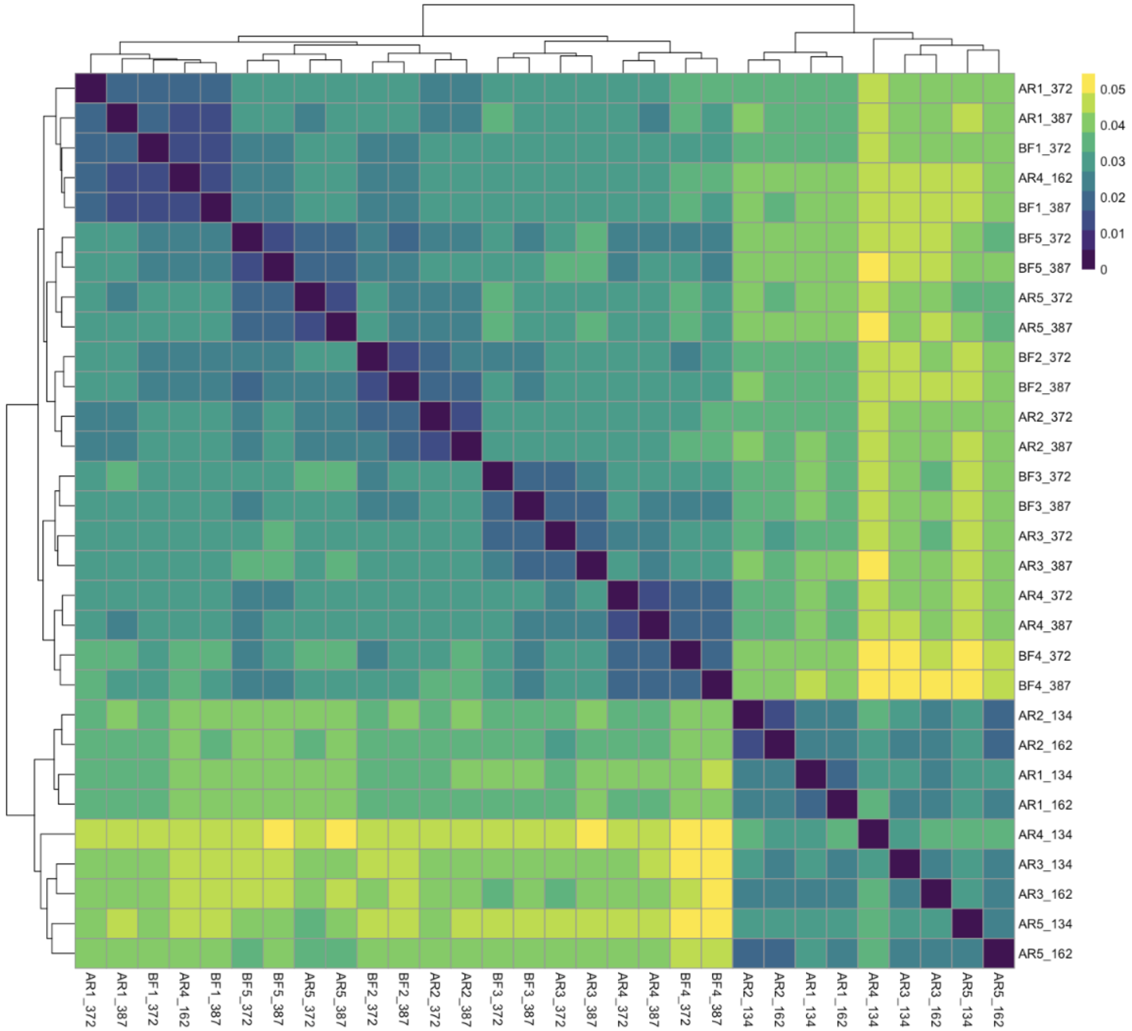

Supplementary Figure 5: **Heatmap of MASH Distances of Reads Between Sequenced Samples**

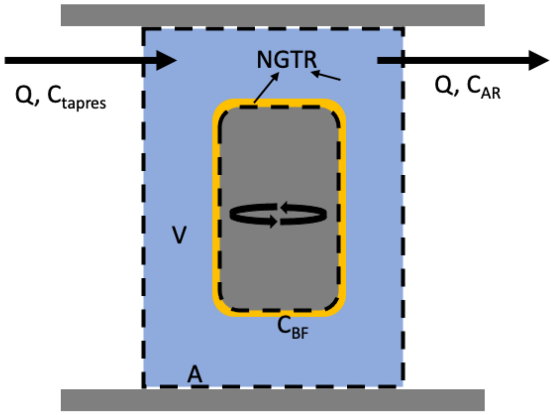

Supplementary Figure 6: Number Balance Diagram. Number balance of intact cells within a reactor with labeled components and flows. The control volume is within the two dashed lines (includes bulk water and biofilm on the PVC slides on the inner cylinder). Arrows depict cells

entering the reactor through advective flow from the tap reservoir and cells leaving the reactor through advective flow.

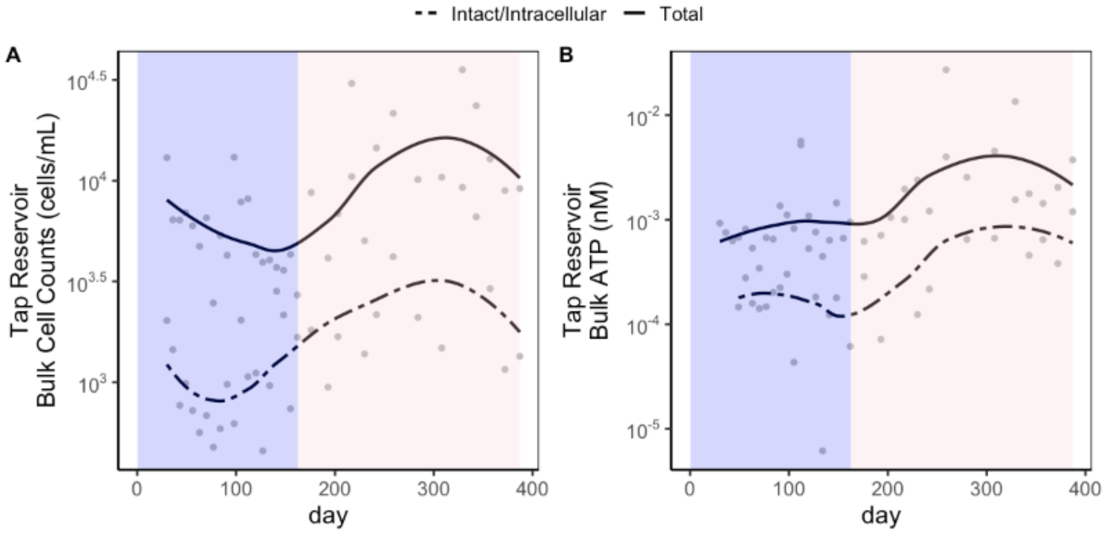

Supplementary Figure 7: Time Series of Tap Reservoir Cell Counts and ATP. Tap reservoir total/intact cell counts and total/intracellular ATP concentrations over time.

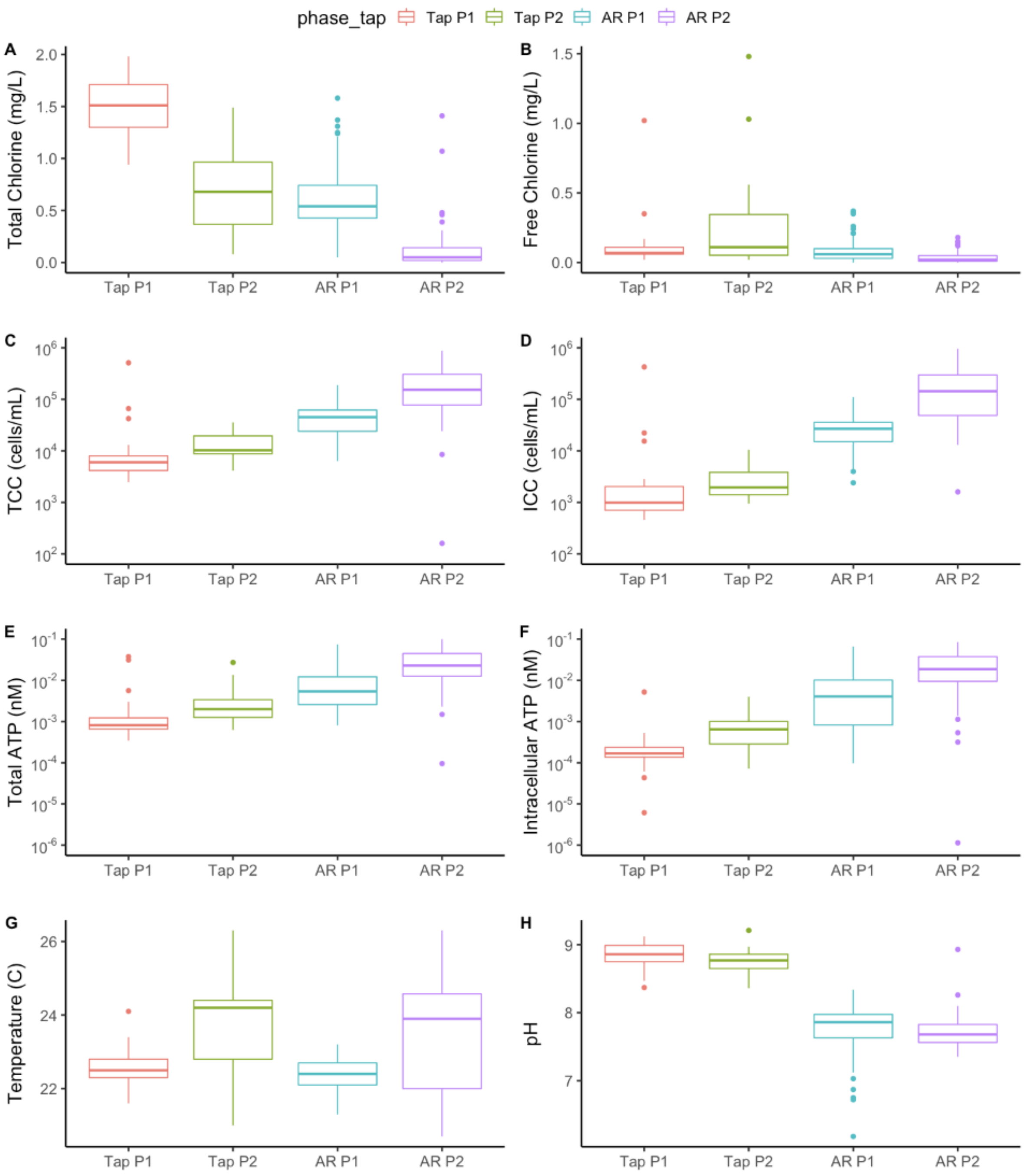

Supplementary Figure 8: Boxplots of bulk water quality parameters. Includes cell counts and ATP concentrations in Phase I versus Phase II in both the tap reservoir and the reactors. Box edges correspond to the first quartile, median, and third quartile. Whiskers extend from the edge to the largest and smallest values no further than 1.5× the interquartile range from the edge. Data beyond the whiskers are considered outliers and plotted individually.

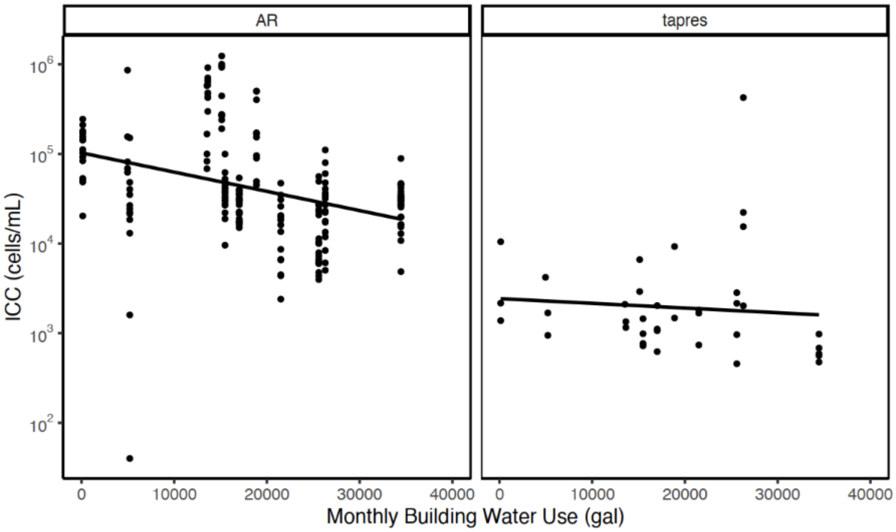

Supplementary Figure 9: Cell Counts vs. Water Usage. ICC in the reactor bulk water and tap reservoir versus monthly building water use based on the shared building water meter.

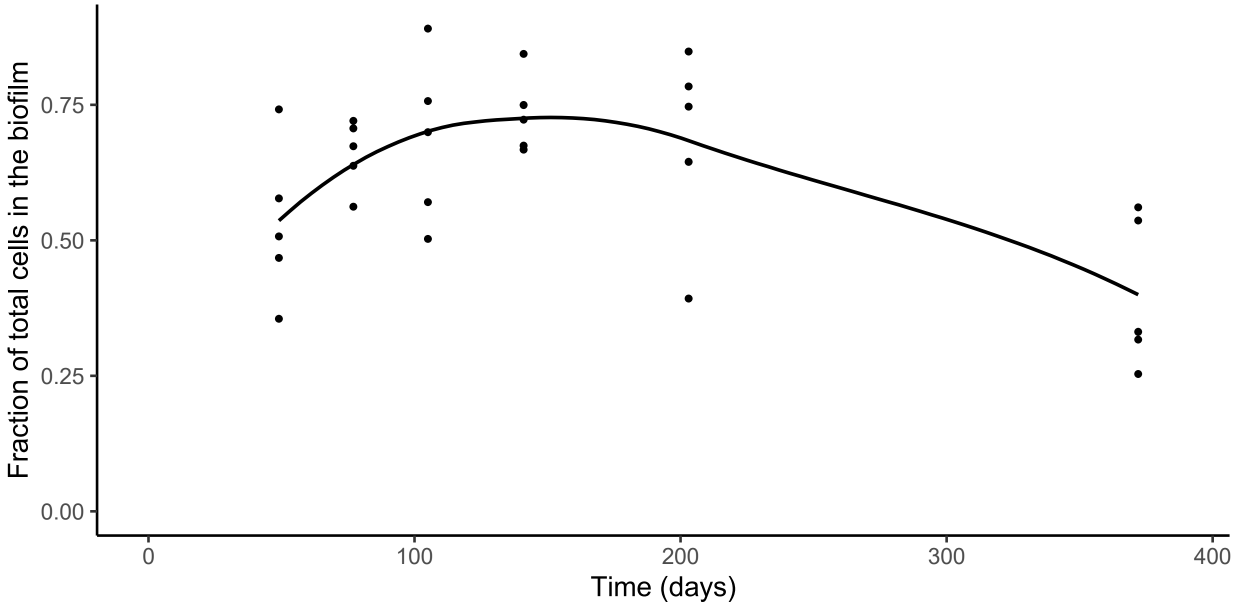

Supplementary Figure 10: Biofilm Cell Fraction Time Series. Intact cells in the biofilm were calculated by multiplying the ICC per area by the total area of PVC coupons in the reactor. Intact cells in the bulk water were calculated by multiplying the concentration by the reactor volume. Total cells were calculated by adding together the intact cells in the biofilm and intact cells in the bulk water.

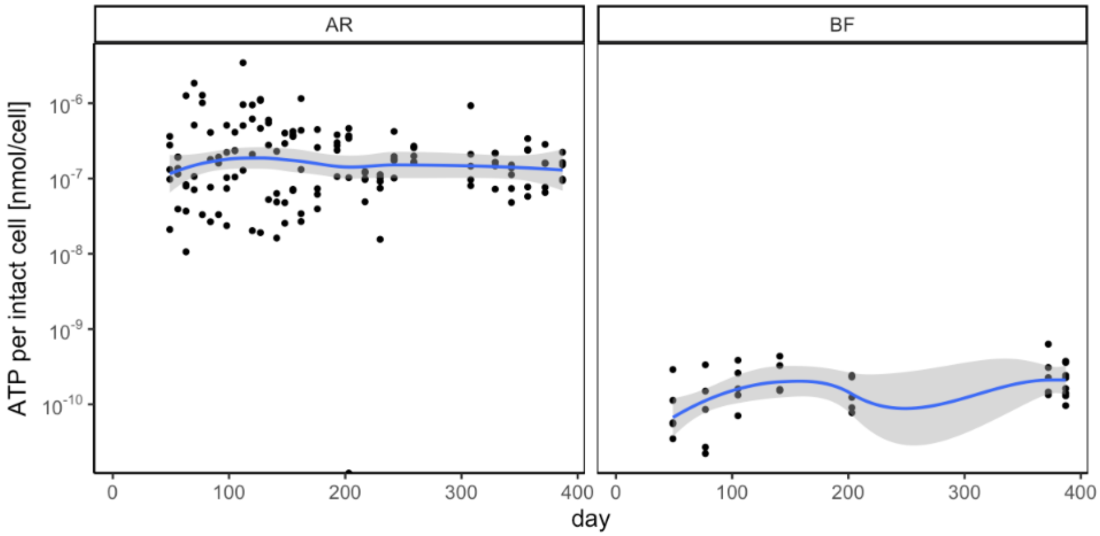

Supplementary Figure 11: Intracellular ATP per Intact Cell Count. Time series for bulk water and biofilm.

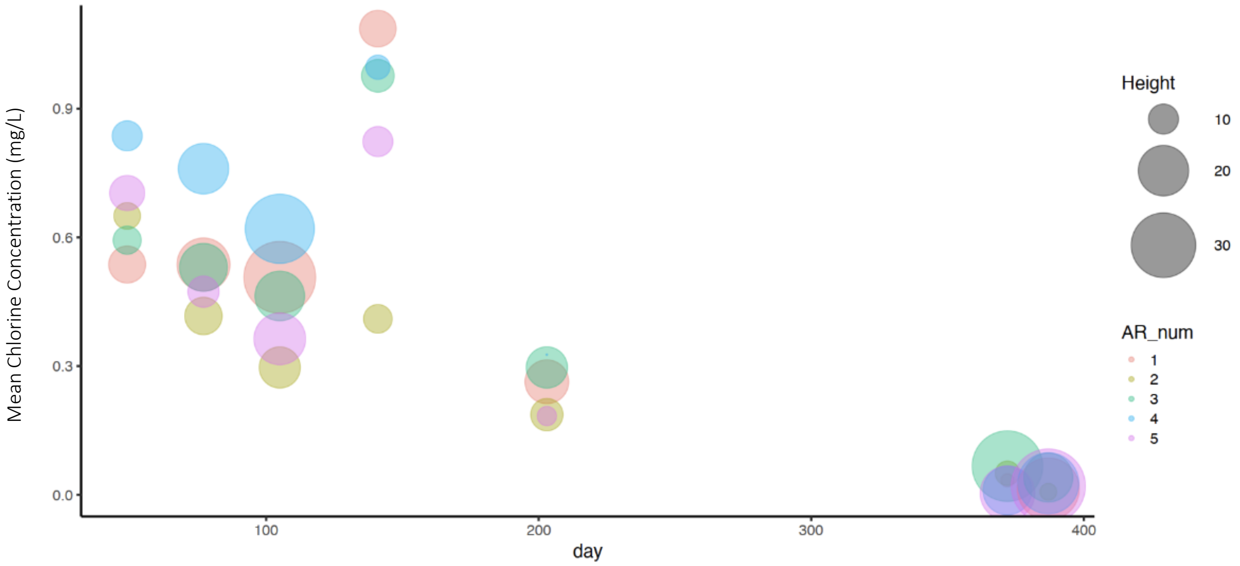

Supplementary Figure 12: Maximum Biofilm Height vs. Chlorine vs. Time. Maximum height of biofilm as a function of chlorine residual concentration and time. The maximum biofilm height is based on quantitative image analysis of CLSM z-stacks and represented by circle size, with day on the x axis and the mean of the three preceding total chlorine measurements on the y axis.

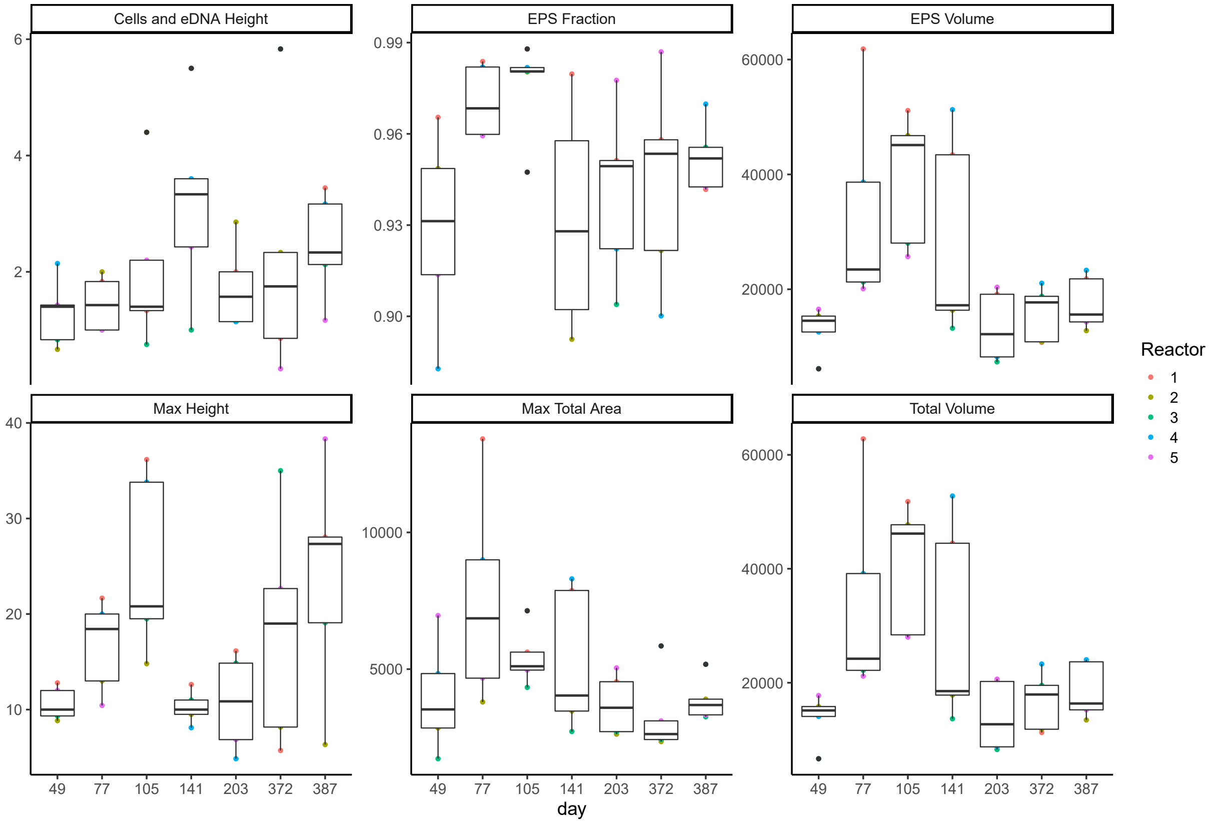

Supplementary Figure 13: Output Parameters of Biofilm Images. Output parameters of biofilm over time from quantitative image analysis of fluorescent confocal laser scanning microscopy images. Parameters were averaged across all images captured for a single sample. Extracellular polymeric substance (EPS) parameters were based on signal from SYPRO Orange and ConA. Height of cells and eDNA was based on signal from SYTO9 dye. Units of height, area, and volume are in µm, μm^2, and μm^3, respectively. Box edges correspond to the first quartile, median, and third quartile. Whiskers extend from the edge to the largest and smallest values no further than 1.5× the interquartile range from the edge. Data beyond the whiskers are considered outliers and plotted individually.

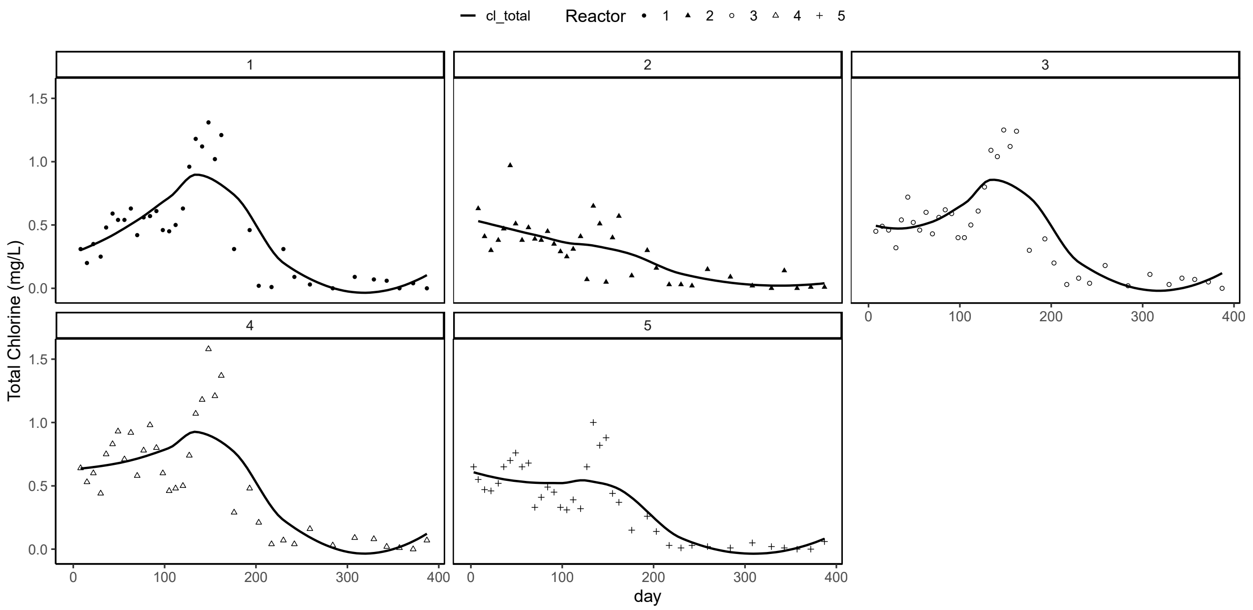

Supplementary Figure 14: Total Chlorine Time Series. Total Chlorine by reactor over the days since the start of the experiment.

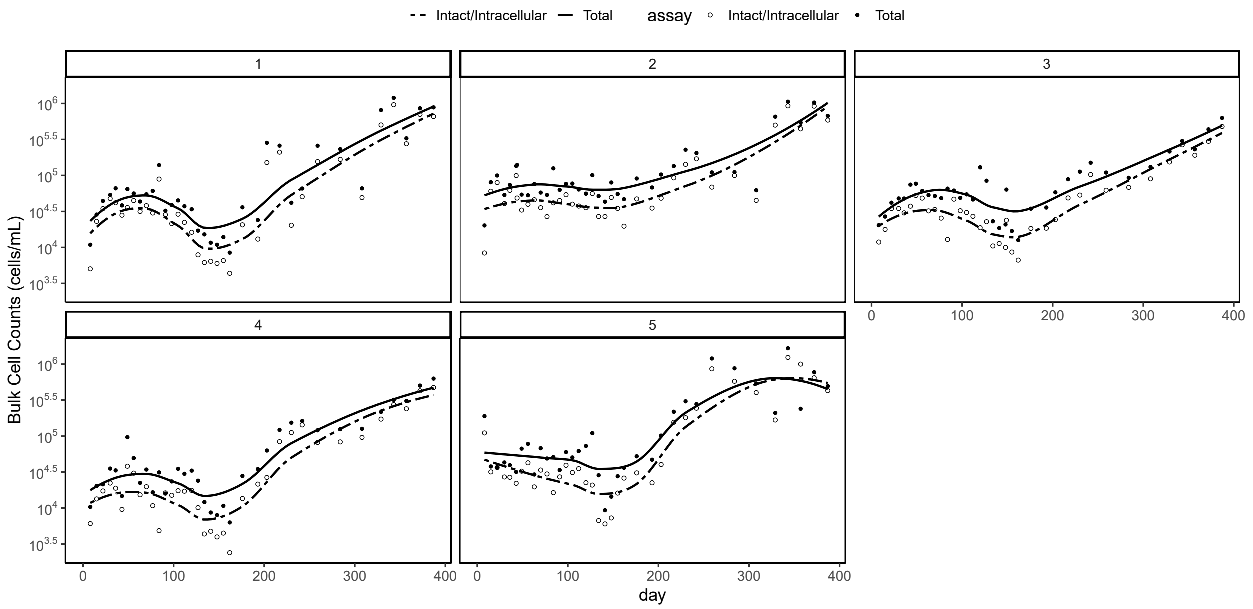

Supplementary Figure 15: **Bulk Cell Counts Time Series.** Bulk cell counts by reactor over the days since the start of the experiment.

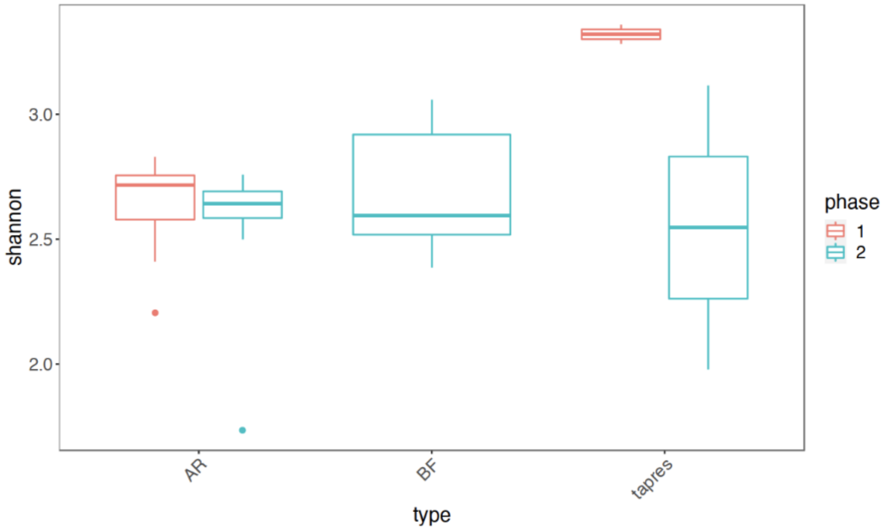

Supplementary Figure 16: Alpha Diversity using Shannon Diversity Index. Alpha diversity by sample type and phase. Box edges correspond to the first quartile, median, and third quartile. Whiskers extend from the edge to the largest and smallest values no further than 1.5× the interquartile range from the edge. Data beyond the whiskers are considered outliers and plotted individually.

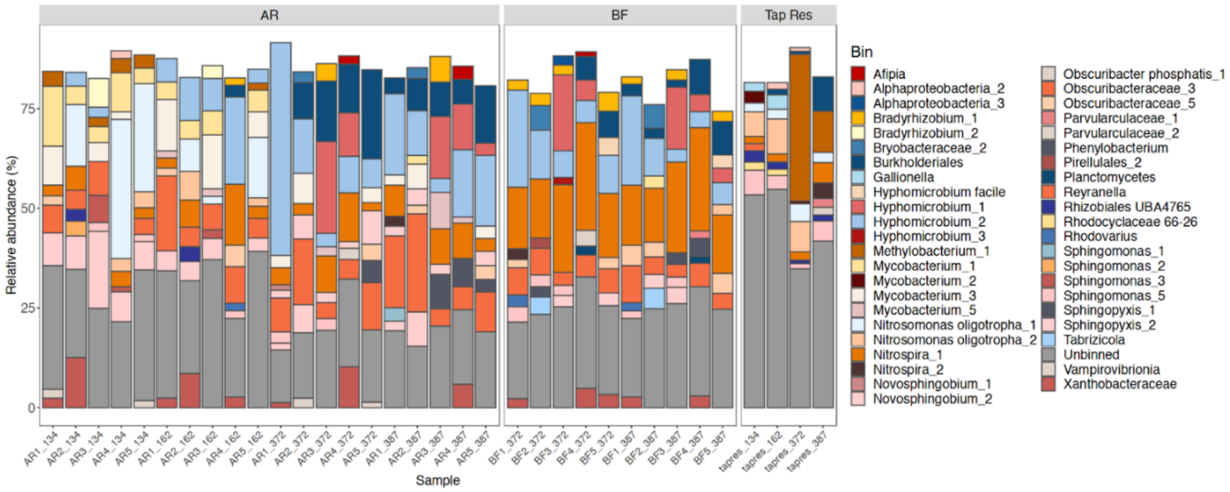

Supplementary Figure 17: MAGs Relative Abundance. Barplot of the Ten Most Abundant MAGs in Each Sample.

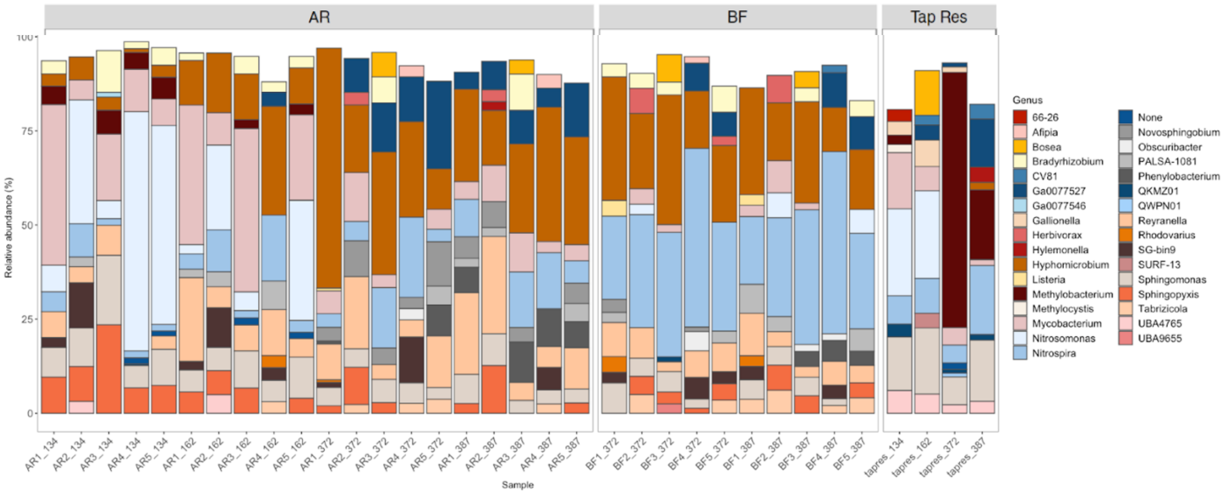

Supplementary Figure 18: Taxa Relative Abundance. Barplot of the Ten Most Abundant Taxa in Each Sample identified via the Single Copy Gene RPS2.

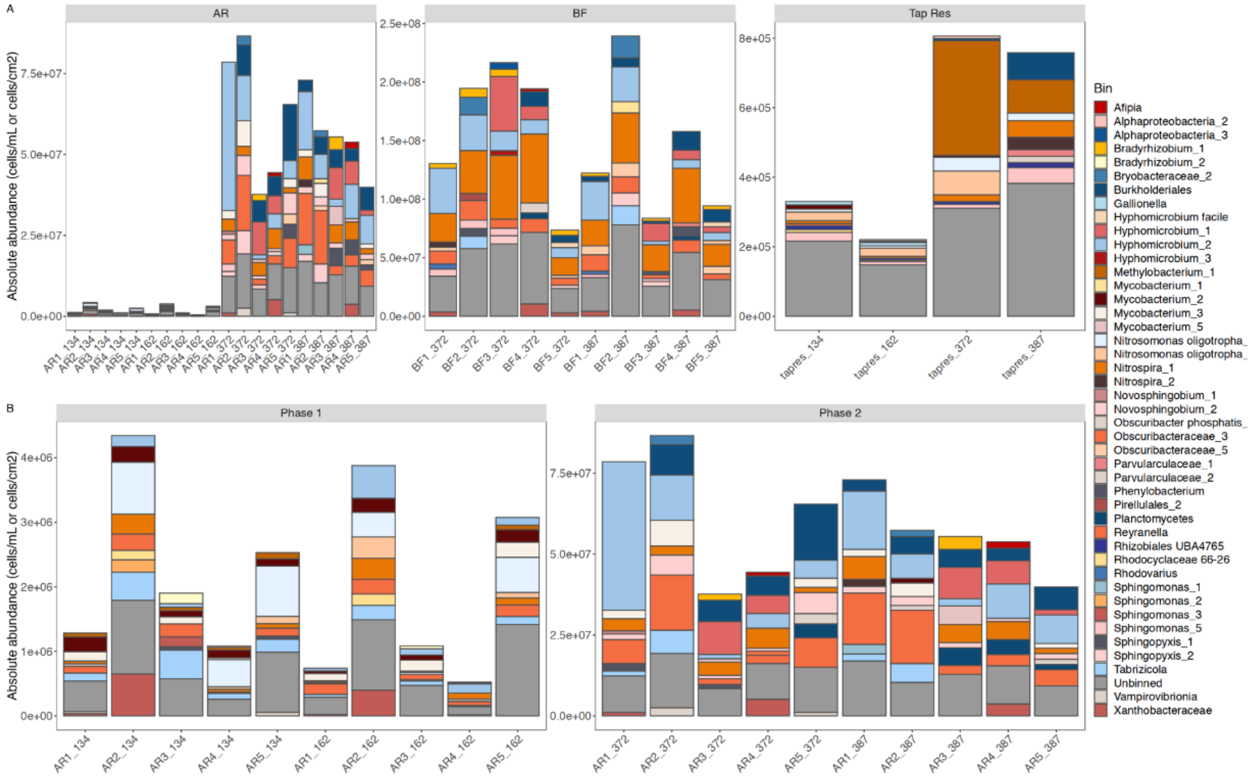

Supplementary Figure 19: MAGs Absolute Abundance. Absolute abundance calculated by relative abundance multiplied by TCC. (**A**) Reactor bulk, reactor biofilm, and tap reservoir absolute abundance barplots. (**B**) A closer view of bulk reactor samples on discrete scales to allow comparison across Phase I and Phase II. Because a relatively high fraction of reads did not map to dereplicated bins (>10%), relative and absolute abundances were scaled to account for the fraction of unbinned reads. The abundances of unbinned reads are presented along with the MAGs (see legend).

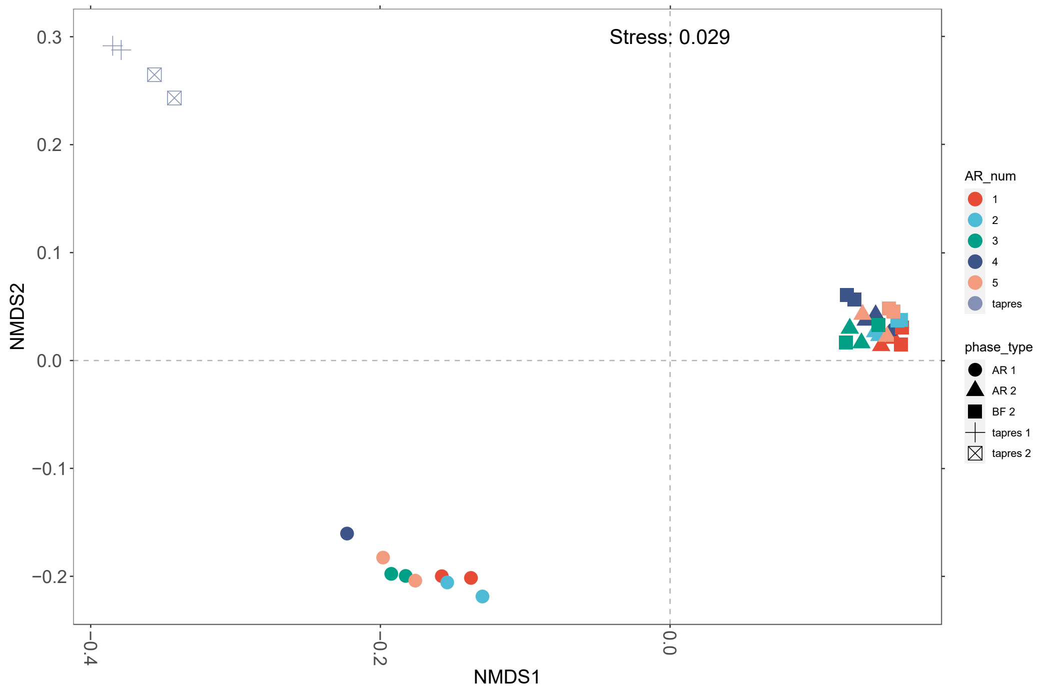

Supplementary Figure 20: NMDS Ordination of MASH Distances of Reads. Sample types are represented by AR for reactor bulk water, BF for reactor biofilm, and tapres for tap reservoir. The sample source is labeled by reactor number or tapres for tap reservoir.

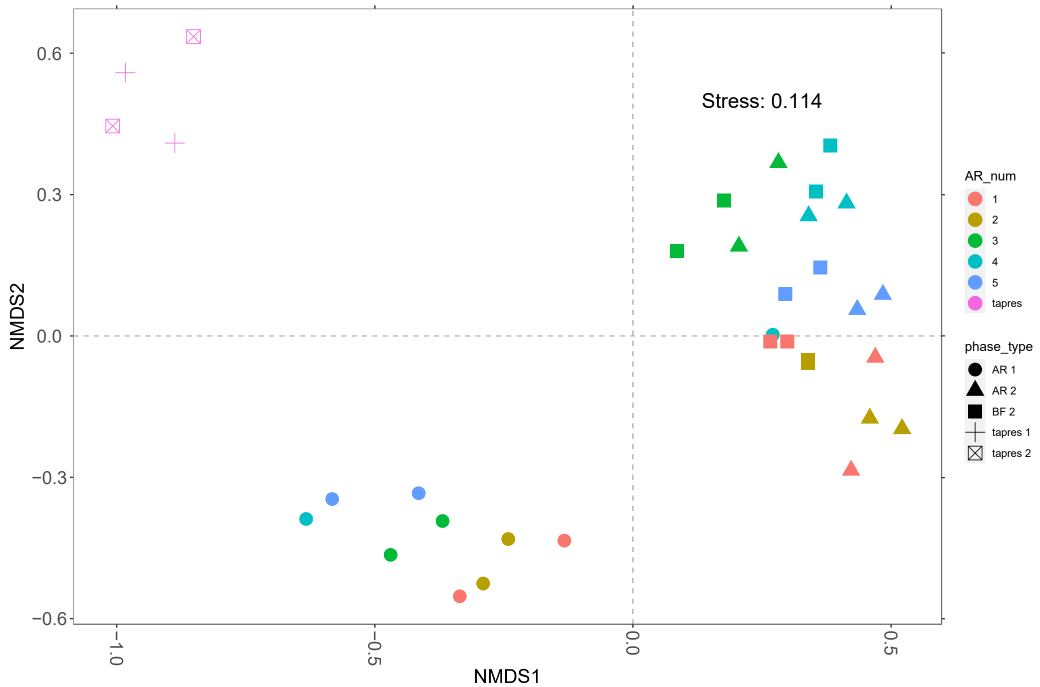

Supplementary Figure 21: Bray-Curtis Distances of MAGs. Sample types are represented by AR for reactor bulk water, BF for reactor biofilm, and tapres for tap reservoir. The sample source is labeled by reactor number or tapres for tap reservoir.

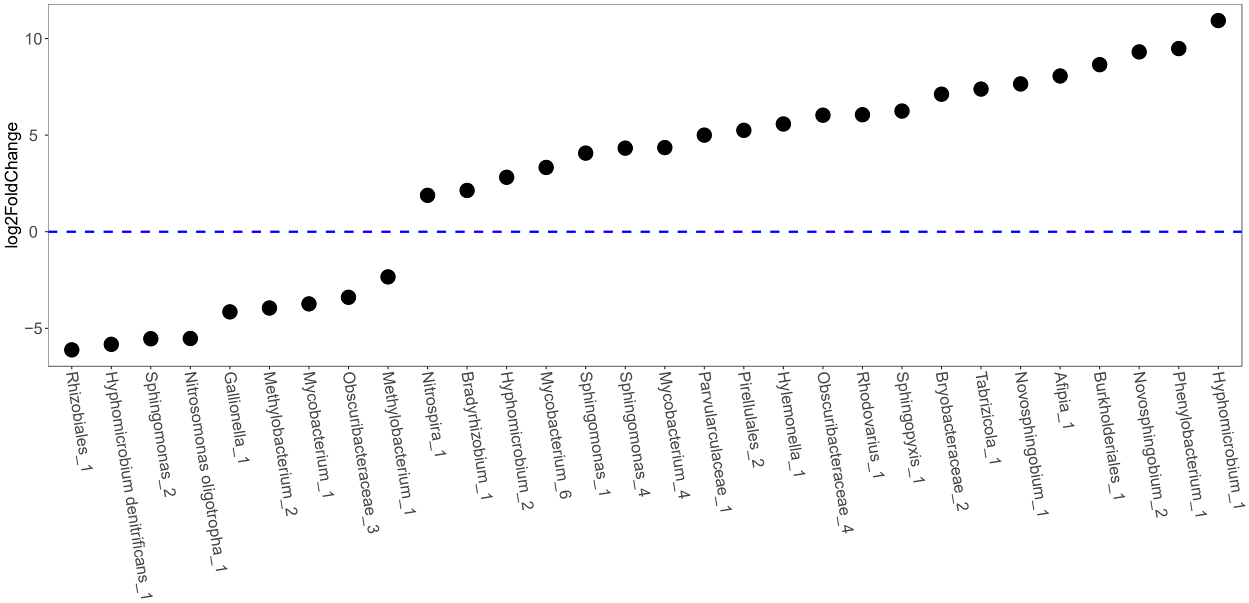

Supplementary Figure 22: Enriched MAGs Phase II vs. Phase I. Enriched MAGs in the AR bulk water between Phase I (log2FoldChange < 0) and Phase II (log2FoldChange > 0) based on differential abundance analysis with DESeq2. Sample AR4_162 was not included in the analysis because of suspected contamination.

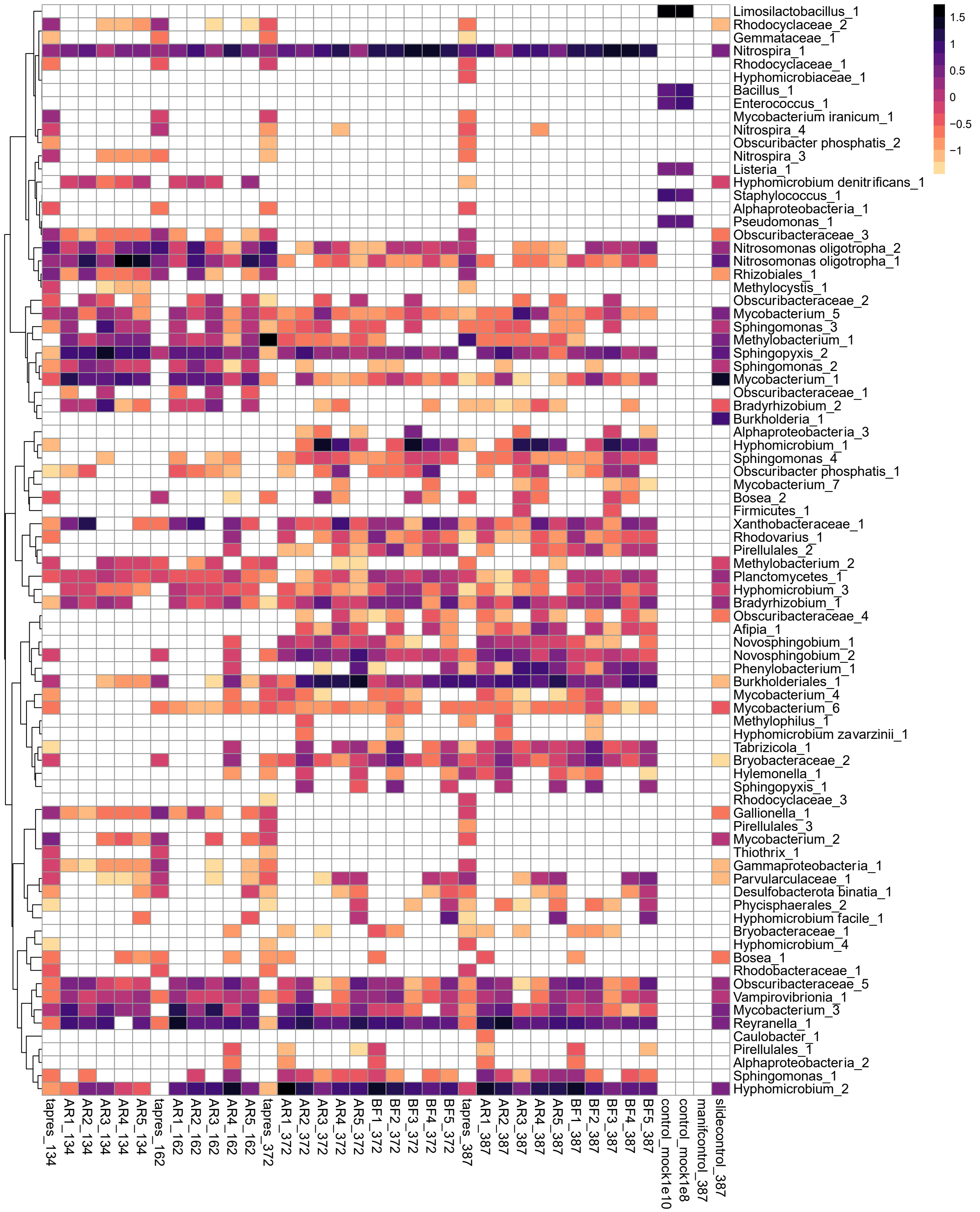

Supplementary Figure 23: Heatmap of All MAGs. Heatmap of the normalized relative abundance (log10) of all the dereplicated bins across all sequenced samples and negative controls.

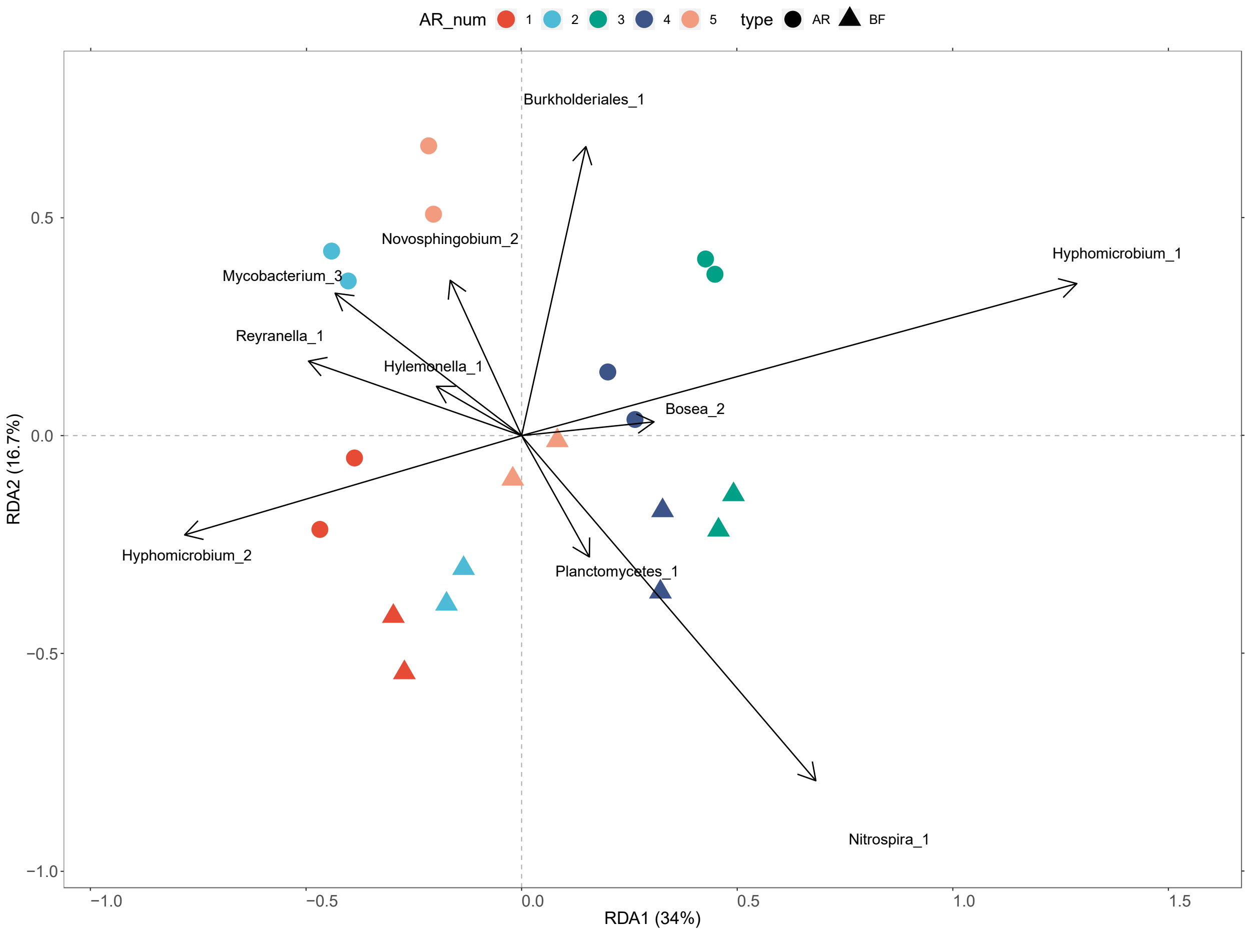

Supplementary Figure 24: RDA Plot of Phase II. Ordination of RDA of reactor bulk and biofilm samples from Phase II only.

Supplementary Tables

Supplementary Table 1: Sampling Events. Sample counts for each assay by sample type.

		AR Bulk Water			Biofilm	Tap Res			Tap			All
	**Day**	**Chlorine, pH, Temp.**	**TCC/ ICC**	**ATPt/ ATPi**	**TCC/ICC, CLSM**	**Chlorine, pH, Temp.**	**TCC/ ICC**	**ATPt/ ATPi**	**Chlorine, pH, Temp.**	**TCC/ ICC**	**ATPt/ ATPi**	**Metagenomics/ qPCR**
Phase I	8	5	5	ATPt only	NA	1	1	ATPt only	NA	NA	NA	NA
	15	5	5	ATPt only	NA	1	1	ATPt only	NA	1	NA	NA
	22	5	5	ATPt only	NA	1	1	ATPt only	NA	NA	NA	NA
	30	5	5	ATPt only	NA	1	1	ATPt only	NA	NA	NA	NA
	36	5	5	ATPt only	NA	1	1	ATPt only	NA	NA	NA	NA
	43	5	5	ATPt only	NA	1	1	ATPt only	NA	NA	NA	NA
	44	NA	1	1	NA	NA	NA	NA	NA	NA	NA	NA
	49	5	5	5	5	1	1	1	NA	NA	NA	NA
	56	5	5	5	NA	1	1	1	NA	NA	NA	NA
	63	5	5	5	NA	1	1	1	NA	NA	NA	NA
	70	5	5	5	NA	1	1	1	NA	NA	NA	NA
	77	5	5	5	5	1	1	1	NA	NA	NA	NA
	84	5	5	5	NA	1	1	1	NA	NA	NA	NA
	91	5	5	5	NA	1	1	1	NA	NA	NA	NA
	98	5	5	5	NA	1	1	1	1	NA	NA	NA
	105	5	5	5	5	1	1	1	1	1	1	NA
	112	5	5	5	NA	1	1	1	1	NA	ATPt only	NA
	120	5	5	5	NA	1	1	1	1	1	1	NA
	127	5	5	5	NA	1	1	1	1	NA	ATPt only	NA
	134	5	5	5	NA	1	1	1	1	1	1	5 AR<br>1 Tap Res
	141	5	5	5	5	1	1	1	1	NA	ATPt only	NA
	148	5	5	5	NA	1	1	1	1	1	1	NA
	149	NA	1	1 ATPt only	NA	1	1	1	NA	NA	NA	NA
	155	5	5	5	NA	1	1	1	1	NA	ATPt only	NA
	162	5	5	5	NA	1	1	1	1	1	1	5 AR<br>1 Tap Res
Phase II	163	5	NA	3 ATPt only	NA	1	1	1	NA	NA	NA	NA
	176	5	5	5	NA	1	1	1	NA	1	ATPt only	NA

193	5	5	5	NA	1	1	1	NA	NA	NA	NA
203	5	5	5	5	1	1	1	NA	NA	NA	NA
217	5	5	5	NA	1	1	1	1	1	1	NA
230	Cl only	5	5	NA	Cl only	1	1	Cl only	1	1	NA
242	5	5	5	NA	1	1	1	1	1	1	NA
259	5	5	5	NA	1	1	1	1	1	1	NA
284	5	5	5	NA	1	1	1	1	1	1	NA
308	5	5	5	NA	1	1	1	1	1	1	NA
329	5	5	5	NA	1	1	1	1	1	1	NA
343	5	5	5	NA	1	1	1	1	1	1	NA
357	5	5	5	NA	1	1	1	1	1	1	NA
372	5	5	5	5	1	1	1	1	1	1	5 AR<br>5 Biofilm<br>1 Tap Res
387	5	5	5	5 (9 CLSM slides)	1	1	1	1	1	1	5 AR<br>5 Biofilm<br>1 Tap Res
Total	186	187	190	30	39	39	39	21	18	21	34

Supplementary Table 2: Metagenomics positive control mock community composition. Two positive controls were extracted and sequenced, with starting concentrations of 1×10^8 cells/mL (1×10^7 total cells extracted) and 1×10^{10} cells/mL (1×10^9 cells extracted).

	Theoretical composition (%) based on:				
**species**	**gDNA**	**16S**	**16S&18S**	**genome copy**	**cell number**
P. aeruginosa	12	4.2	3.6	6.1	6.1
E. coli	12	10.1	8.9	8.5	8.5
S. enterica	12	10.4	9.1	8.7	8.8
L. fermentum	12	18.4	16.1	21.6	21.9
E. faecalis	12	9.9	8.7	14.6	14.6
S. aureus	12	15.5	13.6	15.2	15.3
L. monocytogenes	12	14.1	12.4	13.9	13.9
B. subtilis	12	17.4	15.3	10.3	10.3
S. cerevisiae	2	NA	9.3	0.57	0.29
C. neoformans	2	NA	3.3	0.37	0.18

Supplementary Table 3: qPCR reaction concentrations and volumes. Nuclease-free water was added to each reaction to bring the total volume up to 20 µL (*amoA*) or 10 µL (all other assays) after the addition of other reagents.

**Reagent**	***mip***	***amoA***	***oprL***	**MACF/MACR**
Forward and Reverse Primers	0.9 µM	0.7 µM	0.2 µM	0.9 µM
Probe	0.25 µM	NA	0.2 µM	NA
Bovine serum albumen (BSA)	0.05 mg/L	0.05 mg/L	0.05 mg/L	0.05 mg/L
Mastermix	1x	1x	1x	1x
Sample DNA extract volume	1 µL	5 µL	1 µL	1 µL
Total reaction volume	10 µL	20 µL	1 µL	1 µL

Supplementary Table 4: qPCR primers, probes, and standards.

**Assay (Ref.)**	**Master-mix**	**Primers/Probe**	**Amplicon Sequence/Length**	**Cycling Conditions**
mip (Nazarian et al., 2008)	Taqman Environmental 2.0	**F**:AAAGGCATGCAAGACGCTATG **R**:GAAACTTGTTAAGAACGTCTTTCATTTG **P**:TGGCGCTCAATTGGCTTTAACCGA	AAAGGCATGCAAGACGCTATGAGTGGCGCTCAATTGGCTTTAACCGAACAGCAAATGAAAGACGTTCTTAACAAGTTTC **(79 bp)**	10 min at 95°C; 40 cycles of 15 sec at 95°C, 1 min at 60°C
amoA (Rotthauwe et al., 1997)	PowerUp Sybr Green	**F**: GGGGTTTCTACTGGTGGT **R**:CCCCTCKGSAAAGCCTTCTTC **P**: NA	GGGGTTTCTACTGGTGGTCACATTACCCAATCAACTTTGTCACACCGGGCATCATGCTACCAGGTGCGTTGATGCTGGATCTGACCTTGTATTTGACACGTAATTTCTTGATTACGGCGCTACTTGGTGGTGCATTTTTTGGTTTATTGTTCTATCCGGGCAACTGGACAATATTTGGACCTACGCACTTGCCGATTGTAGTAGAAGGGCACTTATTGTCGATGGCCGATTATATGGGTCACCTGTACATTCGCACAGGTACGCCAGAATACACTCGCTTGATTGAGAAAGGGTCATTACGTACCTTTGGTGGTCATACCACAGTAATTGCGGCATTTTTTGCATCGTTTGTCTCTATGCTGGTATTTTTGGTGTGGTGGTATCTGGGTAAAGTCTATTGCACAGCTTTCTTCTATGTCAAAGGTAAAAGAGGTCGTATTGTACATAGAGAAGACGTGACTGCATTTGGTGAAGAAGGCTTTCCCGAGGGG **(491 bp)**	2 min at 50°C; 2 min at 95°C; 40 cycles of 30 sec at 95°C, 30 sec at 60°C, 1 min at 72°C; Melt curve
oprL (Feizabadi et al., 2010)	Taqman Environmental 2.0	**F**:CGAGTACAACATGGCTCTGG **R**:ACCGGACGCTCTTTACCATA **P**:CCTGCAGCACCAGGTAGCGC	CGAGTACAACATGGCTCTGGGCGAGCGTCGTGCCAAGGCCGTTCAGCGCTACCTGGTGCTGCAGGGTGTTTCGCCGGCCCAGCTGGAACTGGTTTCCTATGGTAAAGAGCGTCCGGT **(117 bp)**	5 min at 95°C; 40 cycles of 20 sec at 95°C, 20 sec at 57.8°C, 20 sec at 72°C

MAC (Whiley et al., 2014)	PowerUp Sybr Green	**F**:CCCTGAGACAACACTCGGTC<br>**R**:ATTACACATTTCGATGAACGC<br>**P**: NA	CCCTGAGACAACACTCGGTCGATCCGTGTGGAGTCCCTCCATCTTGGTGGTGGGGTGTGGTGTTTGAGTATTGGATAGTGGTTGCGAGCATCTAGATGAGCGCGTAGTCCTTCGTGGCTGATGCGTTCATCGAAATGTGTAAT **(143 bp)**	2 min at 50°C; 5 min at 95°C; 40 cycles of 15 sec at 95°C, 30 sec at 55°C, 1 min at 72°C; Melt curve

Supplementary Table 5: qPCR plate quality. Summarizes standard curve parameters and NTCs

Assay	Plate	Efficiency	Slope	y-intercept	R^2	NTCs Amplified
mip	1	90.7%	-3.568	38.972	0.993	0
	2	95.4%	-3.437	37.847	0.995	0
amoA	1	89.8%	-3.594	36.59	0.994	0
	2	92.9%	-3.505	36.496	0.998	0
oprL	1	99.1%	-3.343	38.912	0.994	0
	2	114.6%	-3.016	37.713	0.985	0
MACF/MACR	1	90.4%	-3.575	37.779	0.998	0
	2	92.8%	-3.507	38.893	0.984	0

Supplementary Table 6: Limits of detection and quantification per assay.
Limit of detection (LoD) was defined as the interpolated copy number at which 95% of replicates amplified. Limit of quantification (LoQ) was defined as the interpolated copy number at which the coefficient of variation equalled 35%. If the coefficient of variation never exceeded 35% or went below 35% at a value less than the LoD, the LoQ was set to the LoD.

qPCR Assay	LoD (gc/reaction)	LoQ (gc/reaction)
mip	4.1	4.4
amoA	9	9
oprL	9	53
MACF/MACR	4.3	5.7

Supplementary Table 7: qPCR Inhibition Testing. A distribution of samples across reactors and time points were selected for inhibition testing. For each assay, two biofilm and two bulk water samples were selected: one with high DNA concentration based on Qubit readings and one with low. Samples at each dilution were considered inhibited if dCt relative to the previous dilution was greater than one. Only one sample showed inhibition at any dilution (*oprL* assay, AR4_372) which was diluted out at 4x.

Assay	Sample type	Sample ID	Dilution	Mean Cq	Expected relative to previous	Experimental dCt relative to previous	abs(expected - actual) [dCt relative to previous]	Classification
amoA	AR low DNA	AR4_134	1	19.7	0	0		not inhibited
			2	20.29	1	0.58	**0.42**	not inhibited
			10	23.05	2.32	2.76	**0.44**	not inhibited
			50	25.41	2.32	2.36	**0.04**	-
	AR high DNA	AR3_372	1	19.26	0	0		not inhibited
			2	20.36	1	1.1	**0.1**	not inhibited
			10	22.81	2.32	2.45	**0.13**	not inhibited
			50	24.82	2.32	2.01	**0.31**	-
	BF low DNA	BF4_372	1	19.99	0	0		not inhibited
			2	20.85	1	0.87	**0.13**	not inhibited
			10	23.04	2.32	2.19	**0.13**	not inhibited
			50	25.54	2.32	2.49	**0.17**	-
	BF high DNA	BF3_387	1	20.23	0	0		not inhibited
			2	20.52	1	0.29	**0.71**	not inhibited
			10	23	2.32	2.48	**0.16**	not inhibited
			50	25.23	2.32	2.23	**0.09**	-
oprL	AR low DNA	AR5_134	1	24.3	0	0		not inhibited
			2	24.89	1	0.6	**0.4**	not inhibited
			4	25.64	1	0.75	**0.25**	not inhibited
			8	26.08	1	0.44	**0.56**	-
	AR high DNA	AR4_372	1	22.97	0	0		inhibited
			2	25.6	1	2.62	**1.62**	inhibited
			4	25.12	1	-0.48	**1.48**	not inhibited
			8	26.82	1	1.7	**0.7**	-
	Bf low DNA	BF5_372	1	23.76	0	0		not inhibited
			2	25.32	1	1.56	**0.56**	not inhibited
			4	25.39	1	0.07	**0.93**	not inhibited
			8	25.69	1	0.3	**0.7**	-

	Bf high DNA	BF4_387	1	23	0	0		not inhibited
			2	24.58	1	1.58	**0.58**	not inhibited
			4	25.03	1	0.45	**0.55**	not inhibited
			8	26.76	1	1.73	**0.73**	-
mip	AR low DNA	AR3_134	1	21.27	0	0		not inhibited
			2	22.2	1	0.93	**0.07**	not inhibited
			10	24.68	2.32	2.48	**0.15**	not inhibited
			50	26.85	2.32	2.17	**0.15**	-
	AR high DNA	AR2_372	1	21.38	0	0		not inhibited
			2	22.24	1	0.87	**0.13**	not inhibited
			10	24.56	2.32	2.32	**0**	not inhibited
			50	26.79	2.32	2.23	**0.09**	-
	Bf high DNA	BF2_387	1	20.81	0	0		not inhibited
			2	21.75	1	0.94	**0.06**	not inhibited
			10	24.1	2.32	2.35	**0.03**	not inhibited
			50	26.57	2.32	2.47	**0.15**	-
MACF/ MACR	AR low DNA	AR_1_134	1	19.27	0	0		not inhibited
			2	20.2	1	0.93	**0.07**	not inhibited
			10	22.6	2.32	2.4	**0.08**	not inhibited
			50	24.77	2.32	2.17	**0.16**	-
	AR high DNA	AR1_372	1	19.35	0	0		not inhibited
			2	20.41	1	1.06	**0.06**	not inhibited
			10	22.73	2.32	2.32	**0**	not inhibited
			50	25.12	2.32	2.39	**0.07**	-
	BF low DNA	BF2_372	1	19.21	0	0		not inhibited
			2	20.34	1	1.14	**0.14**	not inhibited
			10	22.67	2.32	2.33	**0.01**	not inhibited
			50	24.91	2.32	2.23	**0.09**	-
	BF high DNA	BF1_387	1	19.2	0	0		not inhibited
			2	20.17	1	0.97	**0.03**	not inhibited
			10	22.6	2.32	2.43	**0.11**	not inhibited
			50	25.1	2.32	2.5	**0.18**	-

Supplementary Table 8: Sequencing Quality by Sample. Information for each Sequenced Sample and Control

Sample ID	Count of Raw Forward Reads	Count of Trimmed Forward Reads	Trimmed bps	N50	Scaffold Count	Assembly Size (bp)	Count of total reads mapped	Count of reads mapped to derep. genomes	Percent of reads mapped to derep. genomes
AR1_134	26391193	26273730	7.73E+09	16075	76242	1.49E+08	48468409	36191474	68.87
AR1_162	27493842	27377695	8.08E+09	10576	78893	1.54E+08	50832749	37276741	68.08
AR1_372	32573758	32362661	9.60E+09	10862	83621	1.67E+08	62510558	56106457	86.68
AR1_387	45065764	44845339	1.33E+10	7788	128121	2.33E+08	84662268	72486165	80.82
AR2_134	37543974	37390867	1.10E+10	18682	79234	1.79E+08	68649513	58250954	77.89
AR2_162	34751616	34581359	1.02E+10	10983	102348	1.86E+08	59617924	53040286	76.69
AR2_372	29552650	29308082	8.70E+09	42946	70093	1.60E+08	56842459	49046712	83.67
AR2_387	43923976	43649070	1.29E+10	28897	81784	1.78E+08	85287824	73898639	84.65
AR3_134	42645590	42449515	1.25E+10	18632	98036	1.84E+08	79049647	63803481	75.15
AR3_162	50243555	49984555	1.48E+10	19962	113300	1.95E+08	91619708	62787469	62.81
AR3_372	41513821	41241292	1.22E+10	10128	95957	1.79E+08	77828246	66511468	80.64
AR3_387	37541537	37355036	1.11E+10	20305	86669	1.66E+08	70812522	59428729	79.55
AR4_134	29074701	28936051	8.28E+09	6744	95368	1.57E+08	50675474	45358005	78.38
AR4_162	55895292	55644314	1.64E+10	13678	134456	2.66E+08	101231738	89422464	80.35
AR4_372	54157417	53805836	1.59E+10	13003	115319	2.33E+08	100137684	83955726	78.02
AR4_387	36058555	35846476	1.06E+10	10178	97103	2.14E+08	67149377	58253297	81.25
AR5_134	28011485	27871385	8.09E+09	6748	108426	1.86E+08	50025362	37501794	67.28
AR5_162	38386252	38177835	1.12E+10	10308	143143	2.47E+08	68360975	46472776	60.86
AR5_372	42448501	42212668	1.25E+10	31487	111229	2.20E+08	81636913	69198286	81.96
AR5_387	42099209	41873687	1.24E+10	20264	112528	2.21E+08	80559759	67859506	81.03
BF1_372	46096198	45876858	1.35E+10	13499	115899	2.42E+08	83368202	74151345	80.82
BF1_387	33708775	33521206	9.97E+09	13476	117136	2.26E+08	60814201	53881379	80.37
BF2_372	28034781	27921668	8.23E+09	22672	97667	1.92E+08	52305168	42811903	76.66
BF2_387	30451001	30311231	8.84E+09	30695	100675	2.16E+08	56681883	45606742	75.23
BF3_372	31239635	31101250	9.16E+09	15608	90457	1.67E+08	56527701	46495305	74.75
BF3_387	35279618	35124908	1.03E+10	35370	92501	1.77E+08	64136115	51906860	73.89
BF4_372	23445210	23320685	6.86E+09	11191	94632	1.57E+08	40972005	33612429	72.07
BF4_387	37356483	37150807	1.09E+10	12926	110792	2.04E+08	65182312	53976212	72.64
BF5_372	33411248	33268104	9.70E+09	24595	106174	2.10E+08	61904196	51673150	77.66
BF5_387	39260600	39103449	1.13E+10	32211	100518	2.27E+08	72564158	58915616	75.33
slidecontrol_387	43086819	42879980	1.23E+10	12617	119057	2.12E+08	78842467	68881006	80.32
manifcontrol_387	309374	295935	8.62E+07	1826	3458	3.42E+06	216949	98071	16.57
control_mock1e10	30282749	30126000	8.93E+09	22602	22625	5.84E+07	58412621	37986476	63.05
control_	29899593	29758759	8.63E+09	71914	25485	4.48E+07	57461794	39078170	65.66

mock1e8									
tapres_134	38735372	38616183	1.14E+10	1322	554239	5.12E+08	50018088	35974421	46.58
tapres_162	33761415	33603683	9.82E+09	878	284917	2.16E+08	32827887	30360438	45.17
tapres_372	45420641	45279563	1.34E+10	1354	316406	3.03E+08	72436440	58982049	65.13
tapres_387	39484195	39297480	1.16E+10	2281	381947	4.51E+08	62531213	45719452	58.17

Supplementary Table 9: Quality Information for Each Metagenomic Assembly.

Assembly ID	N50	Count of Scaffolds	Assembly Size (bp)	Samples in Coassembly
ARSTAG_AR_4_27	13678	134456	265896587	AR4_162
ARSTAG_ARBF_12345_pre	4540	374128	452380896	AR1_134, AR2_134, AR3_134, AR4_134, AR5_134, AR1_162, AR2_162, AR3_162, AR5_162
ARSTAG_ARBF_1_post	28507	138837	341400879	AR1_372, AR1_387, BF1_372, BF1_387
ARSTAG_ARBF_2_post	20471	141263	317594986	AR2_372, AR2_387, BF2_372, BF2_387
ARSTAG_ARBF_3_post	9117	167642	304326242	AR3_372, AR3_387, BF3_372, BF3_387
ARSTAG_ARBF_4_post	28979	140412	318849651	AR4_372, AR4_387, BF4_372, BF4_387
ARSTAG_ARBF_5_post	20823	186216	371591684	AR5_372, AR5_387, BF5_372, BF5_387
ARSTAG_TAPRES_TAPRES_23	1322	554239	511557867	tapres_134
ARSTAG_TAPRES_TAPRES_27	878	284917	216044543	tapres_162
ARSTAG_TAPRES_TAPRES_40	1354	316406	302519152	tapres_372
ARSTAG_TAPRES_TAPRES_41	2281	381947	450663496	tapres_387
ARSTAG_CONTROL_BFSLIDECONTROL_41	12617	119057	212215813	slidecontrol_387
ARSTAG_CONTROL_MANIFB_41	1826	3458	3421622	manifcontrol_387
ARSTAG_CONTROL_MOCK1E10_111821	22602	22625	58439455	control_mock1e10
ARSTAG_CONTROL_MOCK1E8_111821	71914	25485	44799459	control_mock1e8

Supplementary Table 10: Taxonomy Information and Bin Figure Names for All Dereplicated Bins.

Dereplicated Genome Bin	Bin Figure Name	Domain	Phylum	Class	Order	Family	Genus	Species
ARSTAG_AR_ 4_27_bin_23_1	Xantho- bacteraceae_1	Bacteria	Proteo- bacteria	Alphaproteo- bacteria	Rhizobiales	Xanthobacteraceae	SG-bin9	SG-bin9 sp002083525
ARSTAG_AR_ 4_27_bin_26_4	Hyphomicrobium_3	Bacteria	Proteo- bacteria	Alphaproteo- bacteria	Rhizobiales	Hypho- microbiaceae	Hyphomicrobium	Hyphomicrobium sp002928515
ARSTAG_AR_ 4_27_bin_3_2	Nitrosomonas oligotropha_1	Bacteria	Proteo- bacteria	Gammaproteo- bacteria	Burkholderiales	Nitrosomonadaceae	Nitrosomonas	Nitrosomonas oligotropha
ARSTAG_AR_ 4_27_bin_5_5	Rhodovarius_1	Bacteria	Proteo- bacteria	Alphaproteo- bacteria	Acetobacterales	Acetobacteraceae	Rhodovarius	Rhodovarius sp014376425
ARSTAG_ARBF_ 1_post_bin_10	Bryobacteraceae_1	Bacteria	Acido- bacteriota	Acidobacteriae	Bryobacterales	Bryobacteraceae	Bog-105	Bog-105 sp003169195
ARSTAG_ARBF_ 1_post_bin_18_1	Pirellulales_1	Bacteria	Plancto- mycetota	Planctomycetes	Pirellulales	Ga0077529	Ga0077529	Ga0077529 sp001464525
ARSTAG_ARBF_ 1_post_bin_2_3	Bradyrhizobium_1	Bacteria	Proteo- bacteria	Alphaproteo- bacteria	Rhizobiales	Xanthobacteraceae	Bradyrhizobium	NA
ARSTAG_ARBF_ 1_post_bin_26_2	Sphingomonas_1	Bacteria	Proteo- bacteria	Alphaproteo- bacteria	Sphingomonadales	Sphingomon- adaceae	Sphingomonas	Sphingomonas sp001464615
ARSTAG_ARBF_ 1_post_bin_27	Caulobacter_1	Bacteria	Proteo- bacteria	Alphaproteo- bacteria	Caulobacterales	Caulobacteraceae	Caulobacter	NA
ARSTAG_ARBF_ 1_post_bin_3	Alphaproteo- bacteria_2	Bacteria	Proteo- bacteria	Alphaproteo- bacteria	UBA9655	UBA9655	UBA9655	UBA9655 sp001767835
ARSTAG_ARBF_ 1_post_bin_31_1	Bosea_1	Bacteria	Proteo- bacteria	Alphaproteo- bacteria	Rhizobiales	Beijerinckiaceae	Bosea	Bosea sp001556025
ARSTAG_ARBF_ 1_post_bin_36_2	Hyphomicrobium_2	Bacteria	Proteo- bacteria	Alphaproteo- bacteria	Rhizobiales	Hyphomicrobiaceae	Hyphomicrobium	NA
ARSTAG_ARBF_ 1_post_bin_mycob_7	Mycobacterium_3	Bacteria	Actino- bacteriota	Actinomycetia	Mycobacteriales	Mycobacteriaceae	Mycobacterium	NA
ARSTAG_ARBF_ 12345_pre_bin_16_2	Sphingomonas_2	Bacteria	Proteo- bacteria	Alphaproteo- bacteria	Sphingomonadales	Sphingomon- adaceae	Sphingomonas	Sphingomonas sp001556185
ARSTAG_ARBF_ 12345_pre_bin_2_2	Sphingomonas_3	Bacteria	Proteo- bacteria	Alphaproteo- bacteria	Sphingomonadales	Sphingomon- adaceae	Sphingomonas	Sphingomonas sp002083435
ARSTAG_ARBF_ 12345_pre_bin_26	Obscuri- bacteraceae_1	Bacteria	Cyano- bacteria	Vampirovibrionia	Obscuribacterales	Obscuribacteraceae	Ga0077546	Ga0077546 sp001464165
ARSTAG_ARBF_ 12345_pre_bin_30	Obscuri- bacteraceae_2	Bacteria	Cyano- bacteria	Vampirovibrionia	Obscuribacterales	Obscuribacteraceae	QKMZ01	QKMZ01 sp014380105
ARSTAG_ARBF_ 12345_pre_bin_4_1	Methylo- bacterium_1	Bacteria	Proteo- bacteria	Alphaproteo- bacteria	Rhizobiales	Beijerinckiaceae	Methylobacterium	NA
ARSTAG_ARBF_ 12345_pre_bin_40_2	Rhizobiales_1	Bacteria	Proteo- bacteria	Alphaproteo- bacteria	Rhizobiales	UBA4765	UBA4765	UBA4765 sp002365175
ARSTAG_ARBF_ 12345_pre_bin_nitr_1	Nitrospira_3	Bacteria	Nitro- spirota	Nitrospiria	Nitrospirales	Nitrospiraceae	Nitrospira	NA
ARSTAG_ARBF_ 2_post_bin_13_1	Tabrizicola_1	Bacteria	Proteo- bacteria	Alphaproteo- bacteria	Rhodobacterales	Rhodobacteraceae	Tabrizicola	Tabrizicola sp005222785
ARSTAG_ARBF_ 2_post_bin_13_2	Hylemonella_1	Bacteria	Proteo- bacteria	Gammaproteo- bacteria	Burkholderiales	Burkholderiaceae	Hylemonella	Hylemonella sp003500955
ARSTAG_ARBF_ 2_post_bin_16_2	Reyranella_1	Bacteria	Proteo- bacteria	Alphaproteo- bacteria	Reyranellales	Reyranellaceae	Reyranella	NA
ARSTAG_ARBF_ 2_post_bin_29_1	Bryobacteraceae_2	Bacteria	Acido- bacteriota	Acidobacteriae	Bryobacterales	Bryobacteraceae	NA	NA
ARSTAG_ARBF_ 2_post_bin_3_2	Methylophilus_1	Bacteria	Proteo- bacteria	Gammaproteo- bacteria	Burkholderiales	Methylophilaceae	Methylophilus	Methylophilus sp001464125
ARSTAG_ARBF_ 2_post_bin_31_2	Sphingopyxis_1	Bacteria	Proteo- bacteria	Alphaproteo- bacteria	Sphingomonadales	Sphingomonadaceae	Sphingopyxis	Sphingopyxis sp013911715
ARSTAG_ARBF_ 2_post_bin_43_1	Mycobacterium_4	Bacteria	Actino- bacteriota	Actinomycetia	Mycobacteriales	Mycobacteriaceae	Mycobacterium	Mycobacterium sp002863225
ARSTAG_ARBF_ 2_post_bin_5_1	Nitrosomonas oligotropha_2	Bacteria	Proteo- bacteria	Gammaproteo- bacteria	Burkholderiales	Nitrosomonadaceae	Nitrosomonas	Nitrosomonas oligotropha
ARSTAG_ARBF_ 2_post_bin_8_1	Hyphomicrobium zavarzinii_1	Bacteria	Proteo- bacteria	Alphaproteo- bacteria	Rhizobiales	Hyphomicrobiaceae	Hyphomicrobium	Hyphomicrobium zavarzinii
ARSTAG_ARBF_ 3_post_bin_1	Phenylo- bacterium_1	Bacteria	Proteo- bacteria	Alphaproteo- bacteria	Caulobacterales	Caulobacteraceae	Phenylobacterium	Phenylobacterium sp004299445
ARSTAG_ARBF_ 3_post_bin_30_2	Bosea_2	Bacteria	Proteo- bacteria	Alphaproteo- bacteria	Rhizobiales	Beijerinckiaceae	Bosea	Bosea sp001556025
ARSTAG_ARBF_ 3_post_bin_38_2	Obscuribacter phosphatis_1	Bacteria	Cyano- bacteria	Vampirovibrionia	Obscuribacterales	Obscuribacteraceae	Obscuribacter	Obscuribacter phosphatis
ARSTAG_ARBF_ 3_post_bin_43_4	Burkholderiales_1	Bacteria	Proteo- bacteria	Gammaproteo- bacteria	Burkholderiales	SG8-41	Ga0077527	Ga0077527 sp001464815
ARSTAG_ARBF_ 3_post_bin_44_4	Mycobacterium_5	Bacteria	Actino- bacteriota	Actinomycetia	Mycobacteriales	Mycobacteriaceae	Mycobacterium	NA

ARSTAG_ARBF_3_post_bin_9_1	Firmicutes_1	Bacteria	Firmicutes	NA	NA	NA	NA	NA
ARSTAG_ARBF_3_post_bin_9_2	Alphaproteo-bacteria_3	Bacteria	Proteo-bacteria	Alphaproteo-bacteria	UBA9655	UBA9655	UBA9655	UBA9655 sp001897795
ARSTAG_ARBF_4_post_bin_0_2	Obscuri-bacteraceae_4	Bacteria	Cyano-bacteria	Vampirovibrionia	Obscuribacterales	Obscuribacteraceae	QKMZ01	NA
ARSTAG_ARBF_4_post_bin_1_2	Hyphomicrobium_1	Bacteria	Proteo-bacteria	Alphaproteo-bacteria	Rhizobiales	Hyphomicrobiaceae	Hyphomicrobium	Hyphomicrobium sp013141015
ARSTAG_ARBF_4_post_bin_1_3	Afipia_1	Bacteria	Proteo-bacteria	Alphaproteo-bacteria	Rhizobiales	Xanthobacteraceae	Afipia	Afipia broomeae
ARSTAG_ARBF_4_post_bin_12_1	Parvularculaceae_1	Bacteria	Proteo-bacteria	Alphaproteo-bacteria	Caulobacterales	Parvularculaceae	CV81	CV81 sp003506635
ARSTAG_ARBF_4_post_bin_18_3	Sphingomonas_4	Bacteria	Proteo-bacteria	Alphaproteo-bacteria	Sphingomonadales	Sphingomonadaceae	Sphingomonas	NA
ARSTAG_ARBF_4_post_bin_18_5	Bradyrhizobium_2	Bacteria	Proteo-bacteria	Alphaproteo-bacteria	Rhizobiales	Xanthobacteraceae	Bradyrhizobium	NA
ARSTAG_ARBF_4_post_bin_20	Planctomycetes_1	Bacteria	Plancto-mycetota	Planctomycetes	NA	NA	NA	NA
ARSTAG_ARBF_4_post_bin_21_1	Mycobacterium_6	Bacteria	Actino-bacteriota	Actinomycetia	Mycobacteriales	Mycobacteriaceae	Mycobacterium	NA
ARSTAG_ARBF_4_post_bin_22	Mycobacterium_7	Bacteria	Actino-bacteriota	Actinomycetia	Mycobacteriales	Mycobacteriaceae	Mycobacterium	NA
ARSTAG_ARBF_4_post_bin_26	Novosphing-obium_1	Bacteria	Proteo-bacteria	Alphaproteo-bacteria	Sphingomonadales	Sphingomon-adaceae	Novosphingobium	NA
ARSTAG_ARBF_4_post_bin_33	Nitrospira_4	Bacteria	Nitro-spirota	Nitrospiria	Nitrospirales	Nitrospiraceae	Nitrospira	NA
ARSTAG_ARBF_4_post_bin_7	Pirellulales_2	Bacteria	Plancto-mycetota	Planctomycetes	Pirellulales	Ga0077529	Ga0077529	Ga0077529 sp001464525
ARSTAG_ARBF_5_post_bin_12	Desulfobacterota binatia_1	Bacteria	Desulfo-bacterota	Binatia	UTPRO1	UTPRO1	UTPRO1	UTPRO1 sp002050235
ARSTAG_ARBF_5_post_bin_15_2	Novosphing-obium_2	Bacteria	Proteo-bacteria	Alphaproteo-bacteria	Sphingomonadales	Sphingomon-adaceae	Novosphingobium	Novosphingobium sp900117425
ARSTAG_ARBF_5_post_bin_20_5	Phycisphaerales_2	Bacteria	Plancto-mycetota	Phycisphaerae	Phycisphaerales	SM1A02	UBA4658	UBA4658 sp002405485
ARSTAG_ARBF_5_post_bin_21_2	Obscuri-bacteraceae_5	Bacteria	Cyano-bacteria	Vampirovibrionia	Obscuribacterales	Obscuribacteraceae	PALSA-1081	PALSA-1081 sp003963305
ARSTAG_ARBF_5_post_bin_21_3	Vampirovibrionia_1	Bacteria	Cyano-bacteria	Vampirovibrionia	NA	NA	NA	NA
ARSTAG_ARBF_5_post_bin_39_3	Hyphomicrobium facile_1	Bacteria	Proteo-bacteria	Alphaproteo-bacteria	Rhizobiales	Hyphomicrobiaceae	Hyphomicrobium	Hyphomicrobium facile
ARSTAG_ARBF_5_post_bin_43_2	Sphingopyxis_2	Bacteria	Proteo-bacteria	Alphaproteo-bacteria	Sphingomonadales	Sphingomonadaceae	Sphingopyxis	NA
ARSTAG_ARBF_5_post_bin_5	Mycobacterium_1	Bacteria	Actino-bacteriota	Actinomycetia	Mycobacteriales	Mycobacteriaceae	Mycobacterium	Mycobacterium sp001644575
ARSTAG_CONTROL_BFSLIDECONTROL_41_bin_18	Burkholderia_1	Bacteria	Proteo-bacteria	Gammaproteo-bacteria	Burkholderiales	Burkholderiaceae	Burkholderia	NA
ARSTAG_CONTROL_BFSLIDECONTROL_41_bin_20_3	Hyphomicrobium denitrificans_1	Bacteria	Proteo-bacteria	Alphaproteo-bacteria	Rhizobiales	Hyphomicrobiaceae	Hyphomicrobium	Hyphomicrobium denitrificans
ARSTAG_CONTROL_BFSLIDECONTROL_41_bin_meth_1	Methylo-bacterium_2	Bacteria	Proteo-bacteria	Alphaproteo-bacteria	Rhizobiales	Beijerinckiaceae	Methylobacterium	NA
ARSTAG_CONTROL_BFSLIDECONTROL_41_bin_n_1	Nitrospira_1	Bacteria	Nitro-spirota	Nitrospiria	Nitrospirales	Nitrospiraceae	Nitrospira	NA
ARSTAG_CONTROL_MOCK1E10_111821_bin_1	Listeria_1	Bacteria	Firmicutes	Bacilli	Lactobacillales	Listeriaceae	Listeria	NA
ARSTAG_CONTROL_MOCK1E10_111821_bin_1_2	Bacillus_1	Bacteria	Firmicutes	Bacilli	Bacillales	Bacillaceae	Bacillus	NA
ARSTAG_CONTROL_MOCK1E10_111821_bin_3	Pseudomonas aeruginosa_1	Bacteria	Proteo-bacteria	Gammaproteo-bacteria	Pseudomonadales	Pseudomonadaceae	Pseudomonas	Pseudomonas aeruginosa
ARSTAG_CONTROL_MOCK1E8_111821_bin_0	Staphylococcus_1	Bacteria	Firmicutes	Bacilli	Staphylococcales	Staphylococcaceae	Staphylococcus	NA
ARSTAG_CONTROL_MOCK1E8_111821_bin_1	Limosilacto-bacillus_1	Bacteria	Firmicutes	Bacilli	Lactobacillales	Lactobacillaceae	Limosilactobacillus	Limosilactobacillus fermentum
ARSTAG_CONTROL_MOCK1E8_111821_bin_9	Enterococcus_1	Bacteria	Firmicutes	Bacilli	Lactobacillales	Enterococcaceae	Enterococcus	Enterococcus faecalis
ARSTAG_TAPRES_TAPRES_23_bin_11	Alphaproteo-bacteria_1	Bacteria	Proteo-bacteria	Alphaproteo-bacteria	NA	NA	NA	NA
ARSTAG_TAPRES_TAPRES_23_bin_31_4	Methylocystis_1	Bacteria	Proteo-bacteria	Alphaproteo-bacteria	Rhizobiales	Beijerinckiaceae	Methylocystis	Methylocystis sp000188155
ARSTAG_TAPRES_TAPRES_23_bin_40_1	Mycobacterium iranicum_1	Bacteria	Actino-bacteriota	Actinomycetia	Mycobacteriales	Mycobacteriaceae	Mycobacterium	Mycobacterium iranicum

ARSTAG_TAPRES_ TAPRES_23_bin_8_1	Obscuri- bacteraceae_3	Bacteria	Cyano- bacteria	Vampirovibrionia	Obscuribacterales	Obscuribacteraceae	QKMZ01	QKMZ01 sp012965515
ARSTAG_TAPRES_ TAPRES_23_ bin_mycob_1_3	Mycobacterium_2	Bacteria	Actino- bacteriota	Actinomycetia	Mycobacteriales	Mycobacteriaceae	Mycobacterium	NA
ARSTAG_TAPRES_ TAPRES_23_ bin_unknown_1	Thiothrix_1	Bacteria	Proteo- bacteria	Gammaproteo- bacteria	Thiotrichales	Thiotrichaceae	Thiothrix	NA
ARSTAG_TAPRES_ TAPRES_40_bin_1_5	Rhodocyclaceae_1	Bacteria	Proteo- bacteria	Gammaproteo- bacteria	Burkholderiales	Rhodocyclaceae	Fen-999	Fen-999 sp003158255
ARSTAG_TAPRES_ TAPRES_40_bin_11	Rhodocyclaceae_2	Bacteria	Proteo- bacteria	Gammaproteo- bacteria	Burkholderiales	Rhodocyclaceae	66-26	66-26 sp001897745
ARSTAG_TAPRES_ TAPRES_40_bin_13_3	Gallionella_1	Bacteria	Proteo- bacteria	Gammaproteo- bacteria	Burkholderiales	Gallionellaceae	Gallionella	Gallionella sp001801105
ARSTAG_TAPRES_ TAPRES_40_bin_29_1	Gemmataceae_1	Bacteria	Plancto- mycetota	Planctomycetes	Gemmatales	Gemmataceae	UBA969	UBA969 sp003671255
ARSTAG_TAPRES_ TAPRES_40_bin_33_1	Gammaproteo- bacteria_1	Bacteria	Proteo- bacteria	Gammaproteo- bacteria	SURF-13	SURF-13	SURF-13	SURF-13 sp003599485
ARSTAG_TAPRES_ TAPRES_40_bin_35_2	Pirellulales_3	Bacteria	Plancto- mycetota	Planctomycetes	Pirellulales	UBA1268	QWPN01	QWPN01 sp014192425
ARSTAG_TAPRES_ TAPRES_41_bin_0_1	Rhodocyclaceae_3	Bacteria	Proteo- bacteria	Gammaproteo- bacteria	Burkholderiales	Rhodocyclaceae	Ga0074130	Ga0074130 sp001464495
ARSTAG_TAPRES_ TAPRES_41_bin_0_4	Rhodo- bacteraceae_1	Bacteria	Proteo- bacteria	Alphaproteo- bacteria	Rhodobacterales	Rhodobacteraceae	NA	NA
ARSTAG_TAPRES_ TAPRES_41_bin_1	Obscuribacter phosphatis_2	Bacteria	Cyano- bacteria	Vampirovibrionia	Obscuribacterales	Obscuribacteraceae	Obscuribacter	Obscuribacter phosphatis
ARSTAG_TAPRES_ TAPRES_41_bin_21_1	Hyphomicro- biaceae_1	Bacteria	Proteo- bacteria	Alphaproteo- bacteria	Rhizobiales	Hyphomicrobiaceae	DSSY01	DSSY01 sp011330955
ARSTAG_TAPRES_ TAPRES_41_bin_21_2	Hyphomicrobium_4	Bacteria	Proteo- bacteria	Alphaproteo- bacteria	Rhizobiales	Hyphomicrobiaceae	Hyphomicrobium	NA
ARSTAG_TAPRES_ TAPRES_41_bin_21_3	Hyphomicrobium_5	Bacteria	Proteo- bacteria	Alphaproteo- bacteria	Rhizobiales	Hyphomicrobiaceae	Hyphomicrobium	Hyphomicrobium sp002928395
ARSTAG_TAPRES_ TAPRES_41_bin_22_2	Chryseoglobus_1	Bacteria	Actino- bacteriota	Actinomycetia	Actinomycetales	Microbacteriaceae	Chryseoglobus	Chryseoglobus frigidaquae
ARSTAG_TAPRES_ TAPRES_41_bin_26_2	Lacibacter	Bacteria	Bacter- oidota	Bacteroidia	Chitinophagales	Chitinophagaceae	Lacibacter	Lacibacter cauensis
ARSTAG_TAPRES_ TAPRES_41_bin_27_2	Sphingomonas_5	Bacteria	Proteo- bacteria	Alphaproteo- bacteria	Sphingomonadales	Sphingomon- adaceae	Sphingomonas	NA
ARSTAG_TAPRES_ TAPRES_41_bin_28_3	Parvularculaceae_2	Bacteria	Proteo- bacteria	Alphaproteo- bacteria	Caulobacterales	Parvularculaceae	CV81	CV81 sp003506635
ARSTAG_TAPRES_ TAPRES_41_bin_33_1	Phycisphaerales_1	Bacteria	Plancto- mycetota	Phycisphaerae	Phycisphaerales	SM1A02	WM-009	WM-009 sp013822085
ARSTAG_TAPRES_ TAPRES_41_bin_nitro_3	Nitrospira_2	Bacteria	Nitro- spirota	Nitrospiria	Nitrospirales	Nitrospiraceae	Nitrospira	Nitrospira sp900299245

Supplementary Table 11: Quality Information for All Dereplicated Bins.

Dereplicated Genome Bin	Bin Figure Name	Bin Length (bp)	GC Content (%)	Contigs Count	N50	Completeness (%)	Redundancy (%)
ARSTAG_AR_ 4_27_bin_23_1	Xantho- bacteraceae_1	4619039	62.07	17	541953	100.00	0.00
ARSTAG_AR_ 4_27_bin_26_4	Hyphomicrobium_3	3095794	60.01	177	22480	94.37	0.00
ARSTAG_AR_ 4_27_bin_3_2	Nitrosomonas oligotropha_1	3148884	48.58	101	53228	94.37	0.00
ARSTAG_AR_ 4_27_bin_5_5	Rhodovarius_1	4820245	68.33	79	139498	100.00	0.00
ARSTAG_ARBF_ 1_post_bin_10	Bryobacteraceae_1	6879055	62.65	136	103824	97.18	2.82
ARSTAG_ARBF_ 1_post_bin_18_1	Pirellulales_1	7031808	68.82	264	38437	92.96	1.41
ARSTAG_ARBF_ 1_post_bin_2_3	Bradyrhizobium_1	7199065	63.01	87	140829	98.59	0.00
ARSTAG_ARBF_ 1_post_bin_26_2	Sphingomonas_1	3899856	61.75	45	184573	98.59	2.82
ARSTAG_ARBF_ 1_post_bin_27	Caulobacter_1	4134774	66.85	1255	4149	92.96	0.00
ARSTAG_ARBF_ 1_post_bin_3	Alphaproteo- bacteria_2	3694558	39.73	468	39025	91.55	8.45
ARSTAG_ARBF_ 1_post_bin_31_1	Bosea_1	5643005	66.54	118	78172	100.00	0.00
ARSTAG_ARBF_ 1_post_bin_36_2	Hyphomicrobium_2	4444842	64.20	20	689653	100.00	1.41
ARSTAG_ARBF_ 1_post_bin_mycob_7	Mycobacterium_3	5718300	67.29	917	10032	98.59	1.41
ARSTAG_ARBF_ 12345_pre_bin_16_2	Sphingomonas_2	3387346	64.19	69	96367	91.55	0.00
ARSTAG_ARBF_ 12345_pre_bin_2_2	Sphingomonas_3	3697639	67.11	870	5837	90.14	8.45
ARSTAG_ARBF_ 12345_pre_bin_26	Obscuri- bacteraceae_1	7124969	48.30	92	282249	95.77	7.04
ARSTAG_ARBF_ 12345_pre_bin_30	Obscuri- bacteraceae_2	6772201	47.43	53	525688	94.37	5.63
ARSTAG_ARBF_ 12345_pre_bin_4_1	Methylo- bacterium_1	5287748	65.40	130	109741	100.00	1.41
ARSTAG_ARBF_ 12345_pre_bin_40_2	Rhizobiales_1	3441437	61.05	426	13561	97.18	1.41
ARSTAG_ARBF_ 12345_pre_bin_nitr_1	Nitrospira_3	3580629	56.56	196	22378	91.55	0.00
ARSTAG_ARBF_ 2_post_bin_13_1	Tabrizicola_1	3828586	65.48	57	189099	100.00	0.00
ARSTAG_ARBF_ 2_post_bin_13_2	Hylemonella_1	3749190	65.54	42	171864	100.00	1.41
ARSTAG_ARBF_ 2_post_bin_16_2	Reyranella_1	9487268	66.23	73	242581	100.00	0.00
ARSTAG_ARBF_ 2_post_bin_29_1	Bryobacteraceae_2	6167450	63.66	73	137950	97.18	0.00
ARSTAG_ARBF_ 2_post_bin_3_2	Methylophilus_1	2641096	46.86	21	288431	100.00	1.41
ARSTAG_ARBF_ 2_post_bin_31_2	Sphingopyxis_1	3490294	62.74	15	546363	100.00	0.00
ARSTAG_ARBF_ 2_post_bin_43_1	Mycobacterium_4	5351921	66.14	104	107638	97.18	1.41
ARSTAG_ARBF_ 2_post_bin_5_1	Nitrosomonas oligotropha_2	3369947	48.60	369	14963	95.77	4.23
ARSTAG_ARBF_ 2_post_bin_8_1	Hyphomicrobium zavarzinii_1	4414071	61.97	146	45357	97.18	2.82
ARSTAG_ARBF_ 3_post_bin_1	Phenylo- bacterium_1	6297493	68.77	526	24592	100.00	5.63
ARSTAG_ARBF_ 3_post_bin_30_2	Bosea_2	4529685	66.98	36	239422	98.59	5.63

ARSTAG_ARBF_3_post_bin_38_2	Obscuribacter phosphatis_1	6367258	48.65	34	358159	94.37	2.82
ARSTAG_ARBF_3_post_bin_43_4	Burkholderiales_1	3882113	62.87	28	429866	100.00	1.41
ARSTAG_ARBF_3_post_bin_44_4	Mycobacterium_5	5518646	63.81	51	197964	98.59	1.41
ARSTAG_ARBF_3_post_bin_9_1	Firmicutes_1	2447424	41.31	115	71995	97.18	1.41
ARSTAG_ARBF_3_post_bin_9_2	Alphaproteo-bacteria_3	3237885	39.84	284	31779	94.37	1.41
ARSTAG_ARBF_4_post_bin_0_2	Obscuri-bacteraceae_4	6288689	50.21	29	366737	92.96	4.23
ARSTAG_ARBF_4_post_bin_1_2	Hyphomicrobium_1	3541694	59.81	16	761748	100.00	0.00
ARSTAG_ARBF_4_post_bin_1_3	Afipia_1	5038115	61.26	44	241866	100.00	2.82
ARSTAG_ARBF_4_post_bin_12_1	Parvularculaceae_1	3607200	64.57	63	117236	100.00	0.00
ARSTAG_ARBF_4_post_bin_18_3	Sphingomonas_4	2818854	67.34	43	133619	100.00	1.41
ARSTAG_ARBF_4_post_bin_18_5	Bradyrhizobium_2	6937557	63.29	125	81718	97.18	2.82
ARSTAG_ARBF_4_post_bin_20	Planctomycetes_1	7054827	72.39	187	65259	98.59	1.41
ARSTAG_ARBF_4_post_bin_21_1	Mycobacterium_6	6520233	66.83	167	57953	100.00	5.63
ARSTAG_ARBF_4_post_bin_22	Mycobacterium_7	7822085	65.61	188	66898	98.59	4.23
ARSTAG_ARBF_4_post_bin_26	Novosphing-obium_1	3288073	63.88	74	98654	100.00	0.00
ARSTAG_ARBF_4_post_bin_33	Nitrospira_4	3919319	56.17	778	6999	97.18	1.41
ARSTAG_ARBF_4_post_bin_7	Pirellulales_2	7044622	68.93	145	84107	98.59	4.23
ARSTAG_ARBF_5_post_bin_12	Desulfobacterota binatia_1	7054206	69.55	60	236306	98.59	4.23
ARSTAG_ARBF_5_post_bin_15_2	Novosphing-obium_2	4266691	65.46	42	203894	100.00	1.41
ARSTAG_ARBF_5_post_bin_20_5	Phycisphaerales_2	4286531	64.18	33	268898	98.59	0.00
ARSTAG_ARBF_5_post_bin_21_2	Obscuri-bacteraceae_5	7782369	50.22	48	1062029	94.37	4.23
ARSTAG_ARBF_5_post_bin_21_3	Vampirovibrionia_1	7985498	48.98	91	147763	95.77	2.82
ARSTAG_ARBF_5_post_bin_39_3	Hyphomicrobium facile_1	4467177	59.63	17	573520	100.00	0.00
ARSTAG_ARBF_5_post_bin_43_2	Sphingopyxis_2	4541130	64.47	67	134144	100.00	2.82
ARSTAG_ARBF_5_post_bin_5	Mycobacterium_1	6693803	66.99	169	76067	98.59	1.41
ARSTAG_CONTROL_BFSLIDECONTROL_41_bin_18	Burkholderia_1	8151006	66.55	62	227039	100.00	4.23
ARSTAG_CONTROL_BFSLIDECONTROL_41_bin_20_3	Hyphomicrobium denitrificans_1	3371528	59.52	51	130174	97.18	0.00
ARSTAG_CONTROL_BFSLIDECONTROL_41_bin_meth_1	Methylo-bacterium_2	6366832	69.52	269	42453	97.18	4.23
ARSTAG_CONTROL_BFSLIDECONTROL_41_bin_n_1	Nitrospira_1	3162924	58.41	857	5275	92.96	5.63
ARSTAG_CONTROL_MOCK1E10_111821_bin_1	Listeria_1	2866754	37.95	19	475945	98.59	4.23
ARSTAG_CONTROL_MOCK1E10_111821_bin_1_2	Bacillus_1	3960926	43.82	28	263361	92.96	0.00

ARSTAG_CONTROL_ MOCK1E10_ 111821_bin_3	Pseudomonas aeruginosa_1	6709546	66.27	83	215458	98.59	1.41
ARSTAG_CONTROL_ MOCK1E8_ 111821_bin_0	Staphylococcus_1	2842453	32.50	68	99416	98.59	1.41
ARSTAG_CONTROL_ MOCK1E8_ 111821_bin_1	Limosilacto-bacillus_1	1800900	51.29	78	40440	98.59	1.41
ARSTAG_CONTROL_ MOCK1E8_ 111821_bin_9	Enterococcus_1	2785332	37.52	36	215026	100.00	9.86
ARSTAG_TAPRES_ TAPRES_23_bin_11	Alphaproteo-bacteria_1	2237620	67.28	543	5205	94.37	2.82
ARSTAG_TAPRES_ TAPRES_23_bin_31_4	Methylocystis_1	3542705	64.25	72	97453	95.77	4.23
ARSTAG_TAPRES_ TAPRES_23_bin_40_1	Mycobacterium iranicum_1	6651069	65.93	111	142519	98.59	4.23
ARSTAG_TAPRES_ TAPRES_23_bin_8_1	Obscuri-bacteraceae_3	8013946	51.34	49	471261	95.77	5.63
ARSTAG_TAPRES_ TAPRES_23_ bin_mycob_1_3	Mycobacterium_2	7354236	66.57	180	92430	97.18	5.63
ARSTAG_TAPRES_ TAPRES_23_ bin_unknown_1	Thiothrix_1	3306466	51.37	1403	2647	90.14	8.45
ARSTAG_TAPRES_ TAPRES_40_bin_1_5	Rhodocyclaceae_1	3699494	63.87	143	36774	94.37	2.82
ARSTAG_TAPRES_ TAPRES_40_bin_11	Rhodocyclaceae_2	3595318	67.33	558	8423	92.96	2.82
ARSTAG_TAPRES_ TAPRES_40_bin_13_3	Gallionella_1	2754089	56.27	149	26182	94.37	0.00
ARSTAG_TAPRES_ TAPRES_40_bin_29_1	Gemmataceae_1	4500548	44.10	707	8513	91.55	0.00
ARSTAG_TAPRES_ TAPRES_40_bin_33_1	Gammaproteo-bacteria_1	1652173	60.35	543	3804	94.37	4.23
ARSTAG_TAPRES_ TAPRES_40_bin_35_2	Pirellulales_3	3711734	68.98	333	18762	100.00	0.00
ARSTAG_TAPRES_ TAPRES_41_bin_0_1	Rhodocyclaceae_3	3447979	62.00	61	189579	100.00	4.23
ARSTAG_TAPRES_ TAPRES_41_bin_0_4	Rhodo-bacteraceae_1	4502919	65.77	57	151583	100.00	1.41
ARSTAG_TAPRES_ TAPRES_41_bin_1	Obscuribacter phosphatis_2	6482763	53.07	990	8688	90.14	7.04
ARSTAG_TAPRES_ TAPRES_41_bin_21_1	Hyphomicro-biaceae_1	6804856	62.80	464	21777	95.77	5.63
ARSTAG_TAPRES_ TAPRES_41_bin_21_2	Hyphomicrobium_4	3725274	60.72	225	27441	90.14	0.00
ARSTAG_TAPRES_ TAPRES_41_bin_21_3	Hyphomicrobium_5	2971638	63.03	338	10367	91.55	7.04
ARSTAG_TAPRES_ TAPRES_41_bin_22_2	Chryseoglobus_1	2535481	71.14	22	173998	100.00	0.00
ARSTAG_TAPRES_ TAPRES_41_bin_26_2	Lacibacter	4076827	40.51	29	284946	98.59	1.41
ARSTAG_TAPRES_ TAPRES_41_bin_27_2	Sphingomonas_5	2861123	65.67	12	541427	100.00	1.41
ARSTAG_TAPRES_ TAPRES_41_bin_28_3	Parvularculaceae_2	3138522	63.14	47	150699	97.18	0.00
ARSTAG_TAPRES_ TAPRES_41_bin_33_1	Phycisphaerales_1	3706484	68.22	144	51728	97.18	1.41
ARSTAG_TAPRES_ TAPRES_41_bin_nitro_3	Nitrospira_2	3817891	58.59	86	88281	92.96	1.41

Supplementary Table 12: PERMANOVA of Various Parameters. Permutational analysis of variance (PERMANOVA) of Aitchison distances using various data subsets. Parameters considered included sample type (bulk, biofilm, tap reservoir), phase (Phase I, Phase II), and sample source (reactor 1, reactor 2, reactor 3, reactor 4, reactor 5, tap reservoir).

**Data Subgroup**	**Significant Parameters explaining the most variance**	**Not significant Parameters**
All samples	Phase: 27.2% (p=0.001)	Sample Source, Sample Type
AR and BF only	Sample Source 29.1% (p=0.003)<br>Phase: 20.5% (p=0.001)<br>Sample Type: 3.5% (p=0.043)	NA
AR only	Phase: 34.5% (p=0.001)<br>Sample Source: 26.2% (p=0.007)	NA
Phase I (AR and tapres)	NA	Sample Source, Sample Type
Phase I (AR only)	Sample Source: 58.1% (p= 0.023)	Day
Phase II (AR, BF, tapres)	NA	Sample Source, Sample Type
Phase II (AR and BF)	Sample Source: 70.0% (p= 0.001)<br>Sample Type: 9.4% (p=0.001)	NA
Phase II (AR and tapres)	Sample Source: 84.9% (p= 0.001)	Sample Type
Phase II (AR only)	Sample Source: 83.8% (p=0.001)	NA

Supplementary Table 13: Bins Identified as Eukaryotes.

Assembly	Bin	Total Size	GC Content	SCG Domain
ARSTAG_AR_4_27	bin_2	5.15	59.04%	eukarya
ARSTAG_AR_4_27	bin_27	6.93	59.50%	eukarya
ARSTAG_ARBF_4_post	bin_37	7.44	51.10%	eukarya
ARSTAG_ARBF_4_post	bin_6	15.19	52.54%	eukarya
ARSTAG_ARBF_12345_pre	bin_22	14.91	53.70%	eukarya
ARSTAG_ARBF_12345_pre	bin_27	6.15	52.12%	eukarya
ARSTAG_CONTROL_MOCK1E10_111821	bin_7	10.71	38.21%	eukarya
ARSTAG_CONTROL_MOCK1E10_111821	bin_4	9.93	48.18%	eukarya
ARSTAG_ARBF_3_post	bin_16	7.42	58.82%	eukarya
ARSTAG_CONTROL_MOCK1E10	bin_7	10.71	38.21%	eukarya
ARSTAG_CONTROL_MOCK1E10	bin_4	9.93	48.18%	eukarya
ARSTAG_ARBF_1_post	bin_6	6.6	59.04%	eukarya
ARSTAG_ARBF_1_post	bin_29	8.31	54.10%	eukarya

Supplementary Table 14: ARB Silva Classifications of 18S rRNA gene sequences.

Assembly	Silva Classification from 18S
ARSTAG_AR_4_27	Unclassified;
ARSTAG_ARBF_4_post	Unclassified;
ARSTAG_ARBF_4_post	Unclassified;
ARSTAG_CONTROL_MOCK1E10_111821	Eukaryota;Fungi;Dikarya;Basidiomycota;Agaricomycotina;Tremellomycetes;Tremellales;Cryptococcaceae;Cryptococcus;
ARSTAG_CONTROL_MOCK1E10_111821	Eukaryota;Fungi;Dikarya;Ascomycota;Saccharomycotina;Saccharomycetes;Saccharomycetales;Saccharomycetaceae;Saccharomyces;
ARSTAG_CONTROL_MOCK1E8_111821	Eukaryota;Fungi;Dikarya;Basidiomycota;Agaricomycotina;Tremellomycetes;Tremellales;Cryptococcaceae;Cryptococcus;

ARSTAG_CONTROL_MOCK1E8_111821	Eukaryota;Fungi;Dikarya;Ascomycota;Saccharomycotina;Saccharomycetes;Saccharomycetales;Saccharomycetaceae;Saccharomyces;
ARSTAG_TAPRES_TAPRES_23	Unclassified;
ARSTAG_TAPRES_TAPRES_23	Eukaryota;Viridiplantae;Streptophyta;Embryophyta;Tracheophyta;Spermatophyta;Magnoliophyta;Liliopsida; (plant)
ARSTAG_TAPRES_TAPRES_27	Unclassified;
ARSTAG_TAPRES_TAPRES_27	Unclassified;
ARSTAG_TAPRES_TAPRES_41	Eukaryota;
ARSTAG_TAPRES_TAPRES_41	Unclassified;

Supplementary Table 15: NCBI BLAST Classifications of 18S rRNA gene sequences.

Assembly	Blast prediction	Description	Taxid	Score	Cover	Ident	Len	Accession
ARSTAG_AR_4_27	Vexillifera bacillipedes strain TIL2 small subunit ribosomal R...	freshwater amoebida	1105345	2455	99%	96.65	2131	HQ687484.1
ARSTAG_ARBF_4_post	Saccamoeba limax small subunit ribosomal RNA gene, complete...	aerobic, marine or freshwater amoeba	136464	3216	99%	97.03	1908	AF293902.1
ARSTAG_ARBF_4_post	Vannella sp. strain CH88/I 18S small subunit ribosomal RNA gen...	freshwater amoeba	325078	3456	99%	98.62	1953	AY929912.1
ARSTAG_TAPRES_TAPRES_23	Pholoe pallida 18S ribosomal RNA gene, internal transcribed...	top BLAST hits all for segmented worms	328599	1291	99%	79.56	8266	KY753843.1
ARSTAG_TAPRES_TAPRES_23	Juncus effusus genome assembly, chromosome: 6	top BLAST hits all for plants/grasses	13579	2346	100%	99.69	8710358	OX326994.1

ARSTAG_TAPRES_TAPRES_27	Pholoe pallida 18S ribosomal RNA gene, internal transcribed...	top BLAST hits all for segmented worms	328599	1290	100%	79.54	8266	KY753843.1
ARSTAG_TAPRES_TAPRES_27	Falcomonas daucoides 18S ribosomal RNA gene, partial sequence	cryptophyte, marine blue-green algae	95528	1447	78%	91.66	1734	AF143943.1
ARSTAG_TAPRES_TAPRES_41	Poteriospumella sp. isolate NY1 small subunit ribosomal RNA...	golden algae	2003111	2796	100%	97.27	1786	MZ707558.1
ARSTAG_TAPRES_TAPRES_41	Uncultured eukaryote gene for 18S rRNA, partial sequence, clon...	top 10 BLAST hits all for uncultured eukaryote	100272	3120	99%	99.59	1711	LC109022.1
ARSTAG_CONTROL_MOCK1E10_111821	Cryptococcus neoformans var. grubii H99 chromosome 02		235443	3326	100%	100	1645092	CP047903.1
ARSTAG_CONTROL_MOCK1E10_111821	Saccharomyces cerevisiae strain NCIM3186 chromosome XII sequence		4932	3319	100%	100	1078087	CP011821.1
ARSTAG_CONTROL_MOCK1E8_111821	Cryptococcus neoformans var. grubii H99 chromosome 02		235443	3326	100%	100	1645092	CP047903.1
ARSTAG_CONTROL_MOCK1E8_111821	Saccharomyces cerevisiae strain NCIM3186 chromosome XII sequence		4932	3319	100%	100	1078087	CP011821.1
